# Effects of different dietary patterns on glucose management in type 1 diabetes: a systematic review and meta-analysis of randomized controlled trials

**DOI:** 10.1016/j.eclinm.2025.103222

**Published:** 2025-04-28

**Authors:** Jiayi Zeng, Miranda Beck, Afroditi Alexandra Barouti, Josefin E. Löfvenborg, Sofia Carlsson, Anna-Maria Lampousi

**Affiliations:** a Institute of Environmental Medicine, Karolinska Institutet, Stockholm, Sweden; b Department of Epidemiological Methods and Etiological Research, Leibniz Institute for Prevention Research and Epidemiology - BIPS, Bremen, Germany; c Department of Molecular Medicine and Surgery, Karolinska Institutet, Stockholm, Sweden; d Center for Diabetes, Academic Specialist Center, Stockholm, Sweden; e Department of Risk and Benefit Assessment, Swedish Food Agency, Uppsala, Sweden; f Department of Medicine Solna, Clinical Epidemiology Division, Karolinska Institutet, Stockholm, Sweden

**Keywords:** Type 1 diabetes, Dietary patterns, Glucose management, Meta-analysis, Randomized controlled trials

## Abstract

**Background:**

Effective glucose management is essential to prevent complications in type 1 diabetes. While nutrition therapy is crucial, the optimal diet remains uncertain. Our systematic review and meta-analysis synthesized evidence from randomized controlled trials (RCTs) on the impact of various diets on glucose management in type 1 diabetes.

**Methods:**

We systematically searched Medline, Embase, and Cochrane Library up to 11 November 2024 for RCTs on dietary patterns and glucose control outcomes in type 1 diabetes, including HbA1c, time in range (TIR), coefficient of variation (CV), hypoglycemia, insulin dose, and anthropometric characteristics. Two reviewers independently extracted data and assessed the risk of bias using the RoB-2 tool. We estimated summary mean differences (MD) with 95% CIs using random-effects models. The certainty of the evidence was evaluated using GRADE. The study protocol was registered in PROSPERO (CRD42023479252).

**Findings:**

Out of 5287 studies, 35 RCTs involving children, adolescents, and adults with type 1 diabetes were eligible. Higher-fiber diets (≥35 g/day, 5 RCTs) reduced HbA1c (MD −0.46%, 95% CI −0.93 to 0.00, I^2^ 65%) and hypoglycemia (MD −0.81 episodes/month, 95% CI −1.34 to −0.28, I^2^ 0%), with moderate and low certainty of evidence, respectively. Carbohydrate-restricted diets (≤45% energy, 20 RCTs) improved TIR (MD 3.84%, 95% CI 2.24–5.44, I^2^ 0%), CV (MD −3.24%, 95% CI −5.51 to −0.97, I^2^ 53%), and insulin needs (MD −5.63 U/day, 95% CI −9.51 to −1.74, I^2^ 70%), but not HbA1c, with low to moderate certainty of evidence. Higher-protein, low-glycemic index, gluten-free, Mediterranean, vegan, and intermittent fasting diets showed no effects on glucose management (1–6 RCTs), although certainty of evidence was low.

**Interpretation:**

Maintaining a high-fiber diet while restricting other carbohydrates may improve glycemic control in individuals with type 1 diabetes. Further investigation is needed into long-term effects and other diets.

**Funding:**

10.13039/501100004359Swedish Research Council, Swedish Diabetes Foundation, and the Strategic Research Programme in Diabetes at 10.13039/501100004047Karolinska Institutet.

**Research in context**

**Evidence before this study**

Nutritional therapy is crucial for managing type 1 diabetes, yet dietary guidelines rely on evidence from type 2 diabetes or the general population due to limited evidence specific to type 1 diabetes. We conducted a preliminary search of PubMed from its inception until October 2023 for meta-analyses of randomized controlled trials (RCTs) that evaluated the effects of dietary patterns on glucose management in type 1 diabetes. The identified meta-analyses suggested potential benefits of low-glycemic index, high-fiber, and carbohydrate-restricted diets. However, these analyses included mixed populations of people with type 1 and type 2 diabetes, focused on individual dietary patterns, or were based on weak evidence.

**Added value of this study**

This study represents the first systematic review and meta-analysis encompassing the entire body of literature on the effects of dietary patterns on glucose management in individuals with type 1 diabetes. It synthesizes data from 35 RCTs, covering the effects of eight dietary patterns. Evidence rated with moderate certainty suggests that higher-fiber and carbohydrate-restricted diets have beneficial effects on glycemic control in individuals with type 1 diabetes. The remaining dietary patterns, including higher-protein, low-glycemic index, gluten-free, Mediterranean, vegan, and intermittent fasting diets, showed no effects on glucose management, although the certainty of evidence was low.

**Implications of all the available evidence**

Our findings may help inform dietary guidelines for individuals with type 1 diabetes. A closer examination of diets high in fiber and low in other carbohydrates is warranted. This includes assessing potential adverse effects and impacts on other important outcomes, such as cardiometabolic and kidney function. Additionally, there is a clear need for further RCTs on other common dietary patterns, given the current weak evidence.

## Introduction

Type 1 diabetes is becoming increasingly common worldwide, with an estimated annual incidence rise of 3%.^1^ The condition is marked by insulin deficiency, requiring exogenous insulin treatment for survival.^2^ Additionally, frequent hyperglycemia raises the risk of vascular complications, making intensive glucose management crucial.^3^ However, only about 16% of individuals with type 1 diabetes achieve the recommended HbA1c target of 7% (53 mmol/mol),^4^ highlighting the need for improved care strategies.

Nutrition therapy is fundamental for managing type 1 diabetes, with current dietary guidelines recommending healthy dietary patterns, such as the Mediterranean diet, to reach glycemic targets.^5^^,^^6^ However, these guidelines are primarily based on evidence from individuals with type 2 diabetes, who generally exhibit greater insulin resistance, and less insulin deficiency compared to those with type 1 diabetes. Insulin therapy in type 1 diabetes may also influence nutritional intervention responses. The impact of dietary patterns on type 1 diabetes management is unclear, with scattered evidence often from mixed populations of people with type 1 and type 2 diabetes.^7^, ^8^, ^9^ A recent meta-analysis of randomized controlled trials (RCTs) suggested carbohydrate-restricted diets might improve glycemic control in type 1 diabetes, though the evidence was weak.^10^ Since then, several RCTs have been published, potentially strengthening the evidence base.^11^, ^12^, ^13^, ^14^

This systematic review and meta-analysis aims to identify and synthesize existing evidence from RCTs on the impact of dietary patterns on glucose management in type 1 diabetes. By focusing exclusively on type 1 diabetes and incorporating multiple metrics, including HbA1c, hypoglycemia, coefficient of variation (CV), time in range (TIR), insulin dose, and body measurements, this review seeks to inform dietary guidelines for type 1 diabetes.

## Methods

### Search strategy and selection criteria

The literature search was performed in Medline (Ovid), Embase, and Cochrane Library, including the Cochrane Central Register of Controlled Trials (CENTRAL), by a librarian at Karolinska Institutet. Databases were searched from inception and the language was restricted to English. The search was initially performed on 21 December 2023 and was updated on 11 November 2024. Conference/congress papers, editorials, interviews, letters, as well as animal studies, were excluded. The detailed search strategy is described in Supplementary Tables S1–S3. Studies in the reference lists of eligible articles and relevant systematic reviews and meta-analyses were also screened for eligibility.

Studies were included if they met the following criteria: 1) RCT design; 2) Children, adolescents, or adults with type 1 diabetes; 3) Any dietary pattern intervention, defined as the totality of all foods and beverages consumed over a given period of time,^5^ but not interventions exclusively based on dietary supplements, single foods, or single meals; 4) The comparator was usual care, standard diet, or another dietary pattern; 5) The outcome was HbA1c, time below range (TBR, <3.9 mmol/L), time above range (TAR, >10.0 mmol/L), TIR (3.9–10.0 mmol/L),^15^ CV, number of hypoglycemic episodes, insulin dose, waist circumference, or body mass index (BMI). There was no restriction on the duration of the interventions.

The systematic review and meta-analysis was registered in PROSPERO (CRD42023479252) and follows the Preferred Reporting Items for Systematic Reviews and Meta-Analyses (PRISMA) guidelines.^16^

### Screening and data extraction

Two investigators (JZ and MB) independently screened the studies for eligibility and extracted relevant data. Any disagreements were resolved after consultation with a third investigator (AML). The screening process was conducted in two stages using Rayyan, a web-based tool for systematic reviews.^17^ The titles and abstracts were screened first and then the potentially eligible articles were fully examined. The information extracted from the eligible articles included: name of first author, year of publication, year of intervention, journal, country, trial name, trial design (parallel, cross-over), number of participants in each arm, sex, age at baseline, diabetes duration, intervention (type and duration), comparator, outcome, outcome assessment methods, comorbidities, drop out and loss to follow-up rates, follow-up time, mean with SD or, if not available, median with IQR at baseline and the end of follow-up for each outcome, and funding information. In case of unreported data, the study authors were contacted.

### Risk of bias assessment

The risk of bias among eligible studies was assessed independently by two investigators (JZ and MB) by using the Risk of Bias in randomized trials (RoB 2) tool.^18^ Potential disagreements were resolved after consultation with a third investigator (AML). RoB 2 covers five sources of bias in RCTs, including the randomization process, deviations from intended interventions, missing data, outcome measurement, and selection of reported result. Each domain is rated as having “low”, “some concerns”, or “high risk” of bias, and the overall risk of bias comes from the domain with the highest risk of bias.

### Statistical analysis

A random-effects model was used for obtaining summary mean differences (MDs) and 95% CIs of the prespecified outcomes in relation to different dietary patterns. When outcomes were only described as medians with IQR (n = 4), we assumed mean ≈ median and SD ≈ IQR/1.35. The Cochran’s Q test was used to assess the heterogeneity between the included studies and the percentage of observed variance was estimated with the I^2^ statistic. Funnel plots were visually inspected for publication bias when at least five studies were available, and Egger's test was conducted when at least ten studies were included.

In the main analyses, participants were combined irrespective of age. Subgroup and meta-regression analyses were performed for meta-analyses with at least five studies and substantial heterogeneity (I^2^ > 50%). Subgroups were pre-specified and formulated based on age (children/adolescents and adults), duration of intervention (1–6 weeks, 6–12 weeks and ≥12 weeks), composition of carbohydrate-restricted diets (<26% or 26–45% energy, substitution with protein, saturated, unsaturated, or unspecified fat), composition of higher-fiber diets (same or higher carbohydrate content than control group) and risk of bias (low, some concerns, high). The influence of the percentage of energy from carbohydrates in carbohydrate-restricted diets, and the difference in this percentage between intervention and control groups, were assessed in meta-regression analyses. All statistical analyses were performed with Stata Statistical Software Release 17 (StataCorp).

### Certainty of evidence assessment

The Grading of Recommendations, Assessment, Development, and Evaluations (GRADE) framework was used independently by two investigators (JZ and AML) to evaluate the certainty of meta-evidence.^19^ Any disagreements were resolved through consensus. This assessment starts by assigning a high level of certainty to meta-analyses of RCTs. The certainty of evidence can be downgraded if there is risk of bias, imprecision, inconsistency, indirectness, or publication bias. Conversely, it can be upgraded if there is a substantial effect, a dose-response relationship, or if all plausible biases would reduce an association. Ultimately, the certainty of evidence is classified into one of four levels: “very low”, “low”, “moderate”, or “high”.

### Ethics

Ethical approval was not required for this meta-analysis as it retrieved and synthesized summary-level data from previously published studies.

### Role of the funding source

The supporting sources had no role in study design, collection, analysis, and interpretation of data, writing of the report, and did not impose any restrictions regarding the publication of the report.

## Results

### Study characteristics

**Fig. 1:**
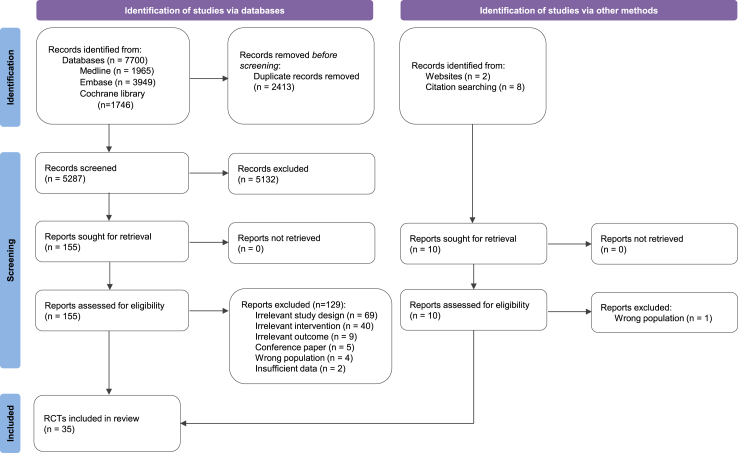
Flow diagram of study selection.

Of the 5287 articles that were initially screened, 35 fulfilled the eligibility criteria (Fig. 1). Table 1 describes all eligible studies,^11^, ^12^, ^13^, ^14^^,^^20^, ^21^, ^22^, ^23^, ^24^, ^25^, ^26^, ^27^, ^28^, ^29^, ^30^, ^31^, ^32^, ^33^, ^34^, ^35^, ^36^, ^37^, ^38^, ^39^, ^40^, ^41^, ^42^, ^43^, ^44^, ^45^, ^46^, ^47^, ^48^, ^49^, ^50^ and Supplementary Table S4 lists the studies that were excluded after full-text review (n = 130), along with reasons for their exclusion. Most of the included studies were conducted in Europe (n = 16), followed by North America (n = 11), Oceania (n = 4), Asia (n = 3), and Africa (n = 1). Participants were mainly adults, with only seven studies specifically focusing on children or adolescents. All studies, except for one that focused exclusively on females,^33^ included both males and females. None of the studies reported the inclusion of pregnant women. Most studies were rated as having “some concerns” (n = 18), followed by “low risk” (n = 12) and “high risk” (n = 5) of bias (Supplementary Fig. S1). The risk of bias was primarily due to deviations from intended interventions. Because the interventions were not blinded, concerns arose regarding adherence and unbalanced co-interventions (e.g., energy intake) across arms. Consequently, the domain of deviations from intended interventions was rated as “some concerns” when authors reported poor or no information on adherence (n = 9) or when the two arms were not isocaloric (n = 9). It was rated as “high risk” if both scenarios occurred.

**Table 1:** Characteristics of eligible studies.

Study	N	Mean age	Male/Female	Mean T1D duration	Country	Trial duration	Assessed diet	Comparison diet	Outcomes	Risk of bias
**Carbohydrate-restricted versus non-carbohydrate-restricted diet**										
Simpson 1979^20^^a^	11	40	5/6	18 years	UK	6 weeks	Diet instruction: 40% carbohydrates, 15% protein, and 45% fat. Ratio of polyunsaturated to other fatty acids 1:3.	Diet instruction: 60% carbohydrates, 15% protein, and 25% fat. Ratio of polyunsaturated to other fatty acids 1:1. Isocaloric.	HbA1c	Some concerns
Simpson 1981^21^^a^	9	41.2	4/5	11 years	UK	6 weeks	Personalized diet plan: 40% carbohydrates, 20% protein, 40% fat, and 18 g fiber per day. Ratio of polyunsaturated to other fatty acids 1:3.	Personalized diet plan: 60% carbohydrates, 22% protein, 18% fat, and 100 g fiber per day. Ratio of polyunsaturated to other fatty acids 1:1. Isocaloric.	HbA1c	Low
Anderson 1991^22^^a^	10	47.2	9/1	18 years	USA	4 weeks	Meal provision in hospital: 39% carbohydrates (50% simple and 50% complex) 20% protein, 41% fat (20% monounsaturated). With 500 mg cholesterol, and 10 g dietary fiber per day.	Meal provision in hospital: 70% carbohydrates (25% simple and 75% complex), 20% protein, 10% fat with 50 mg cholesterol, and 70 g dietary fiber per day. Isocaloric.	HbA1c	Some concerns
Dullaart 1993^23^^a^	30	40.9	27/3	20 years	The Netherlands	2 years	Diet instruction: 41% carbohydrates, 18% protein, and 41% fat.	Diet instruction: 48% carbohydrates, 13% protein (0.6 g/kg/day), and 39% fat. Isocaloric.	HbA1c/Insulin dose	Some concerns
Raal 1994^24^^a^	22	26.4	8/14	20 years	South Africa	6 months	Personalized diet plan in hospital: ≥1.6 g/kg/day protein, 30% fat, and remaining carbohydrate (<40%).	Personalized diet plan in hospital: 0.8 g/kg/day protein, 30% fat, and remaining carbohydrate (53%). Isocaloric.	HbA1c	Low
Donaghue 2000^25^	23	16.9	–	9 years	Australia	12 weeks	Diet instruction: 42% carbohydrates, 15% protein, and 43% fat (20% from monounsaturated fat).	Diet instruction: 55% carbohydrates, 15% protein, and 30% fat. Non-isocaloric.	HbA1c/Insulin dose	High
Georgopoulos 2000^26^^a^	19	29	11/8	13 years	USA	4 weeks	Meal provision: 45% carbohydrates, 15% protein, and 40% fat (25% monounsaturated, 6% polyunsaturated and 9% saturated).	Meal provision: 61% carbohydrates, 15% protein, and 24% fat (9% monounsaturated, 6% polyunsaturated and 9% saturated). Non-isocaloric.	HbA1c/Insulin dose	High
Strychar 2003^27^	26	39	15/11	19 years	Canada	8 weeks	Diet instruction: 43%–46% carbohydrates, 15%–18% protein, and 37%–40% fat (17%–20% monounsaturated).	Diet instruction: 54%–57% carbohydrates, 15%–18% protein, and 27%–30% fat (10%–13% monounsaturated). Isocaloric.	HbA1c/Insulin dose/BMI	Low
Strychar 2009^28^^a^	30	37.9	–	17 years	Canada	6 months	Diet instruction: 43–46% carbohydrates, 37–40% fat (20% monounsaturated).	Diet instruction: 54–57% carbohydrates, 27–30% fat (10% monounsaturated). Isocaloric.	HbA1c/BMI	Low
Krebs 2016^29^	10	44.6	7/3	22 years	New Zealand	12 weeks	Diet instruction: 50–75 g/day carbohydrates.	Diet instruction: normal diet, around 200 g/day carbohydrates. Non-isocaloric.	HbA1c/Insulin dose/BMI	Some concerns
Ranjan 2017^30^	10	48	6/4	23 years	Denmark	1 week	Personalized diet plan: 10% carbohydrates, 30% protein, and 60% fat. Carbohydrates <50 g/day.	Personalized diet plan: 51% carbohydrates, 21% protein, and 28% fat. Carbohydrates >250 g/day. Isocaloric.	HbA1c/TIR/TBR/TAR/CV/Insulin dose/BMI	Some concerns
Schmidt 2019^31^	14	44	6/8	19 years	Denmark	12 weeks	Personalized diet plan: <100 g per day carbohydrates.	Personalized diet plan: >250 g per day carbohydrates. Isocaloric.	HbA1c/TIR/TBR/TAR/CV/Insulin dose/Waist circumference	Some concerns
Dimosthenopoulos 2021^32^	15	36.1	5/10	12 years	Greece	1 week	Diet instruction: 20% carbohydrates, 40% protein, and 40% fat (>15% monounsaturated).	Diet instruction: 50% carbohydrates, 20% protein, and 30% fat. Isocaloric.	TIR/TAR/TBR/CV/Insulin dose/Waist circumference	Low
Öz 2021^33^	28	16.1	0/28	6 years	Turkey	10 weeks	Diet instruction: 25% carbohydrates, 25% protein, and 50% fat.	Diet instruction: 55% carbohydrates, 15% protein, and 30% fat. Isocaloric.	TIR/TAR/TBR/Insulin dose	Some concerns
Duffus 2022^34^	26	15.7	13/13	8 years	USA	12 weeks	Diet instruction: 25% carbohydrates. No instruction for protein and fat.	Diet instruction: 50% carbohydrates. No instruction for protein and fat. Isocaloric.	HbA1c	Some concerns
Igudesman 2022^35^	28	25.4	8/20	–	USA	12 weeks	Diet instruction: 15–20% carbohydrates (45–75 g/day depending on energy requirements) and <10% of fat from saturated fat.	Diet instruction: Flexibility related to % of calories from carbohydrates and protein. <30% fat and <10% fat from saturated fat. Isocaloric.	HbA1c/TIR/TAR/TBR/CV	Low
Isaksson 2024^11^	50	47.6	25/25	25 years	Sweden	4 weeks	Diet instruction: 30% carbohydrates, 20% protein, and 50% fat (mainly unsaturated oils).	Diet instruction: 50% carbohydrates, 20% protein, and 30% fat. Isocaloric.	TIR/TAR/TBR/Insulin dose	Low
Levran 2024^12^	40	18	6/14	10 years	Israel	6 months	Diet instruction: 15–20% carbohydrates, 33% protein, and 58% fat (mainly unsaturated).	Diet instruction: 40–50% carbohydrates, 25% protein, and 35% fat. Non-isocaloric.	HbA1c/TIR/CV/Insulin dose/BMI	Some concerns
Neuman 2024^13^	35	14.5	15/20	5 years	Czechia	5 weeks	Meal provision: 15% carbohydrates.	Meal provision: 35–45% carbohydrates. Isocaloric.	TIR/TAR/TBR/CV/Insulin dose/BMI	Low
Kristensen 2024^14^	12	50	8/4	25 years	Denmark	1 week	Diet instruction: 19% carbohydrates, 19% protein, and 62% fat (mainly from dairy products).	Diet instruction: 48% carbohydrates, 19% protein, and 33% fat. Isocaloric.	TIR/TAR/TBR/CV/Insulin dose/Hypoglycemic episodes	Some concerns
**Higher-protein versus lower-protein diet**										
Ciavarella 1987^36^^a^	16	37.1	9/7	18 years	Italy	4.5 months	Diet instruction: 1.44 g/kg/day protein.	Diet instruction: 0.71 g/kg/day protein. Isocaloric, same carbohydrate, supplementation of fat.	HbA1c/Insulin dose	Low
Dullaart 1993^23^^a^	30	40.9	27/3	20 years	The Netherlands	2 years	Diet instruction: 41% carbohydrates, 18% protein, and 41% fat.	Diet instruction: 48% carbohydrates, 13% protein (0.6 g/kg/day), and 39% fat. Isocaloric.	HbA1c/Insulin dose	Some concerns
Raal 1994^24^^a^	22	26.4	8/14	20 years	South Africa	6 months	Personalized diet plan in hospital: ≥1.6 g/kg/day protein, 30% fat, and remaining carbohydrate.	Personalized diet plan in hospital: 0.8 g/kg/day protein, 30% fat, and remaining carbohydrate. Isocaloric.	HbA1c	Low
Hansen 2002^37^^a^	82	40.5	53/29	28 years	Denmark	4 years	Diet instruction: 1 g/kg/day protein.	Diet instruction: 0.6 g/kg/day protein. Isocaloric.	HbA1c	High
Rosenfalck 2006^38^	13	34.9	6/7	10 years	Denmark	12 weeks	Meal provision: 55% carbohydrates, 20% protein, and 25% fat.	Meal provision: 55% carbohydrates, 15% protein, and 30% fat. Isocaloric.	HbA1c/Insulin dose	Some concerns
Öz 2021^33^	28	16.1	0/28	6 years	Turkey	10 weeks	Diet instruction: 25% carbohydrates, 25% protein, and 50% fat.	Diet instruction: 55% carbohydrates, 15% protein, and 30% fat. Isocaloric.	TIR/TAR/TBR/Insulin dose	Some concerns
**Higher-fiber versus lower-fiber diet**										
Simpson 1981^21^	9	41.2	4/5	11 years	UK	6 weeks	Personalized diet plan: 60% carbohydrates, 22% protein, 18% fat, and 100 g fiber per day.	Personalized diet plan: 40% carbohydrates, 20% protein, 40% fat, and 18 g fiber per day. Isocaloric.	HbA1c	Low
Kinmonth 1982^39^	10	14.1	6/4	5 years	UK	6 weeks	Diet instruction: 55% carbohydrates, 15% protein, 30% fat. Half carbohydrates as whole grains (wholemeal bread and cereals) and about half from fresh fruit and vegetables including dried beans. Supplied 3 g of dietary fiber for every 100 calories.	Diet instruction: 55% carbohydrates, 15% protein, 30% fat. Refined grains-such as white bread and cereals-supplied about two-thirds of the total carbohydrates, and the remaining one-third came from processed fruits and vegetables, milk, and yoghurt. Supplied 1 g of dietary fiber for every 100 calories. Isocaloric.	HbA1c/Hypoglycemic episodes/Insulin dose	Some concerns
Harold 1985^40^	4	22	2/2	–	USA	6 weeks	Diet instruction: normal daily intake 40% carbohydrates supplied with 78 g of wheat bran per day, and >50 g of fiber per day.	Diet instruction: 40% carbohydrates, <30 g fiber per day. Isocaloric.	HbA1c	Low
Anderson 1991^22^	10	47.2	9/1	18 years	USA	4 weeks	Meal provision in hospital: 70% carbohydrates (25% simple and 75% complex), 20% protein, and 10% fat, and 70 g of fiber per day.	Meal provision in hospital: 39% carbohydrates (50% simple and 50% complex) 20% protein, 41% fat with 500 mg cholesterol, and 10 g dietary fiber per day. Isocaloric.	HbA1c	Some concerns
Giacco 2000^41^	54	28.2	21/33	10 years	Italy	6 months	Diet instruction: fiber 50 g/day, 70% glycemic index; 20% protein, 30% fat, and 50% carbohydrates.	Diet instruction: fiber 15 g/day, 90% glycemic index; 20% protein, 30% fat, and 50% carbohydrates. Isocaloric.	HbA1c/Insulin dose/Hypoglycemic episodes	Low
**Low-glycemic index versus high-glycemic index diet**										
Kinmonth 1982^39^	10	14.1	6/4	5 years	UK	6 weeks	Diet instruction: 55% carbohydrates, 15% protein, 30% fat. Half carbohydrates as whole grains (wholemeal bread and cereals) and about half from fresh fruit and vegetables including dried beans. Supplied 3 g of dietary fiber for every 100 calories.	Diet instruction: 55% carbohydrates, 15% protein, 30% fat. Refined grains-such as white bread and cereals-supplied about two-thirds of the total carbohydrates, and the remaining one-third came from processed fruits and vegetables, milk, and yoghurt. Supplied 1 g of dietary fiber for every 100 calories. Isocaloric.	HbA1c/Hypoglycemic episodes/Insulin dose	Some concerns
Collier 1988^42^	7	12	6/1	3 years	Canada	6 weeks	Diet instruction: exchange list indicating which foods to be substituted for low glycemic index foods, 47% carbohydrates	Diet instruction: regular diet, 49% carbohydrates. Isocaloric.	HbA1c	Some concerns
Venhaus 1988^43^	10	27	8/2	13 years	Germany	6 weeks	Diet instruction: refined carbohydrates were to be avoided, whole grain products, leguminous, seeds, fruits, and vegetables were to be chosen. 40% carbohydrates	Diet instruction: whole grain products were to be avoided, and the intake of vegetables and fruits was limited to one serving of processed vegetables per day and less than five servings of fresh fruit per week. 41% carbohydrates. Non-isocaloric.	HbA1c/Insulin dose/Hypoglycemic episodes	Some concerns
Giacco 2000^41^	54	28.2	21/33	10 years	Italy	6 months	Diet instruction: fiber 50 g/day, 70% glycemic index; 20% protein, 30% fat, and 50% carbohydrates.	Diet instruction: fiber 15 g/day, 90% glycemic index; 20% protein, 30% fat, and 50% carbohydrates. Isocaloric.	HbA1c/Insulin dose/Hypoglycemic episodes	Low
Gilbertson 2001^44^	104	10.5	52/52	4 years	Australia	1 year	Diet instruction: tables for low glycemic index diet instruction. 49% carbohydrates	Diet instruction: tables for high glycemic index diet instruction. 49% carbohydrates. Isocaloric.	HbA1c/Insulin dose/Hypoglycemic episodes	Some concerns
Marquard 2011^45^^a^	16	10.2	6/10	3 years	Germany	12 weeks	Diet instruction: Avoid consuming carbohydrate carriers (pasta, rice), sweets, light bread and biscuits made with white flour, consume whole grain only. 60% carbohydrates	Diet instruction: Plenty of plant fruits (vegetables, fruits, cereals), moderately animal food (milk, dairy products, meat, sausage, eggs), occasionally fat and sugar-rich food (sweets), >50% whole grain. 62% carbohydrates. Isocaloric.	HbA1c	Some concerns
**Gluten-free diet versus non-gluten-free diet**										
Kaur 2020^46^	30	26.7	13/17	–	India	1 year	Diet instruction: safe/unsafe food lists.	Diet instruction: normal gluten-containing diet. Non-isocaloric.	HbA1c/Insulin dose/Hypoglycemic episodes/BMI	Some concerns
Mahmud 2020^47^	51	8–45	–	–	Canada	1 year	Diet instruction: gluten-free diet.	Diet instruction: normal gluten-containing diet. Non-isocaloric.	HbA1c/Insulin dose/BMI/Waist circumference	High
**Mediterranean versus non-Mediterranean diet**										
Fortin 2018^48^	28	50.9	16/12	27 years	Canada	6 months	Diet instruction: Mediterranean principles and recipes.	Diet instruction: limitation of total fat intake. Isocaloric.	HbA1c/Hypoglycemic episodes/BMI/Waist circumference	Low
Dimosthenopoulos 2021^32^	15	36.1	5/10	12 years	Greece	1 week	Diet instruction: use olive oil and nuts as the main sources of fat, low glycemic index (<60) foods, such as pulses, vegetables, cereals, and pasta, two servings of fish per week, and limited intake of red or processed meats. 40% carbohydrates, 25% protein, and 35% fat.	Diet instruction: 50% carbohydrates, 20% protein, and 30% fat. Isocaloric.	CV/TIR/TAR/TBR/Insulin dose/Waist circumference	Low
Igudesman 2022^35^	22	26.5	8/20	–	USA	12 weeks	Diet instruction: use of olive oil, increased consumption of plant-based foods, whole grains, and fish, reduced red meat consumption; avoidance of high-sugar baked goods and sugar-sweetened beverages, and moderate consumption of red wine among those who drink alcohol.	Diet instruction: <30% fat and <10% saturated fat. Non-isocaloric	HbA1c/CV/TAR/TIR/TBR	Low
Levran 2024^12^^a^	40	18	6/14	10 years	Israel	6 months	Diet instruction: diet rich in vegetables and low in red meat, with poultry and fish preferred to beef and lamb. The primary sources of added fat were 30–45 g of olive oil and a handful of nuts (5–7 nuts <20 g) per day. 40–50% carbohydrates, 25% protein, and 35% fat.	Diet instruction: 15–20% carbohydrates, 33% protein, and 58% fat. Non-isocaloric.	HbA1c/TIR/CV/Insulin dose/BMI	Some concerns
**Intermittent fasting versus continuous energy restriction diet**										
Overland 2018^49^	10	46.9	2/8	28 years	Australia	12 weeks	Meal provision and diet instruction: 600 calories on two 24-h periods a week, consisting of three meal replacement products. Participants were encouraged to eat to appetite on the remaining days of the week.	Personalized diet plan: restricted energy intake by 30% relative to weight maintenance energy needs. Non-isocaloric.	HbA1c/Insulin dose/Hypoglycemic episodes	High
**Vegan versus portion-controlled diet**										
Kahleova 2024^50^	35	49.5	28/7	–	USA	12 weeks	Diet instruction: diet (75% carbohydrates, 15% protein, and 10% fat) consisting of vegetables, grains, legumes, and fruits, with no limits on calories or carbohydrates. Participants were instructed to avoid animal products and added fats and to favor foods with a low glycemic index.	Diet instruction: reduced daily energy intake by 500–1000 kcal/day for overweight participants (BMI >25 kg/m^2^) and kept carbohydrate intake stable over time. 60–70% carbohydrate and monounsaturated fats, 15–20% protein, and <7% saturated fat and contained ≤200 mg/day of cholesterol. Non-isocaloric.	HbA1c/Insulin dose/BMI/CV/TIR	Some concerns

a Studies where the intervention and comparator groups were reversed. CI: confidence interval; BMI: body mass index; CV: coefficient of variation; TIR: time in range; TBR: time below range; TAR: time above range; T1D: type 1 diabetes.

Based on the 35 eligible RCTs, the dietary pattern comparisons assessed in this study include carbohydrate-restricted (≤45% energy) versus non-carbohydrate-restricted (>45% energy, with one study at 35–45%, n = 20),^11^, ^12^, ^13^, ^14^^,^^20^, ^21^, ^22^, ^23^, ^24^, ^25^, ^26^, ^27^, ^28^, ^29^, ^30^, ^31^, ^32^, ^33^, ^34^, ^35^ higher-protein (>15% energy) versus lower-protein (≤15% energy, n = 6),^23^^,^^24^^,^^33^^,^^36^, ^37^, ^38^ higher-fiber (≥35 g/day) versus lower-fiber (<35 g/day, n = 5),^21^^,^^22^^,^^39^, ^40^, ^41^ low-glycemic index versus high-glycemic index (except for one study that used a healthy control diet, n = 6),^39^^,^^41^, ^42^, ^43^, ^44^, ^45^ gluten-free versus non-gluten-free (n = 2),^46^^,^^47^ Mediterranean versus non-Mediterranean (n = 4),^12^^,^^32^^,^^35^^,^^48^ intermittent fasting versus continuous energy restriction (n = 1),^49^ and vegan versus portion-controlled (n = 1) diets.^50^ The definitions of dietary patterns were derived from the identified studies and were not pre-specified. In some cases, an intervention could fit into multiple definitions. For example, some studies primarily focused on protein restriction could fit both the higher-protein comparison and the carbohydrate restriction comparison if the intervention and control groups were reversed. The cut-offs for macronutrient intakes and fiber were based on previously suggested definitions (carbohydrate-restricted diets)^51^ or recommended intakes (protein and fiber).^6^ The interventions were primarily implemented through instructional methods, with durations ranging from one week to four years. Additionally, five studies included the provision of meals, with durations ranging from four to twelve weeks, consistent with the majority of the interventions (Table 1).

The outcomes that were assessed included HbA1c (n = 30), insulin dose (n = 23), number of hypoglycemic episodes per month (n = 7), BMI (n = 10), TIR (n = 10), TBR (n = 8), TAR (n = 8), CV (n = 8), and waist circumference (n = 4). We were able to meta-analyze the effects of all dietary patterns on HbA1c and insulin dose, except for intermittent fasting and vegan diet, which had only one available study. Effects on other outcomes could only be meta-analyzed for some of the interventions. Forests plots for each meta-analysis are presented in Supplementary Figs. S2–S28, funnel plots in Supplementary Figs. S29–S38 and the certainty of evidence assessment is shown in Supplementary Table S5.

### Carbohydrate-restricted versus non-carbohydrate-restricted diet

**Fig. 2:**
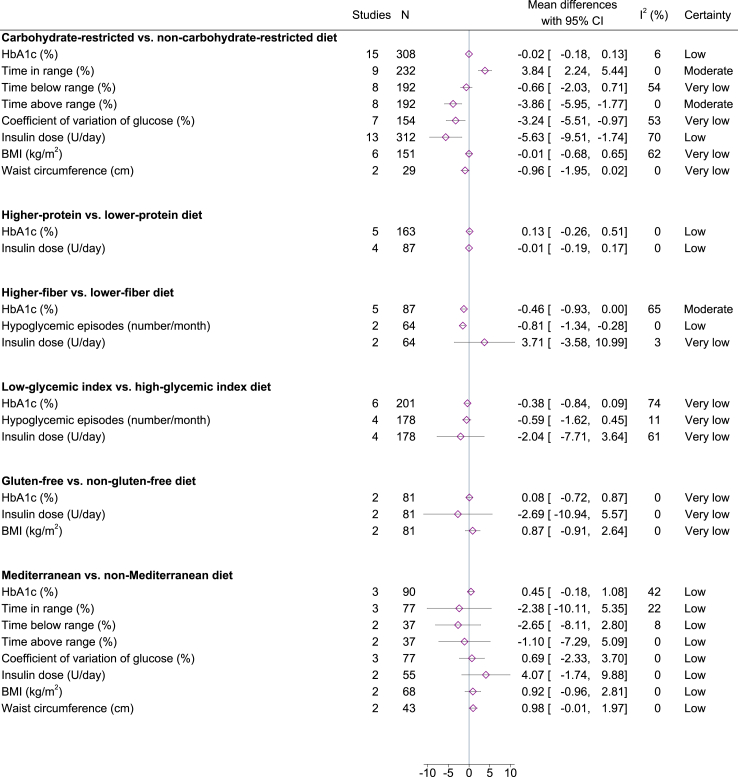
Summary mean differences with 95% confidence intervals for each dietary pattern comparison.

A total of 20 RCTs involving 448 participants were included in the analysis of carbohydrate-restricted diets. The difference in carbohydrate intakes between the intervention and control groups ranged from 7% to 41% of total energy. Carbohydrate-restricted diets did not affect HbA1c levels (Fig. 2). There was no evidence of publication bias (Egger’s test *P* = 0.9601), and the certainty of evidence for this null effect was low due to risk of bias and imprecision. Conversely, carbohydrate-restricted diets led to an increased TIR (MD 3.84%, 95% CI 2.24–5.44, I^2^ 0%) and a reduced TAR (MD −3.86%, 95% CI −5.95 to −1.77, I^2^ 0%), both with a moderate certainty of evidence. Additionally, individuals following a carbohydrate-restricted diet had a lower CV (MD −3.24%, 95% CI −5.51 to −0.97, I^2^ 53%) and required less insulin (MD −5.63 U/day, 95% CI −9.51 to −1.74, I^2^ 70%, Egger’s test *P* = 0.1510). The certainty of evidence for these effects was low due to risk of bias, inconsistency, and imprecision. Subgroup analyses indicated that effects on CV were more pronounced in studies with “some concerns” compared to those with a “low risk” of bias, while factors such as carbohydrate amount or substitute, intervention duration, or age did not play a role (Supplementary Figs. S39 and S40). In contrast, reductions in insulin doses were more pronounced in studies with lower carbohydrate intake or larger differences in carbohydrate intake between intervention and control groups (Supplementary Figs. S41 and S42). There was no effect of carbohydrate-restricted diets on TBR, BMI, or WC, although the certainty of evidence was very low. Some heterogeneity was observed in the meta-analyses of TBR (I^2^ 54%) and BMI (I^2^ 62%). Subgroup and meta-regression analyses indicated reductions in TBR among studies with “some concerns”, but not “low risk” of bias, and when differences in carbohydrate intake between intervention and control groups were larger (Supplementary Figs. S43 and S44). Subgroup and meta-regression analyses for BMI indicated that carbohydrate-restricted diets reduced BMI if their carbohydrate content was less than 26% of energy, but increased BMI if their carbohydrate content was 26–45% (Supplementary Figs. S45 and S46).

### Higher-protein versus lower-protein diet

The analysis of higher-protein diets included six RCTs with 191 participants. There was no effect of higher-protein diets on HbA1c or insulin dose (Fig. 2). The certainty of evidence for these null effects was low due to serious risk of bias and imprecision. Regarding other outcomes, a single RCT of female adolescents found no effects of a higher-protein diet on TIR, TAR, or TBR.

### Higher-fiber versus lower-fiber diet

A total of five RCTs, involving 87 participants, were analyzed for higher-fiber diets. There was evidence supporting the effectiveness of higher-fiber diets in reducing HbA1c levels (MD −0.46%, 95% CI −0.93 to 0.00, I^2^ 65%; Fig. 2). There was some heterogeneity between studies, likely explained by differences in intervention duration, participants’ age, and carbohydrate content (Supplementary Fig. S47). Specifically, higher-fiber diets appeared to reduce HbA1c when the intervention lasted longer (MD for 6–12 weeks −0.91%, 95% CI −1.24 to −0.59), in a single study of adolescents (MD −0.94%, 95% CI −1.27 to −0.61), and when the total carbohydrate content was the same between intervention and control groups (MD −0.69%, 95% CI −1.23 to −0.15). Nevertheless, all studies pointed towards benefit, and the certainty of evidence for this effect was rated as moderate. Additionally, higher-fiber diets led to fewer hypoglycemic episodes per month (MD −0.81, 95% CI −1.34 to −0.28, I^2^ 0%) with low certainty of evidence. The fiber content of the diet did not affect insulin dose, although certainty of evidence was very low. There was no information on other outcomes.

### Low-glycemic index versus high-glycemic index diet

Six RCTs, including 201 participants, were analyzed for low-glycemic index diets. There was no clear benefit of low-glycemic index diets on HbA1c (Fig. 2). Certainty of evidence was very low, primarily due to risk of bias and heterogeneity among the included studies. This heterogeneity was likely attributable to variations in the duration of the interventions and the age of the participants (Supplementary Fig S48). Notably, low-glycemic index diets appeared to reduce HbA1c levels when the intervention duration exceeded 12 weeks (MD −0.45%, 95% CI −0.76 to −0.14) and among child/adolescent participants (MD −0.83%, 95% CI −1.14 to −0.52). No effect was observed on the number of hypoglycemic episodes or insulin dose, although certainty of evidence was very low. No other outcomes were available for this comparison.

### Gluten-free versus non-gluten-free diet

Two RCTs, including 81 participants, were analyzed for gluten-free diets. Gluten-free diets had no effect on HbA1c, insulin dose, or BMI, although certainty of evidence was very low due to very serious risk of bias and imprecision (Fig. 2). The studies reported additional data on hypoglycemia and waist circumference, respectively, and found no effects.

### Mediterranean versus non-Mediterranean diet

A total of four RCTs involving 105 participants were included in the analysis of the Mediterranean diet. There was no effect of the Mediterranean diet on HbA1c, TIR, TBR, TAR, insulin dose, BMI, or waist circumference (Fig. 2). The certainty of evidence for these null effects was rated as low, mainly due to the imprecision of the estimates.

### Other diets

Other dietary patterns identified but not included in meta-analyses are intermittent fasting and the vegan diet. Based on a single RCT with 10 participants, intermittent fasting had no effect on HbA1c, insulin dose, or the number of hypoglycemic episodes compared to a restricted calorie diet.^49^ Similarly, a single RCT with 35 participants on vegan diet found no difference in HbA1c, CV, or TIR compared to a portion-controlled diet with no restriction on animal products.^50^ However, the vegan diet did lead to reductions in BMI (MD −1.6 kg/m^2^, 95% CI −2.2 to −0.9) and insulin dose (MD −10.7 U/day, 95% CI −21.3 to −0.2).^50^

## Discussion

This systematic review and meta-analysis of 35 RCTs aimed to synthesize the entire literature on effects of dietary patterns on the glucose management of type 1 diabetes. The most compelling evidence suggested that higher-fiber diets likely reduce HbA1c levels and may also help prevent hypoglycemia. Carbohydrate-restricted diets improved TIR, TAR, CV, and insulin requirements, although not HbA1c levels, with low to moderate certainty of evidence. The remaining dietary patterns, including higher-protein, low-glycemic index, gluten-free, Mediterranean, intermittent fasting, and vegan diets, did not exhibit effects on glucose management. However, the evidence for these patterns was generally weak, primarily due to the limited number of studies.

Our finding that higher-fiber diets likely reduce HbA1c levels in type 1 diabetes is consistent with previous meta-analyses of observational studies and RCTs that included mixed type 1 and type 2 diabetes populations.^9^^,^^52^ Additionally, we found that higher-fiber diets may help prevent hypoglycemia. The underlying mechanisms likely involve fiber's ability to slow digestion and the absorption of glucose.^53^ This process improves satiety, reduces postprandial hyperglycemia, and allows for a better match with insulin administration, thereby improving overall glucose control.^54^ Low-glycemic index diets may have similar effects on glucose management, as they also slow down glucose absorption.^55^ This is partly due to their high fiber content, but also because they include other complex carbohydrates, namely starches. While there was no overall benefit of low-glycemic index diets, we found a reduction in HbA1c if the diet was maintained for at least 12 weeks, which is often required to see changes in HbA1c, and in studies of children and adolescents. This reduction may be attributed to the higher metabolic rates and better insulin sensitivity in younger individuals. This finding aligns with a previous meta-analysis that included long-duration interventions and mainly children/adolescents,^56^ as well as meta-analyses involving individuals with type 2 diabetes.^56^^,^^57^

Our findings provide the strongest evidence to date supporting the effectiveness of carbohydrate-restricted diets in managing type 1 diabetes, based on an analysis of 20 RCTs, compared to the 9 included in the most recent meta-analysis on the topic.^10^ The observed improvements in TIR and CV align with the latest meta-analysis.^10^ Additionally, we could extend previous knowledge by identifying potential benefits in reducing insulin requirements. A possible explanation is that higher carbohydrate intake increases the likelihood of errors in carbohydrate counting, leading to incorrect insulin dosing and subsequent blood glucose fluctuations.^58^ This might also explain the lack of differences in HbA1c levels. Frequent highs and lows due to a higher carbohydrate diet may still result in stable HbA1c, as it reflects average glucose levels over the previous three months. Another potential reason for the lack of effect on HbA1c levels is that most interventions lasted less than three months, which may be insufficient to detect any changes in HbA1c. In contrast, carbohydrate-restricted diets appear to reduce HbA1c levels in type 2 diabetes.^59^ This discrepancy may be due to individuals with type 2 diabetes not experiencing substantial glucose fluctuations following a high-carbohydrate diet, but rather hyperglycemia, which subsequently increases HbA1c levels. Beneficial effects of carbohydrate-restricted diets on TIR, TAR, and CV are particularly noteworthy, since these metrics are linked to the risk of diabetes complications, independent of HbA1c.^60^ It is important to note that no study addressed the effects of very low-carbohydrate ketogenic diets (<10% total energy or <50 g/day), which are generally not recommended due to safety concerns.^5^^,^^6^

We found no evidence that the Mediterranean diet improves glycemic control in individuals with type 1 diabetes, which contrasts findings in type 2 diabetes.^59^ Components of the Mediterranean diet that may benefit glucose control in individuals with type 2 diabetes include its high content in unsaturated fats and antioxidants, which are linked to improved insulin sensitivity.^61^^,^^62^ Some benefits might also be expected in type 1 diabetes, given the Mediterranean diet's moderate carbohydrate content and high fiber. Notably, the evidence regarding the Mediterranean diet's effects on type 1 diabetes was of low certainty, and further research is needed to better understand its impact on type 1 diabetes management. Additionally, we found that abstaining from gluten, a protein found in wheat, did not influence HbA1c levels, which aligns with findings from a systematic review of observational studies.^63^ This diet is essential to manage celiac disease, which often co-occurs with type 1 diabetes, but may not provide additional benefits for glycemic control. On the contrary, it has raised concerns regarding its value in non-celiac populations as it is generally low in fiber and certain micronutrients.^64^

This is the first systematic review and meta-analysis to evaluate the effects of all dietary patterns studied in RCTs on glucose management in type 1 diabetes. Our analysis covered multiple aspects of glucose control, such as HbA1c, glucose variability, and hypoglycemia. Importantly, we focused exclusively on individuals with type 1 diabetes, avoiding the inclusion of those with type 2 diabetes, for whom dietary effects may differ. Additional strengths include the use of a predefined protocol, a thorough literature search conducted by a librarian, and the use of recommended tools for assessing bias and evidence certainty.

Our study has also some limitations. Restricting the searches to English publications and human studies may have led to omitting some relevant studies. To assess the impact of this restriction, we performed a search limited to non-English articles (n = 329), but did not identify any eligible studies. For most comparisons, there were fewer than 10 RCTs, which resulted in some imprecise estimates and limited opportunities for assessment of publication bias. Additionally, the definitions of dietary patterns varied between studies, making the interventions not fully comparable. For instance, some studies defined carbohydrate-restricted diets as containing 45% of total energy from carbohydrates, while others set the threshold at below 26%. Nevertheless, the impact of varying carbohydrate contents was assessed when heterogeneity was present. In addition, follow-up periods varied and were less than 12 weeks in several studies, which may be insufficient to observe changes in HbA1c. Conversely, shorter interventions might have been more reliable for outcomes such as TIR and CV, as adherence to the intervention may decline over longer periods. Furthermore, nine RCTs compared non-isocaloric diets, which may have been problematic in isolating intervention effects. This limitation, along with the lack of adherence noted in nine studies, has been addressed in the risk of bias assessment and was a primary reason for downgrading the certainty of evidence. It was not possible to investigate sex- and gender-specific effects of the different diets, as such estimates were not provided in the original studies. Lastly, our study focused on glucose control outcomes, but some studies also assessed effects on lipids, blood pressure, and kidney function, which are also important for type 1 diabetes management.

Our findings underscore the importance of both the quality and quantity of carbohydrates for glycemic control in children and adults with type 1 diabetes. This reinforces current diabetes management guidelines, which recommend a daily fiber intake of at least 35 g.^5^^,^^6^ Notably, the favorable effects of higher-fiber diets on HbA1c were less pronounced in studies with higher total carbohydrate content. This suggests that a higher intake of other carbohydrates, such as refined carbohydrates or sugars, may counteract the beneficial effects of fiber. This emphasizes the potential benefits of carbohydrate restriction. With appropriate nutritional counseling, it is feasible to meet fiber recommendations even on a very low-carbohydrate diet (<130 g) by incorporating seeds, nuts, beans, and whole fruits and vegetables.^65^ Including legumes and moderate amounts of whole grain cereals can further support fiber intake in less restrictive low-carbohydrate diets of up to 45% of total energy intake. To offset carbohydrate restriction, increasing fat intake is a common strategy. Although our observations did not indicate differences between various types of fat, it is advisable to limit saturated fats due to their adverse effects on cardiometabolic markers.

Despite these findings, current dietary guidelines have not endorsed carbohydrate restriction for people with type 1 diabetes, primarily due to concerns about long-term effects and safety. Future studies should investigate adverse events like severe hypoglycemia and ketoacidosis and extend intervention durations beyond 12 weeks. For children with diabetes, it is important to consider the potential adverse effects of carbohydrate restriction on growth. Additionally, future studies should include participants using the latest automated insulin pumps, investigate sex- and gender-specific effects, and extend to populations outside Europe and North America and pregnant women. Moreover, there is a scarcity of studies examining the effects of other common dietary patterns, such as the Mediterranean, Dietary Approaches to Stop Hypertension (DASH), vegetarian, or vegan diets, as well as studies on energy restriction in individuals with type 1 diabetes. Future reviews should also investigate the cardiometabolic effects of various dietary patterns in individuals with type 1 diabetes.

Higher-fiber diets emerged as potentially beneficial for glycemic control in individuals with type 1 diabetes. Maintaining a high-fiber diet while restricting other carbohydrate sources (e.g., refined carbohydrates or high-sugar foods) may offer additional advantages, though further research is needed to assess long-term effects and safety. Other dietary patterns showed no clear impact on glucose management, however further research is needed due to the weak evidence.

## Contributors

AML and JZ conceptualized the study; JZ and MB independently performed the study selection, data extraction, and risk of bias assessment, and consulted AML in case of disagreement; JZ and AML performed the statistical analyses, certainty of evidence assessment, visualization, and wrote the original draft; SC, JEL, AB, and MB reviewed and edited the manuscript; and all authors contributed to the interpretation of the findings. JZ, MB, and AML have directly accessed and verified the underlying data reported in the manuscript. All authors read and approved the final version of the manuscript and were responsible for the decision to submit the manuscript.

## Data sharing statement

All data used in this meta-analysis are publicly available from the original studies cited within this manuscript.

## Declaration of interests

AB has a consulting agreement with Abbott Scandinavia AB for the development of a digital program focusing on diet in people with type 2 diabetes, starting on March 26, 2025. This agreement does not pertain to the current manuscript.
