# Supplementary material for: Effects of different dietary patterns on glucose management in type 1 diabetes: a systematic review and meta-analysis of randomized controlled trials
Source: eClinicalMedicine. 2025 Apr 28;83:103222. doi: 10.1016/j.eclinm.2025.103222 (PMC12242849; doi:10.1016/j.eclinm.2025.103222)
Supplement: Supplementary Figs. S1–48 and Tables S1–S5 [file mmc1.pdf]

SUPPLEMENTARY MATERIAL

Effects of different dietary patterns on glucose management in type 1 diabetes: a systematic review and meta-analysis of randomized controlled trials

Authors:
Jiayi Zeng,[1,2] Miranda Beck,[1] Afroditi Barouti,[3,4] Josefin E. Löfvenborg,[5] Sofia Carlsson,[1] and Anna-Maria Lampousi[1,6]

[1]Institute of Environmental Medicine, Karolinska Institutet, Stockholm, Sweden
[2]Department of Epidemiological Methods and Etiological Research, Leibniz Institute for Prevention Research and Epidemiology - BIPS, Bremen, Germany
[3]Department of Molecular Medicine and Surgery, Karolinska Institutet, Stockholm, Sweden
[4]Center for Diabetes, Academic Specialist Center, Stockholm, Sweden
[5]Department of Risk and Benefit Assessment, Swedish Food Agency, Uppsala, Sweden
[6]Department of Medicine Solna, Clinical Epidemiology Division, Karolinska Institutet, Stockholm, Sweden

Correspondence to:
Anna-Maria Lampousi
Department of Medicine Solna, Clinical Epidemiology Division, T2, Karolinska Institutet
17176 Stockholm, Sweden
annamaria.lampousi@ki.se

Table of Contents

Supplementary Table 1. Search strategy for identification of relevant articles in Medline 5

Supplementary Table 2. Search strategy for identification of relevant articles in Embase 6

Supplementary Table 3. Search strategy for identification of relevant articles in Cochrane Library 7

Supplementary Table 4. Studies that were excluded after full text screening and reasons for exclusion 8

Supplementary Table 5. Certainty of evidence assessment using the GRADE system 9

Supplementary Figure 1. Risk of bias assessment of randomized controlled trials using RoB 2.0, generated with the robvis tool 12

Supplementary Figure 2. Summary of mean difference in HbA1c (%) for carbohydrate-restricted versus non-carbohydrate-restricted diets. 13

Supplementary Figure 3. Summary of mean difference in time in range (%) for carbohydrate-restricted versus non-carbohydrate-restricted diets. 14

Supplementary Figure 4. Summary of mean difference in time below range (%) for carbohydrate-restricted versus non-carbohydrate-restricted diets. 15

Supplementary Figure 5. Summary of mean difference in time above range (%) for carbohydrate-restricted versus non-carbohydrate-restricted diets. 16

Supplementary Figure 6. Summary of mean difference in coefficient of variation (%) for carbohydrate-restricted versus non-carbohydrate-restricted diets. 17

Supplementary Figure 7. Summary of mean difference in insulin dose (U/day) for carbohydrate-restricted versus non-carbohydrate-restricted diets. 18

Supplementary Figure 8. Summary of mean difference in body mass index (kg/m^2) for carbohydrate-restricted versus non-carbohydrate-restricted diets. 19

Supplementary Figure 9. Summary of mean difference in waist circumference (cm) for carbohydrate-restricted versus non-carbohydrate-restricted diets. 20

Supplementary Figure 10. Summary of mean difference in HbA1c (%) for higher-protein versus lower-protein diets. 21

Supplementary Figure 11. Summary of mean difference in insulin dose (U/day) for higher-protein versus lower-protein diets. 22

Supplementary Figure 12. Summary of mean difference in HbA1c (%) for higher-fiber versus lower-fiber diets. 23

Supplementary Figure 13. Summary of mean difference in number of hypoglycemic episodes per month for higher-fiber versus lower-fiber diets. 24

Supplementary Figure 14. Summary of mean difference in insulin dose (U/day) for higher-fiber versus lower-fiber diets diets. 25

Supplementary Figure 15. Summary of mean difference in HbA1c (%) for low-glycemic index versus high-glycemic index diets. 26

Supplementary Figure 16. Summary of mean difference in number of hypoglycemic episodes per month for low-glycemic index versus high-glycemic index diets. 27

Supplementary Figure 17. Summary of mean difference in insulin dose (U/day) for low-glycemic index versus high-glycemic index diets. 28

Supplementary Figure 18. Summary of mean difference in HbA1c (%) for gluten-free versus non-gluten-free diets. 29

Supplementary Figure 19. Summary of mean difference in insulin dose (U/day) for gluten-free versus non-gluten-free diets. 30

Supplementary Figure 20. Summary of mean difference in body mass index (kg/m^2) for gluten-free versus non-gluten-free diets. 31

Supplementary Figure 21. Summary of mean difference in HbA1c (%) for Mediterranean versus non-Mediterranean diets. 32

Supplementary Figure 22. Summary of mean difference in time in range (%) for Mediterranean versus non-Mediterranean diets. 33

Supplementary Figure 23. Summary of mean difference in time below range (%) for Mediterranean versus non-Mediterranean diets. 34

Supplementary Figure 24. Summary of mean difference in time above range (%) for Mediterranean versus non-Mediterranean diets. 35

Supplementary Figure 25. Summary of mean difference in coefficient of variation (%) for Mediterranean versus non-Mediterranean diets. 36

Supplementary Figure 26. Summary of mean difference in insulin dose (U/day) for Mediterranean versus non-Mediterranean diets. 37

Supplementary Figure 27. Summary of mean difference in body mass index (kg/m^2) for Mediterranean versus non-Mediterranean diets. 38

Supplementary Figure 28. Summary of mean difference in waist circumference (cm) for Mediterranean versus non-Mediterranean diets. 39

Supplementary Figure 29. Contour-enhanced funnel plot of studies on carbohydrate-restricted versus non-carbohydrate-restricted diets and HbA1c change. 40

Supplementary Figure 30. Contour-enhanced funnel plot of studies on carbohydrate-restricted versus non-carbohydrate-restricted diets and insulin change. 41

Supplementary Figure 31. Contour-enhanced funnel plot of studies on carbohydrate-restricted versus non-carbohydrate-restricted diets and time in range change. 42

Supplementary Figure 32. Contour-enhanced funnel plot of studies on carbohydrate-restricted versus non-carbohydrate-restricted diets and time above range change. 43

Supplementary Figure 33. Contour-enhanced funnel plot of studies on carbohydrate-restricted versus non-carbohydrate-restricted diets and time below range change. 44

Supplementary Figure 34. Contour-enhanced funnel plot of studies on carbohydrate-restricted versus non-carbohydrate-restricted diets and coefficient of variation change. 45

Supplementary Figure 35. Contour-enhanced funnel plot of studies on carbohydrate-restricted versus non-carbohydrate-restricted diets and BMI change. 46

Supplementary Figure 36. Contour-enhanced funnel plot of studies on higher-protein versus lower-protein diets and HbA1c change. 47

Supplementary Figure 37. Contour-enhanced funnel plot of studies on higher-fiber versus lower-fiber diets and HbA1c change. 48

Supplementary Figure 38. Contour-enhanced funnel plot of studies on higher-glycemic index versus lower-glycemic index diets and HbA1c change. 49

Supplementary Figure 39. Subgroup analyses for summary of mean difference in coefficient of variation (%) for carbohydrate-restricted versus non-carbohydrate-restricted diets. 50

Supplementary Figure 40. Meta-regression of mean difference in coefficient of variation (%) for carbohydrate-restricted versus non-carbohydrate-restricted diets with (A) carbohydrate % of total energy intake ($p=0.539$) and (B) carbohydrate difference between intervention and control group ($p=0.058$) as effect modifiers. 51

Supplementary Figure 41. Subgroup analyses for summary of mean difference in insulin dose (U/day) for carbohydrate-restricted versus non-carbohydrate-restricted diets. 52

Supplementary Figure 42. Meta-regression of mean difference in insulin dose (U/day) for carbohydrate-restricted versus non-carbohydrate-restricted diets with (A) carbohydrate % of total energy intake ($p<0.001$) and (B) carbohydrate difference between intervention and control group ($p=0.003$) as effect modifiers. 53

Supplementary Figure 43. Subgroup analyses for summary of mean difference in time below range (%) for carbohydrate-restricted versus non-carbohydrate-restricted diets. 54

Supplementary Figure 44. Meta-regression of mean difference in time below range (%) for carbohydrate-restricted versus non-carbohydrate-restricted diets with (A) carbohydrate % of total energy intake ($p=0.113$) and (B) carbohydrate difference between intervention and control group ($p=0.001$) as effect modifiers. 55

Supplementary Figure 45. Subgroup analyses for summary of mean difference in body mass index (kg/m^2) for carbohydrate-restricted versus non-carbohydrate-restricted diets. 56

Supplementary Figure 46. Meta-regression of mean difference in body mass index (kg/m^2) for carbohydrate-restricted versus non-carbohydrate-restricted diets with (A) carbohydrate % of total energy intake ($p<0.001$) and (B) carbohydrate difference between intervention and control group ($p=0.032$) as effect modifiers. 57

Supplementary Figure 47. Subgroup analyses for summary of mean difference in HbA1c (%) for higher-fiber versus lower-fiber diets. 58

Supplementary Figure 48. Subgroup analyses for summary of mean difference in HbA1c (%) for low-glycemic index versus high-glycemic index diets. 59

Supplementary references 60

Supplementary Table 1. Search strategy for identification of relevant articles in Medline

Interface: Ovid MEDLINE(R) ALL<br><br>Date of Search: 22 December 2023<br><br>Number of hits: 1,868<br><br>Comment: In Ovid, two or more words are automatically searched as phrases; i.e. no quotation marks are needed	Field labels<br><br>• exp/ = exploded MeSH term<br>• / = non exploded MeSH term<br>• .ti,ab,kf. = title, abstract and author keywords<br>• adjx = within x words, regardless of order<br>• * = truncation of word for alternate endings

#	Searches	Results
1	Diabetes, type-1/	86984
2	(diabetes adj3 (autoimmun* or brittle or juvenile or ketosis-prone or sudden-onset or type 1)).ti,ab,kf.	61527
3	((insulin dependent not non insulin dependent) and diabetes).ti,ab,kf.	15209
4	(iddm or t1d or t1dm).ti,ab,kf.	24145
5	or/1-4	111721
6	exp Diet/	333532
7	exp Dietary carbohydrates/	102160
8	exp Dietary fats/	98921
9	exp Dietary fiber/	24094
10	exp Dietary Proteins/	107646
11	exp Nutrients/	601885
12	exp Nutritive value/	19739
13	(caloric restriction or carbohydrate* or dash or diet* or fiber? or fibre? or fat? or fodmap or gluten or glyc?emic index or glyc?emic load or ketogenic or low calori? or high calori? or macrobiotic or macronutrient* or mediterranean or monounsaturated or nutrient? or nutritive* or paleolithic or polyunsaturated or protein? or raw food or saturated or vegan or vegetarian).ti,ab,kf.	4980284
14	((intermittent or periodic* or time-restrict*) adj3 (eating or fasting)).ti,ab,kf.	1924
15	or/6-14	5602467
16	controlled clinical trial.pt.	95490
17	randomized controlled trial.pt.	605257
18	Randomized Controlled Trials as Topic/	165755
19	placebo.ti,ab.	250792
20	(randomly or randomi#ation or randomi#ed).ti,ab,kf.	1160576
21	trial.ti,ab.	784736
22	((double or single or doubly or singly) adj (blind or blinded or blindly)).ti,ab.	195343
23	or/16-22	1809853
24	5 and 15 and 23	2097
25	(animals not humans).sh.	5145487
26	24 not 25	1940
27	limit 26 to english language	1868

Supplementary Table 2. Search strategy for identification of relevant articles in Embase

Interface: embase.com<br><br>Date of Search: 21 December 2023<br><br>Number of hits: 3,719<br><br>Comment: Emtree is the controlled vocabulary in Embase	Field labels<br>• /exp = exploded Emtree term<br>• /de = non exploded Emtree term<br>• ti,ab,kw = title, abstract and author keywords<br>• NEAR/x = within x words, regardless of order<br>• * = truncation of word for alternate endings

No.	Query	Results
#1	'insulin dependent diabetes mellitus'/de	148797
#2	(diabetes NEAR/3 (autoimmun* OR brittle OR juvenile OR 'ketosis prone' OR 'sudden onset' OR 'type 1')):ti,ab,kw	98487
#3	iddm:ti,ab,kw OR t1d:ti,ab,kw OR t1dm:ti,ab,kw OR ('insulin dependent':ti,ab,kw NOT 'non insulin dependent':ti,ab,kw AND diabetes:ti,ab,kw)	57246
#4	#1 OR #2 OR #3	185276
#5	'diet'/exp	437928
#6	'dietary intake'/de	98410
#7	'carbohydrate intake'/exp	35562
#8	'fat intake'/exp	59005
#9	'fiber intake'/de	2006
#10	'protein intake'/de	49877
#11	'glycemic index'/de	8400
#12	'glycemic load'/de	1997
#13	'nutrient intake'/de	3402
#14	'nutritional parameters'/de	5576
#15	'caloric restriction':ti,ab,kw OR carbohydrate*:ti,ab,kw OR dash:ti,ab,kw OR diet*:ti,ab,kw OR fiber$:ti,ab,kw OR fibre$:ti,ab,kw OR fat$:ti,ab,kw OR fodmap:ti,ab,kw OR gluten:ti,ab,kw OR 'glyc$emic index':ti,ab,kw OR 'glyc$emic load':ti,ab,kw OR ketogenic:ti,ab,kw OR 'low calori$':ti,ab,kw OR 'high calori$':ti,ab,kw OR macrobiotic:ti,ab,kw OR macronutrient*:ti,ab,kw OR mediterranean:ti,ab,kw OR monounsaturated:ti,ab,kw OR nutrient$:ti,ab,kw OR nutritive*:ti,ab,kw OR paleolithic:ti,ab,kw OR polyunsaturated:ti,ab,kw OR protein$:ti,ab,kw OR 'raw food':ti,ab,kw OR saturated:ti,ab,kw OR vegan:ti,ab,kw OR vegetarian:ti,ab,kw	6016330
#16	((intermittent OR periodic* OR 'time restrict*') NEAR/3 (eating OR fasting)):ti,ab,kw	2489
#17	#5 OR #6 OR #7 OR #8 OR #9 OR #10 OR #11 OR #12 OR #13 OR #14 OR #15 OR #16	6139443
#18	('randomized controlled trial'/de OR 'controlled clinical trial'/de OR random*:ti,ab,tt OR 'randomization'/de OR 'intermethod comparison'/de OR placebo:ti,ab,tt OR compare:ti,tt OR compared:ti,tt OR comparison:ti,tt OR ((evaluated:ab OR evaluate:ab OR evaluating:ab OR assessed:ab OR assess:ab) AND (compare:ab OR compared:ab OR comparing:ab OR comparison:ab)) OR ((open NEXT/1 label):ti,ab,tt) OR (((double OR single OR doubly OR singly) NEXT/1 (blind OR blinded OR blindly)):ti,ab,tt) OR 'double blind procedure'/de OR ((parallel NEXT/1 group*):ti,ab,tt) OR crossover:ti,ab,tt OR 'cross over':ti,ab,tt OR (((assign* OR match OR matched OR allocation) NEAR/6 (alternate OR group OR groups OR intervention OR interventions OR patient OR patients OR subject OR subjects OR participant OR participants)):ti,ab,tt) OR assigned:ti,ab,tt OR allocated:ti,ab,tt OR ((controlled NEAR/8 (study OR design OR trial)):ti,ab,tt) OR volunteer:ti,ab,tt OR volunteers:ti,ab,tt OR 'human experiment'/de OR trial:ti,tt) NOT (((random* NEXT/1 sampl* NEAR/8 ('cross section*' OR questionnaire* OR survey OR surveys OR database OR databases)):ti,ab,tt) NOT ('comparative study'/de OR 'controlled study'/de OR 'randomised controlled':ti,ab,tt OR 'randomized controlled':ti,ab,tt OR 'randomly assigned':ti,ab,tt) OR ('cross-sectional study' NOT ('randomized controlled trial'/de OR 'controlled clinical study'/de OR 'controlled study'/de OR 'randomised controlled':ti,ab,tt OR 'randomized controlled':ti,ab,tt OR 'control group':ti,ab,tt OR 'control groups':ti,ab,tt)) OR ('case control*':ti,ab,tt AND random*:ti,ab,tt NOT ('randomised controlled':ti,ab,tt OR 'randomized controlled':ti,ab,tt)) OR ('systematic review':ti,tt NOT (trial:ti,tt OR study:ti,tt)) OR (nonrandom*:ti,ab,tt NOT random*:ti,ab,tt) OR 'random field*':ti,ab,tt OR (('random cluster' NEAR/4 sampl*):ti,ab,tt) OR (review:ab AND review:it NOT trial:ti,tt) OR ('we searched':ab AND (review:ti,tt OR review:it)) OR 'update review':ab OR ((databases NEAR/5 searched):ab) OR ((rat:ti,tt OR rats:ti,tt OR mouse:ti,tt OR mice:ti,tt OR swine:ti,tt OR porcine:ti,tt OR murine:ti,tt OR sheep:ti,tt OR lambs:ti,tt OR pigs:ti,tt OR piglets:ti,tt OR rabbit:ti,tt OR rabbits:ti,tt OR cat:ti,tt OR cats:ti,tt OR dog:ti,tt OR dogs:ti,tt OR cattle:ti,tt OR bovine:ti,tt OR monkey:ti,tt OR monkeys:ti,tt OR trout:ti,tt OR marmoset*:ti,tt) AND 'animal experiment'/de) OR ('animal experiment'/de NOT ('human experiment'/de OR 'human'/de)))	5708602
#19	#4 AND #17 AND #18	6954
#20	#4 AND #17 AND #18 AND ([conference abstract]/lim OR [conference paper]/lim OR [conference review]/lim)	2297
#21	#19 NOT #20	3942
#22	#19 NOT #20 AND [english]/lim	3719

Supplementary Table 3. Search strategy for identification of relevant articles in Cochrane Library

Interface: Wiley<br><br>Date of Search: 21 December 2023<br><br>Number of hits: 1,679	Field labels<br>• ti,ab,kw = title, abstract and author keywords<br>• NEAR/x = within x words, regardless of order<br>• * = truncation of word for alternate endings

ID	Search	Hits
#1	[mh ^"Diabetes, type 1"]	6880
#2	(diabetes:ti,ab,kw NEAR/3 (autoimmun*:ti,ab,kw OR brittle:ti,ab,kw OR juvenile:ti,ab,kw OR ketosis-prone:ti,ab,kw OR sudden-onset:ti,ab,kw OR "type 1":ti,ab,kw))	11892
#3	(iddm:ti,ab,kw OR t1d:ti,ab,kw OR t1dm:ti,ab,kw)	2297
#4	(("insulin dependent" NOT "non insulin dependent") AND diabetes):ti,ab,kw	5362
#5	#1 OR #2 OR #3 OR #4	13140
#6	[mh Diet]	25912
#7	[mh "Dietary carbohydrates"]	7453
#8	[mh "Dietary fats"]	8828
#9	[mh "Dietary fiber"]	2552
#10	[mh "Dietary Proteins"]	4848
#11	[mh Nutrients]	6458
#12	[mh "Nutritive value"]	1674
#13	("caloric restriction":ti,ab,kw OR carbohydrate*:ti,ab,kw OR dash:ti,ab,kw OR diet*:ti,ab,kw OR fiber?:ti,ab,kw OR fibre?:ti,ab,kw OR fat?:ti,ab,kw OR fodmap:ti,ab,kw OR gluten:ti,ab,kw OR (glyc?emic NEXT "index"):ti,ab,kw OR (glyc?emic NEXT "load"):ti,ab,kw OR ketogenic:ti,ab,kw OR ("low" NEXT calori?):ti,ab,kw OR ("high" NEXT calori?):ti,ab,kw OR macrobiotic:ti,ab,kw OR macronutrient*:ti,ab,kw OR mediterranean:ti,ab,kw OR monounsaturated:ti,ab,kw OR nutrient?:ti,ab,kw OR nutritive*:ti,ab,kw OR paleolithic:ti,ab,kw OR polyunsaturated:ti,ab,kw OR protein?:ti,ab,kw OR "raw food":ti,ab,kw OR saturated:ti,ab,kw OR vegan:ti,ab,kw OR vegetarian:ti,ab,kw)	245511
#14	((intermittent:ti,ab,kw OR periodic*:ti,ab,kw OR time-restrict*:ti,ab,kw) NEAR/3 (eating:ti,ab,kw OR fasting:ti,ab,kw))	614
#15	#6 OR #7 OR #8 OR #9 OR #10 OR #11 OR #12 OR #13 OR #14	252813
#16	#5 AND #15 NOT (clinicaltrials or trialsearch):so	2351
#17	conference proceeding:pt	232480
#18	#16 NOT #17	1755
#19	Limit to english	1670

Supplementary Table 4. Studies that were excluded after full text screening and reasons for exclusion

Reason	Number of studies	References
Irrelevant study design	69	1-69
Irrelevant intervention	40	70-109
Irrelevant outcome	9	110-118
Conference paper	5	119-123
Wrong population	5	124-128
Insufficient data	2	129-130

Supplementary Table 5. Certainty of evidence assessment using the GRADE system

Certainty assessment								N	Outcome	Summary MD (95% CI)	Certainty
Studies	Study design	Risk of bias	Inconsistency	Indirectness	Imprecision	Publication bias	Other				
**Carbohydrate-restricted versus non-carbohydrate-restricted diet**											
15	RCT	Serious[1]	Not serious	Not serious	Serious[2]	Not serious		308	HbA1c (%)	-0.02 (-0.18, 0.13)	⊕⊕◯◯ LOW
9	RCT	Serious[1]	Not serious	Not serious	Not serious	Not serious		232	TIR (%)	3.84 (2.24, 5.44)	⊕⊕⊕◯ MODERATE
8	RCT	Serious[1]	Serious[3]	Not serious	Very serious[4]	Not serious		192	TBR (%)	-0.66 (-2.03, 0.71)	⊕◯◯◯ VERY LOW
8	RCT	Serious[1]	Not serious	Not serious	Not serious	Not serious		192	TAR (%)	-3.86 (-5.95, -1.77)	⊕⊕⊕◯ MODERATE
7	RCT	Serious[1]	Serious[3]	Not serious	Serious[5]	Not serious		154	CV (%)	-3.24 (-5.51, -0.97)	⊕◯◯◯ VERY LOW
13	RCT	Serious[1]	Serious[3]	Not serious	Serious[5]	Not serious	Dose-response	312	Insulin dose (U/day)	-5.63 (-9.51, -1.74)	⊕⊕◯◯ LOW
6	RCT	Serious[1]	Serious[3]	Not serious	Very serious[4]	Not serious	Dose-response	151	BMI (kg/m^2)	-0.01 (-0.68, 0.65)	⊕◯◯◯ VERY LOW
2	RCT	Serious[1]	Not serious	Not serious	Very serious[4]	-		29	WC (cm)	-0.96 (-1.95, 0.02)	⊕◯◯◯ VERY LOW
**Higher-protein versus lower-protein diet**											
5	RCT	Serious[1]	Not serious	Not serious	Serious[2]	Not serious		163	HbA1c (%)	0.13 (-0.26, 0.51)	⊕⊕◯◯ LOW
4	RCT	Serious[1]	Not serious	Not serious	Serious[2]	-		87	Insulin dose (U/day)	-0.01 (-0.19, 0.17)	⊕⊕◯◯ LOW
**Higher-fiber versus lower-fiber diet**											
5	RCT	Not serious	Not serious	Serious[6]	Serious[2]	Not serious	Large effect	87	HbA1c (%)	-0.46 (-0.93, 0.00)	⊕⊕⊕◯ MODERATE

Certainty assessment								N	Outcome	Summary MD (95% CI)	Certainty
Studies	Study design	Risk of bias	Inconsistency	Indirectness	Imprecision	Publication bias	Other				
2	RCT	Serious[1]	Not serious	Not serious	Serious[5]	-		64	Hypoglycemic episodes/month	-0.81 (-1.34, -0.28)	⊕⊕◯◯ LOW
2	RCT	Serious[1]	Not serious	Not serious	Very serious[4]	-		64	Insulin dose (U/day)	3.71 (-3.58, 10.99)	⊕◯◯◯ VERY LOW
**Low-glycemic index versus high-glycemic index diet**											
6	RCT	Serious[1]	Serious[3]	Serious[6]	Serious[2]	Serious[7]		201	HbA1c (%)	-0.38 (-0.84, 0.09)	⊕◯◯◯ VERY LOW
4	RCT	Serious[1]	Not serious	Not serious	Very serious[4]	-		178	Hypoglycemic episodes/month	-0.59 (-1.62, 0.45)	⊕◯◯◯ VERY LOW
4	RCT	Serious[1]	Serious[3]	Not serious	Very serious[4]	-		178	Insulin dose (U/day)	-2.04 (-7.71, 3.64)	⊕◯◯◯ VERY LOW
**Gluten-free versus non-gluten-free diet**											
2	RCT	Very serious[8]	Not serious	Not serious	Very serious[4]	-		81	HbA1c (%)	0.08 (-0.72, 0.87)	⊕◯◯◯ VERY LOW
2	RCT	Very serious[8]	Not serious	Not serious	Very serious[4]	-		81	Insulin dose (U/day)	-2.69 (-10.94, 5.57)	⊕◯◯◯ VERY LOW
2	RCT	Very serious[8]	Not serious	Not serious	Very serious[4]	-		81	BMI (kg/m^2)	0.87 (-0.91, 2.64)	⊕◯◯◯ VERY LOW
**Mediterranean versus non-Mediterranean diet**											
3	RCT	Not serious	Not serious	Not serious	Very serious[4]	-		90	HbA1c (%)	0.45 (-0.18, 1.08)	⊕⊕◯◯ LOW
3	RCT	Not serious	Not serious	Not serious	Very serious[4]	-		77	TIR (%)	-2.38 (-10.11, 5.35)	⊕⊕◯◯ LOW

Certainty assessment								N	Outcome	Summary MD (95% CI)	Certainty
Studies	Study design	Risk of bias	Inconsistency	Indirectness	Imprecision	Publication bias	Other				
2	RCT	Not serious	Not serious	Not serious	Very serious[4]	-		37	TBR (%)	-2.65 (-8.11, 2.80)	⊕⊕◯◯ LOW
2	RCT	Not serious	Not serious	Not serious	Very serious[4]	-		37	TAR (%)	-1.10 (-7.29, 5.09)	⊕⊕◯◯ LOW
3	RCT	Not serious	Not serious	Not serious	Very serious[4]	-		77	CV (%)	0.69 (-2.33, 3.70)	⊕⊕◯◯ LOW
2	RCT	Not serious	Not serious	Not serious	Very serious[4]	-		55	Insulin dose (U/day)	4.07 (-1.74, 9.88)	⊕⊕◯◯ LOW
2	RCT	Not serious	Not serious	Not serious	Very serious[4]	-		68	BMI (kg/m^2)	0.92 (-0.96, 2.81)	⊕⊕◯◯ LOW
2	RCT	Not serious	Not serious	Not serious	Very serious[4]	-		43	WC (cm)	0.98 (-0.01, 1.97)	⊕⊕◯◯ LOW

[1]None of the studies were blinded, and at least half were rated as having some concerns or high risk of bias, mainly due to poor adherence to the intervention or non-isocaloric diets. Therefore, we decided to downgrade by one level.
[2]The confidence interval includes the possibility of both harms (or no effects) and benefits, but it is not considerably wide. Therefore, we decided to downgrade by one level.
[3]There was moderate heterogeneity ($I^2 > 50\%$) and not all studies point towards benefit. Therefore, we decided to downgrade by one level.
[4]The confidence interval includes the possibility of both harms (or no effects) and benefits, and it is considerably wide. Therefore, we decided to downgrade by two levels.
[5]The confidence interval includes only the possibility of benefits, but the optimal information size is not reached. Therefore, we decided to downgrade by one level.
[6]Effects differed between children/adolescents and adults ($p<0.05$), but both point towards benefit. Therefore, we decided to downgrade by one level.
[7]Inspection of the funnel plot suggested that studies are missing from the areas of statistical non-significance.
[8]Both studies were rated as having some concerns or high risk of bias, mainly due to poor adherence to the intervention and non-isocaloric diets. Therefore, we decided to downgrade by two levels.

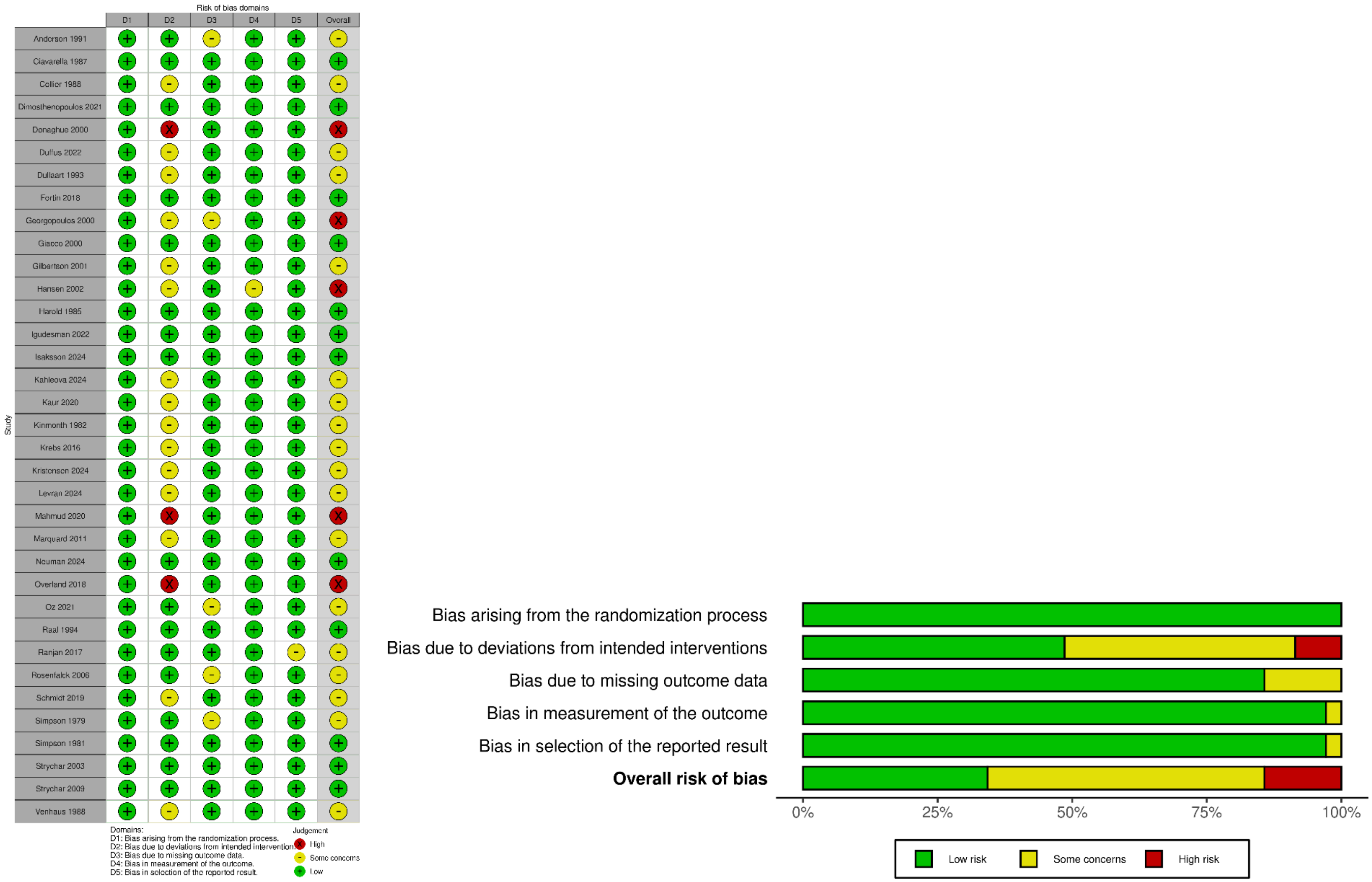

Supplementary Figure 1. Risk of bias assessment of randomized controlled trials using RoB 2.0, generated with the robvis tool.

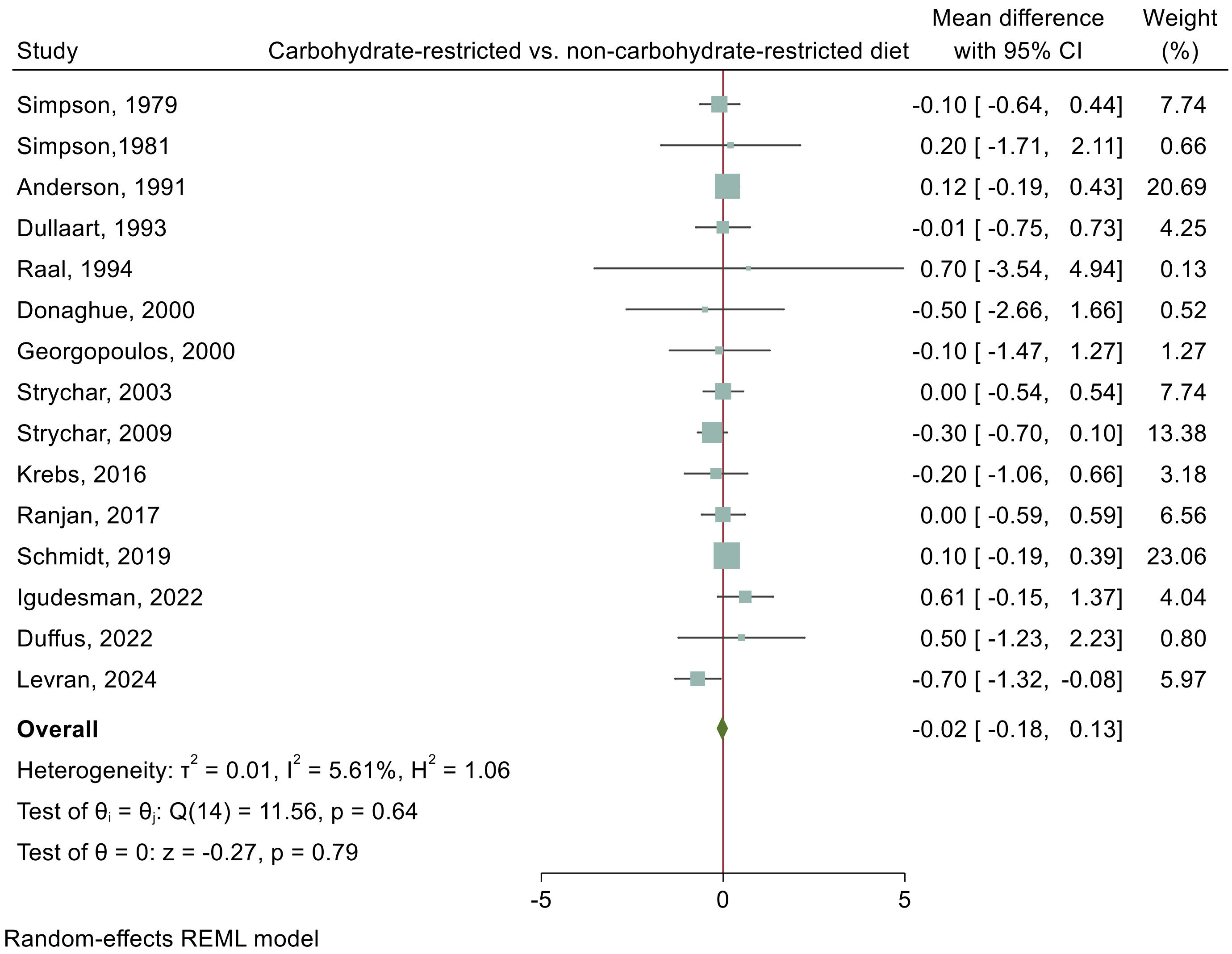

Supplementary Figure 2. Summary of mean difference in HbA1c (%) for carbohydrate-restricted versus non-carbohydrate-restricted diets.

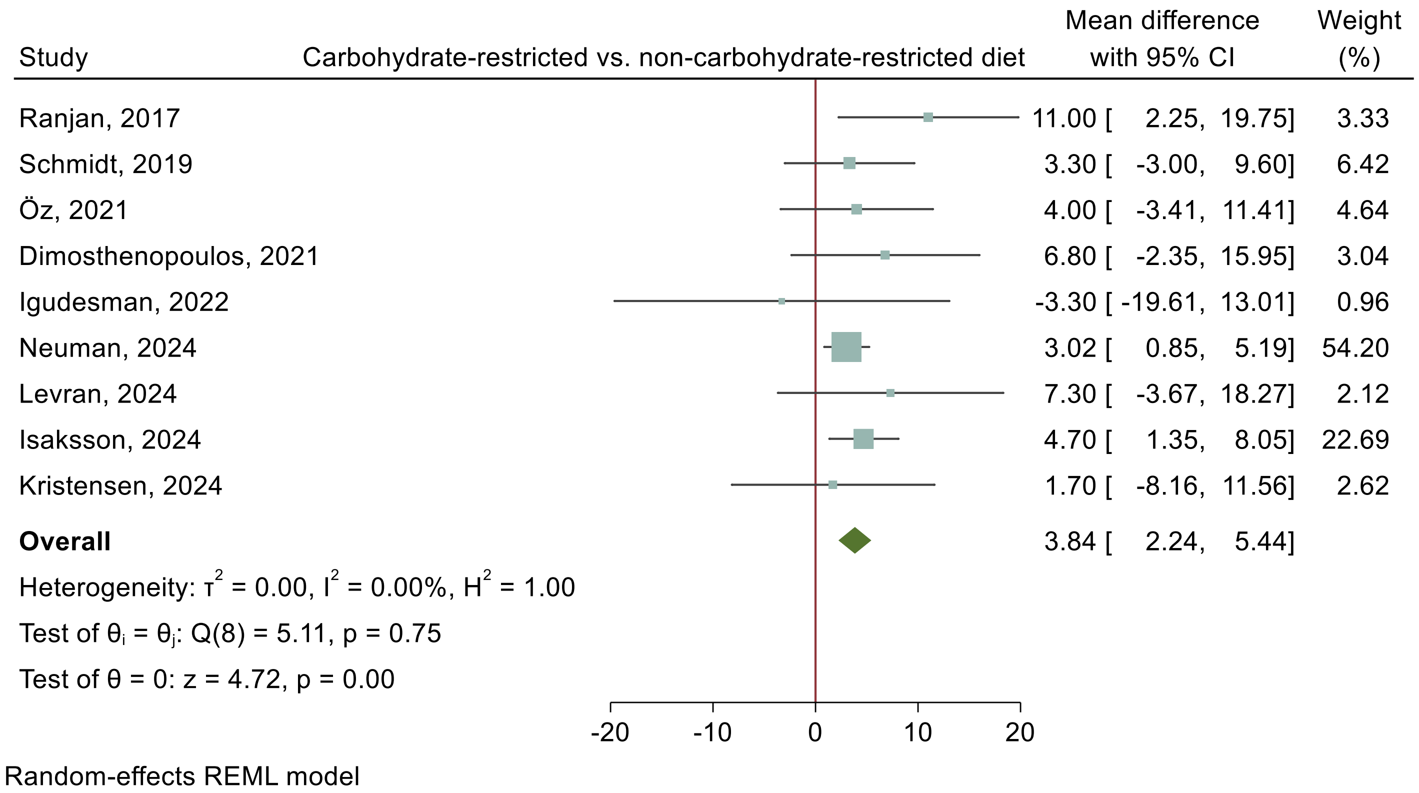

Supplementary Figure 3. Summary of mean difference in time in range (%) for carbohydrate-restricted versus non-carbohydrate-restricted diets.

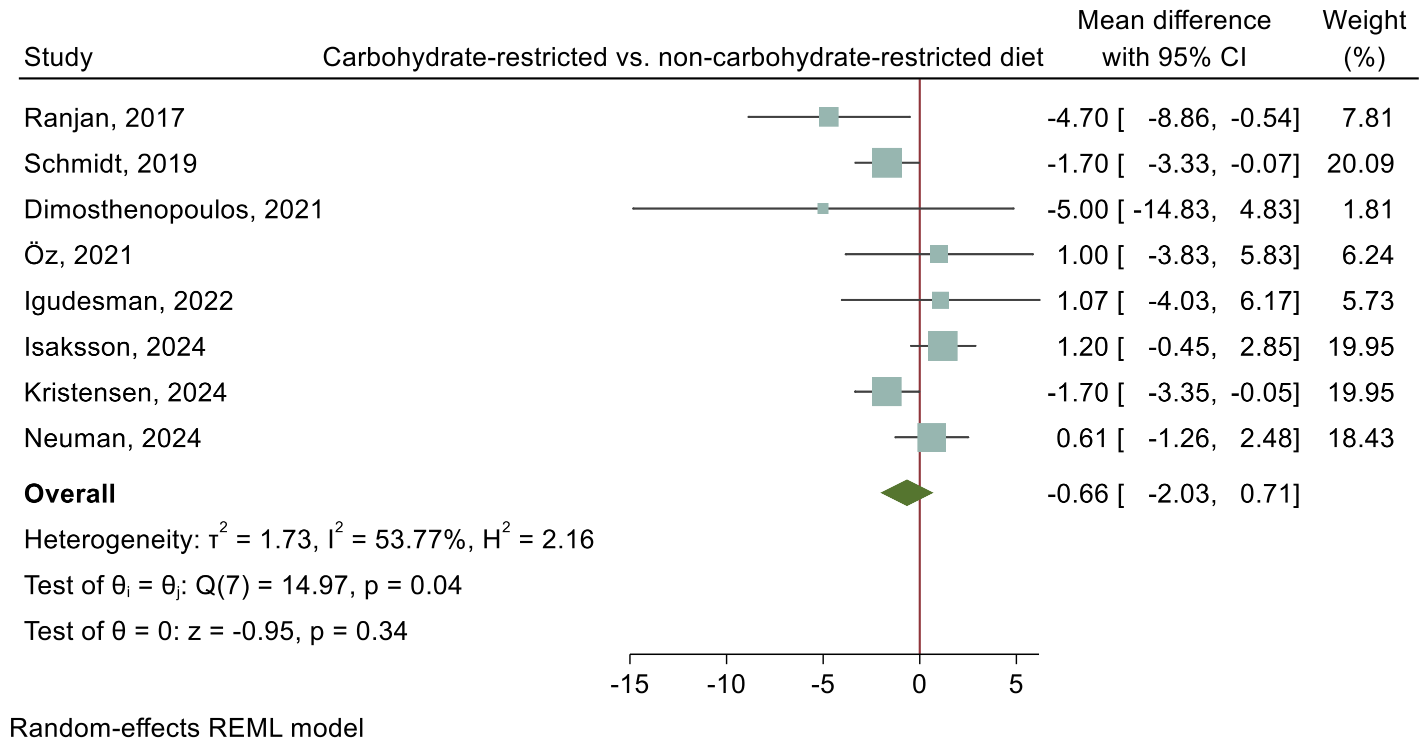

Supplementary Figure 4. Summary of mean difference in time below range (%) for carbohydrate-restricted versus non-carbohydrate-restricted diets.

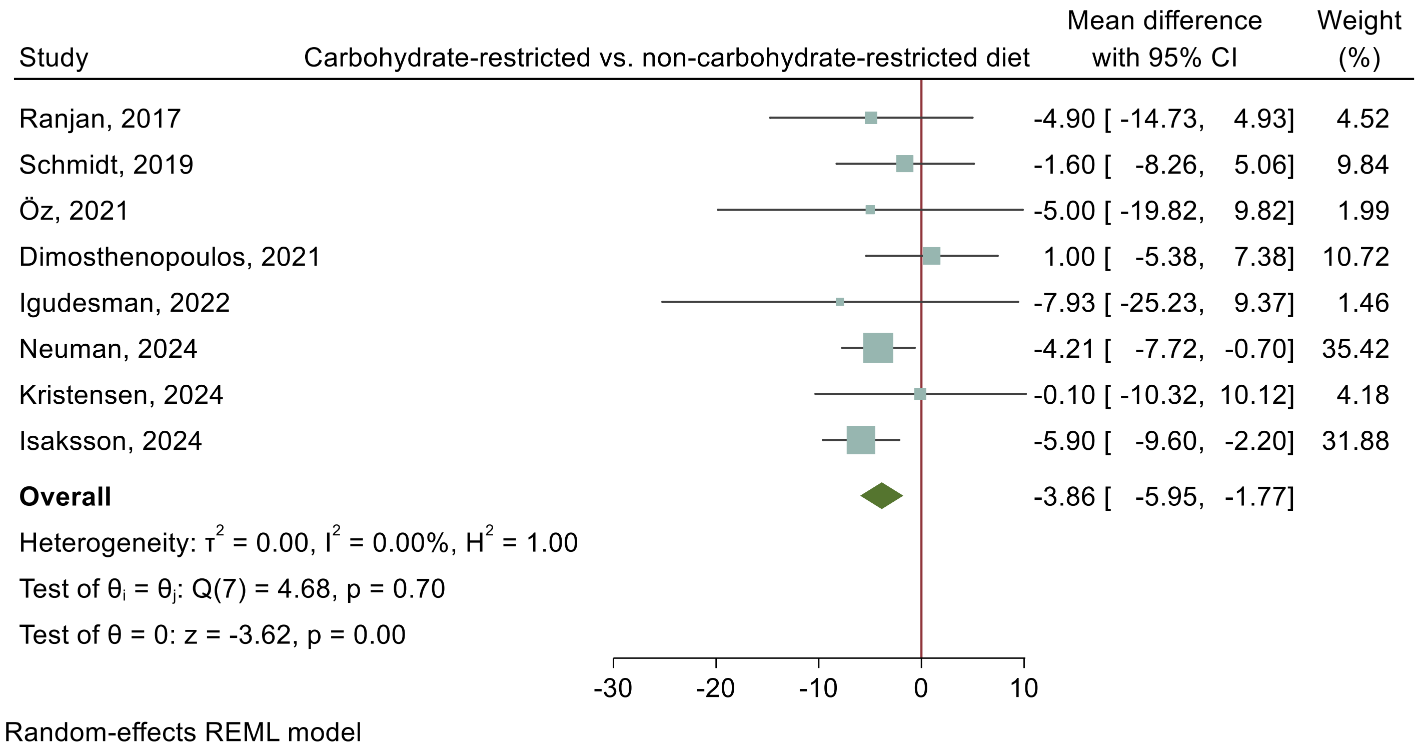

Supplementary Figure 5. Summary of mean difference in time above range (%) for carbohydrate-restricted versus non-carbohydrate-restricted diets.

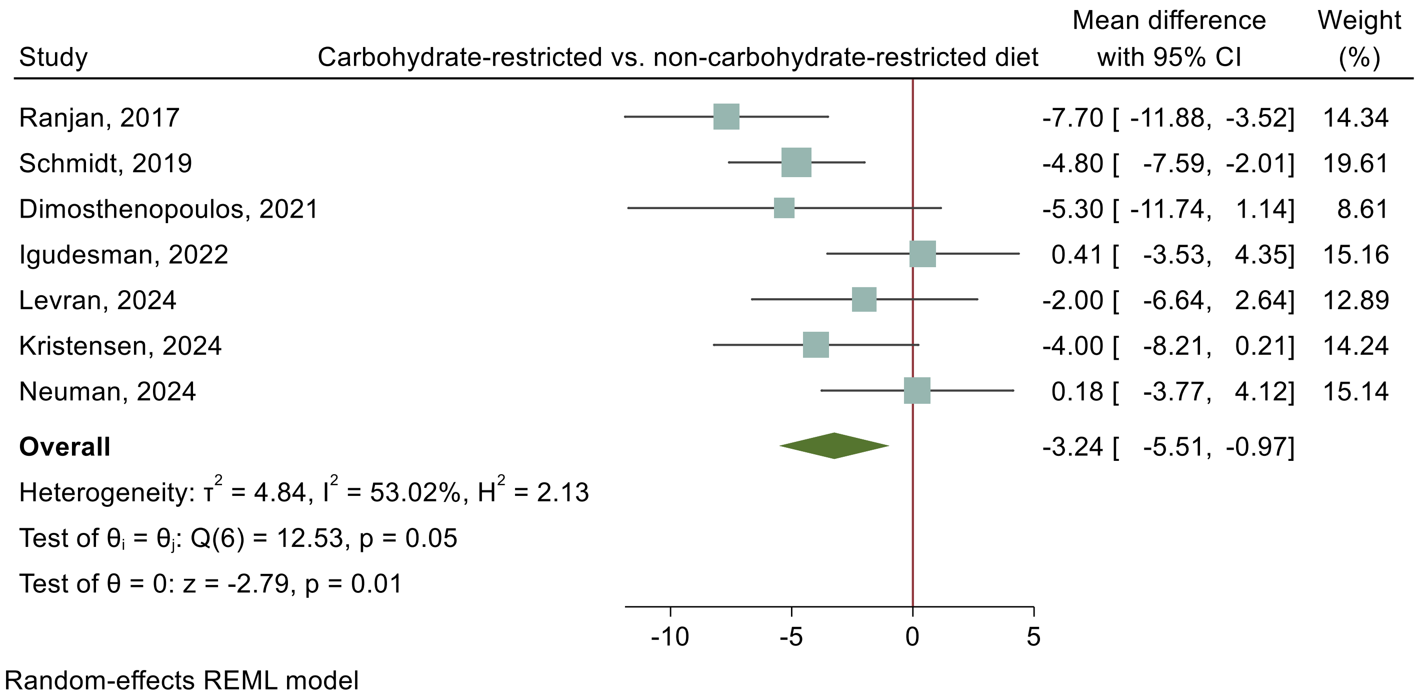

Supplementary Figure 6. Summary of mean difference in coefficient of variation (%) for carbohydrate-restricted versus non-carbohydrate-restricted diets.

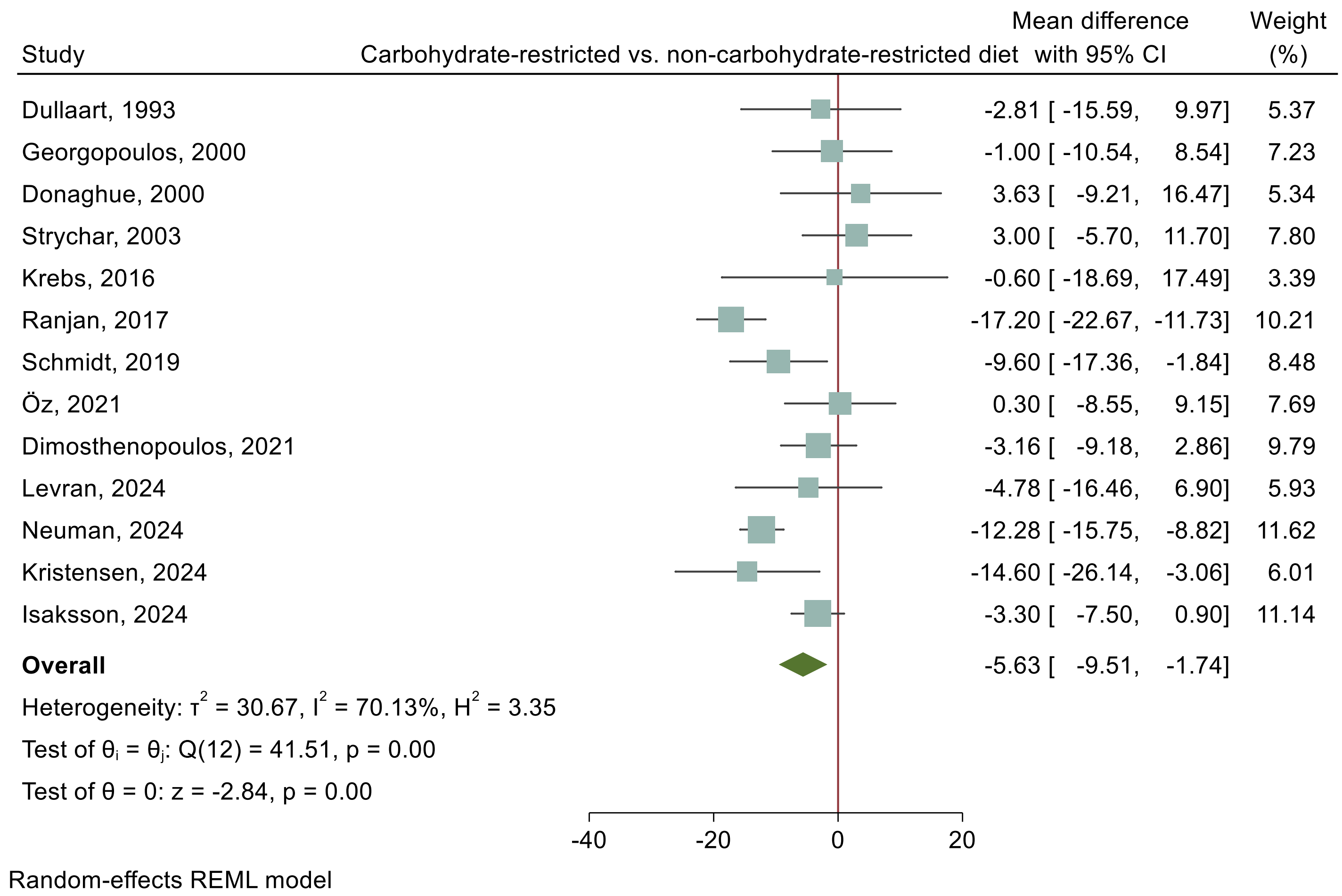

Supplementary Figure 7. Summary of mean difference in insulin dose (U/day) for carbohydrate-restricted versus non-carbohydrate-restricted diets.

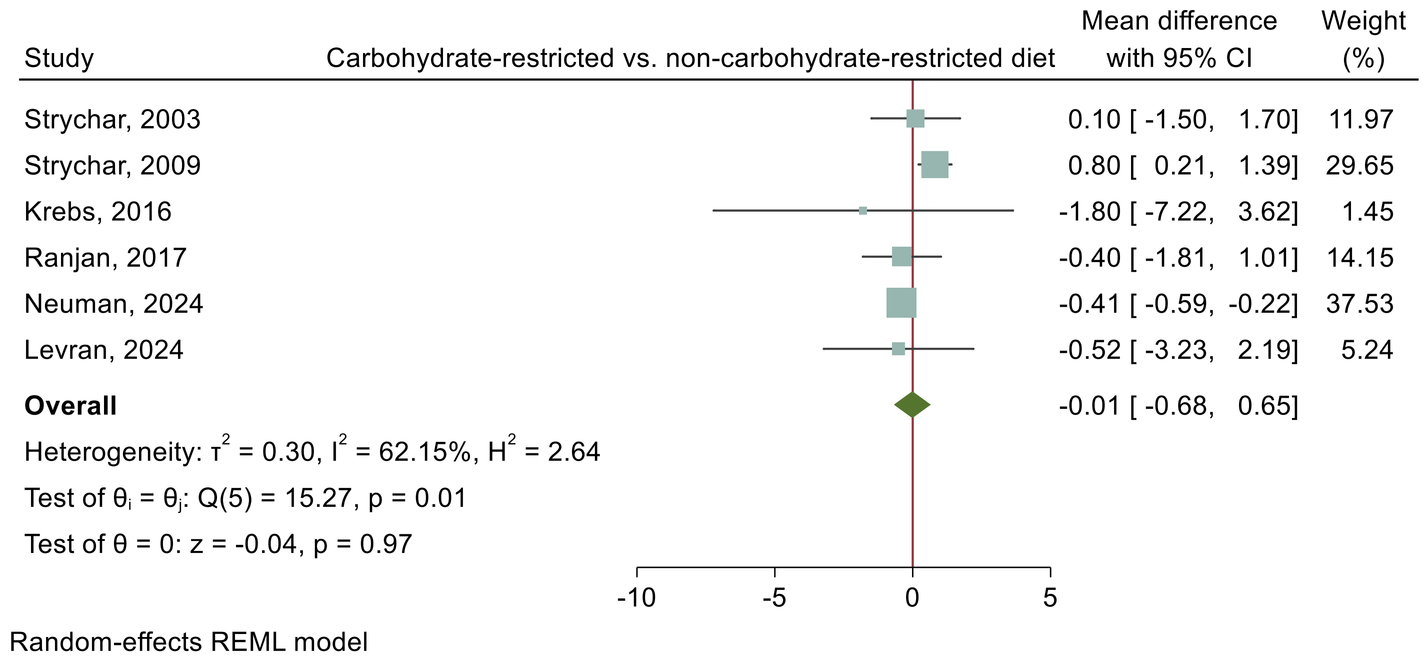

Supplementary Figure 8. Summary of mean difference in body mass index (kg/m^2) for carbohydrate-restricted versus non-carbohydrate-restricted diets.

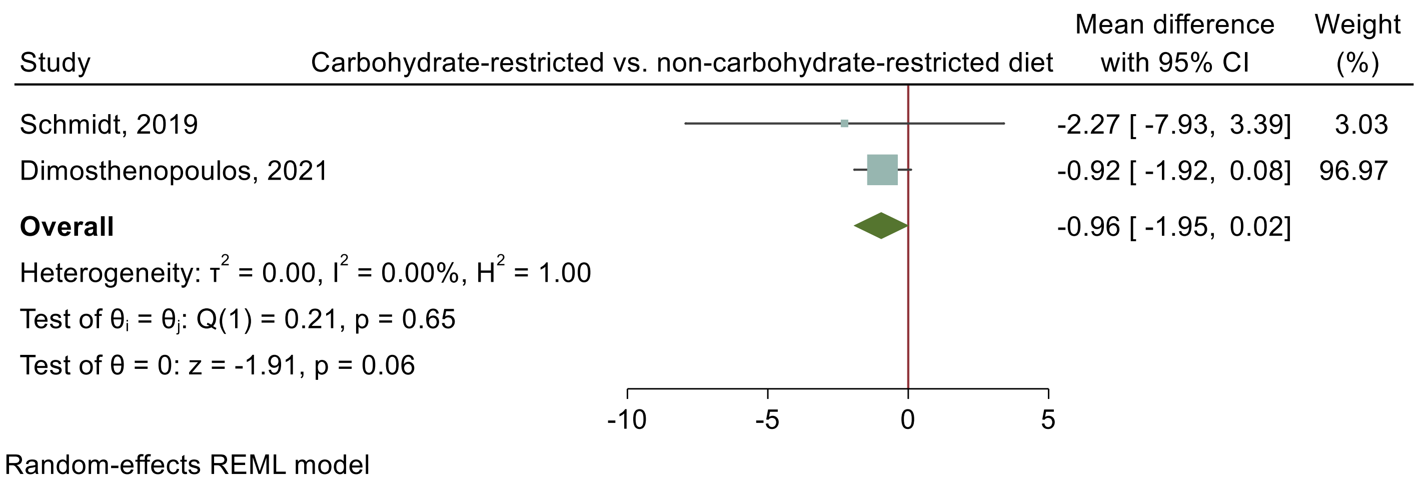

Supplementary Figure 9. Summary of mean difference in waist circumference (cm) for carbohydrate-restricted versus non-carbohydrate-restricted diets.

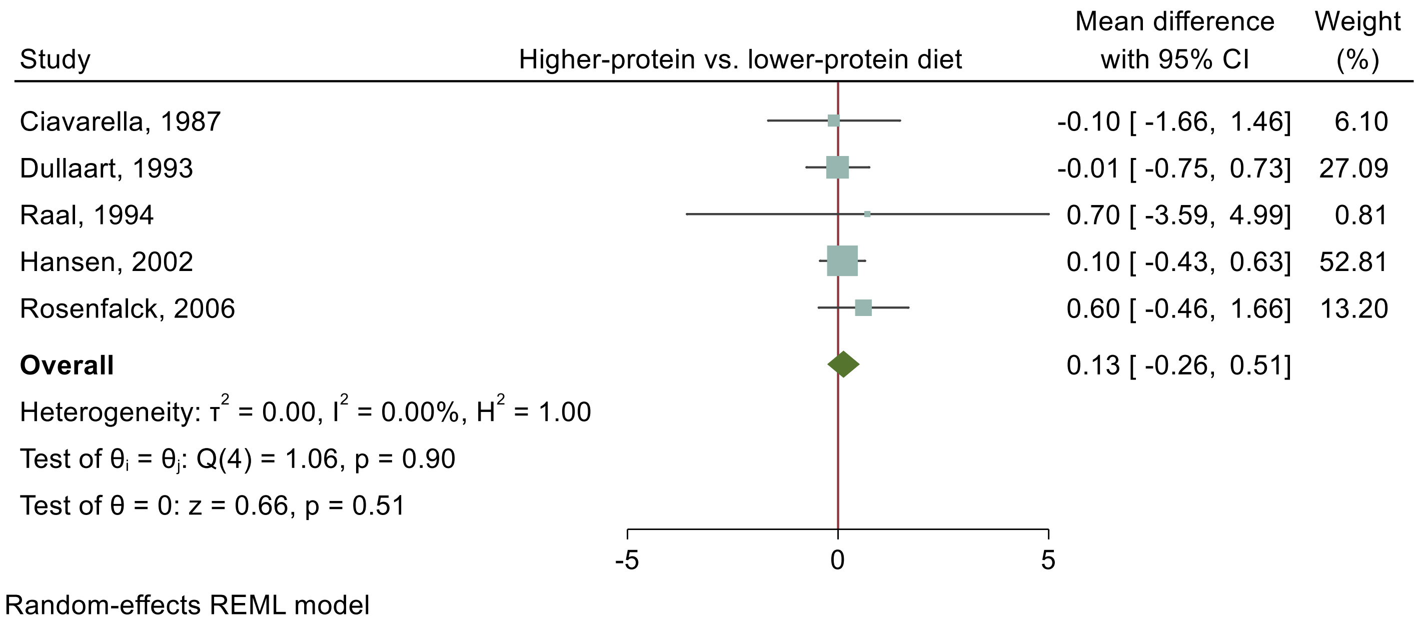

Supplementary Figure 10. Summary of mean difference in HbA1c (%) for higher-protein versus lower-protein diets.

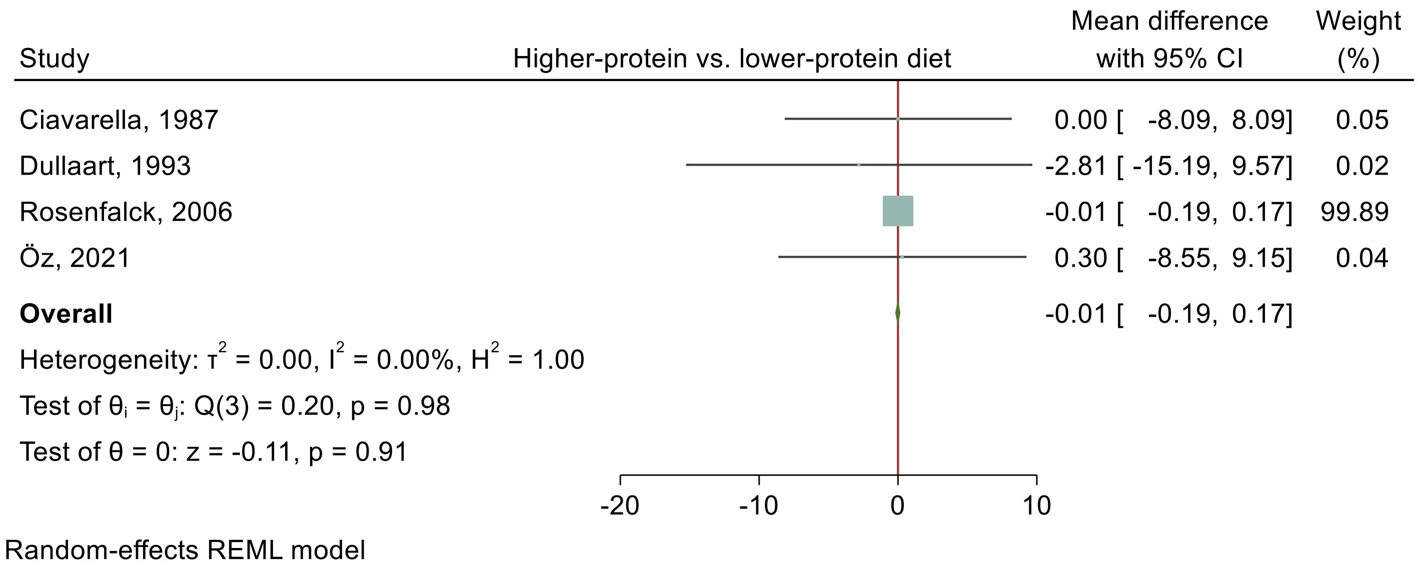

Supplementary Figure 11. Summary of mean difference in insulin dose (U/day) for higher-protein versus lower-protein diets.

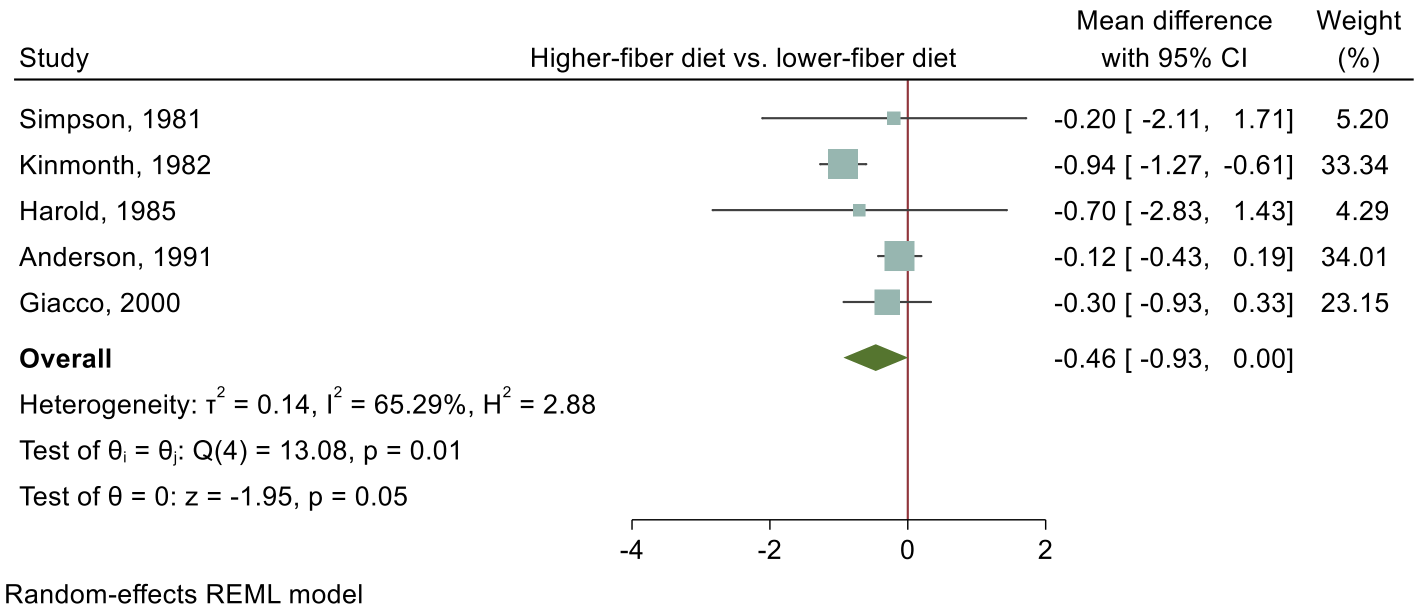

Supplementary Figure 12. Summary of mean difference in HbA1c (%) for higher-fiber versus lower-fiber diets.

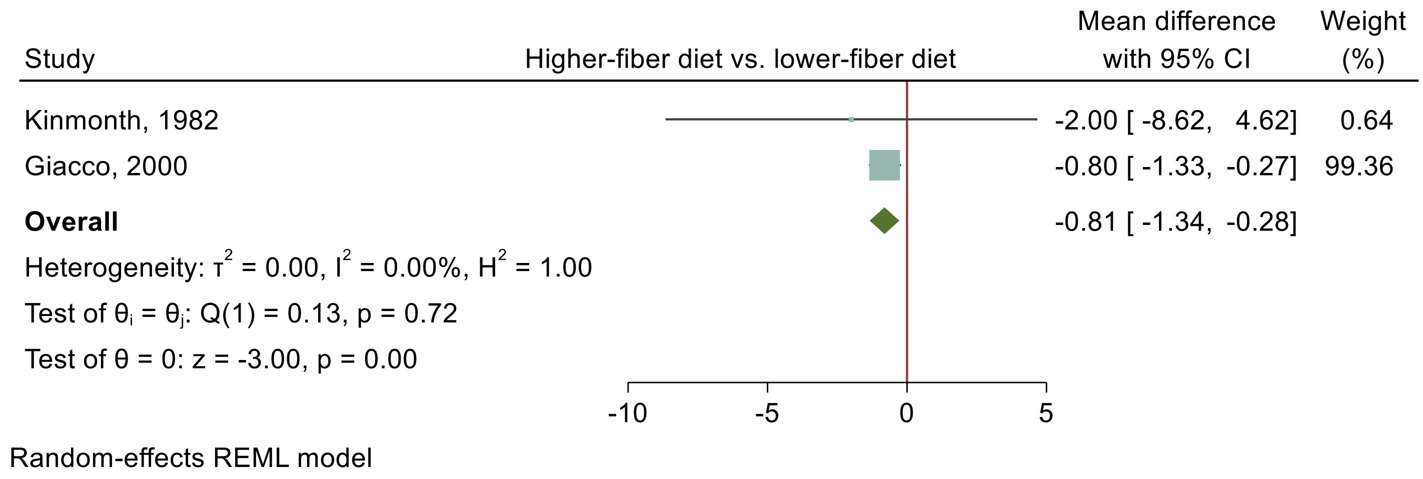

Supplementary Figure 13. Summary of mean difference in number of hypoglycemic episodes per month for higher-fiber versus lower-fiber diets.

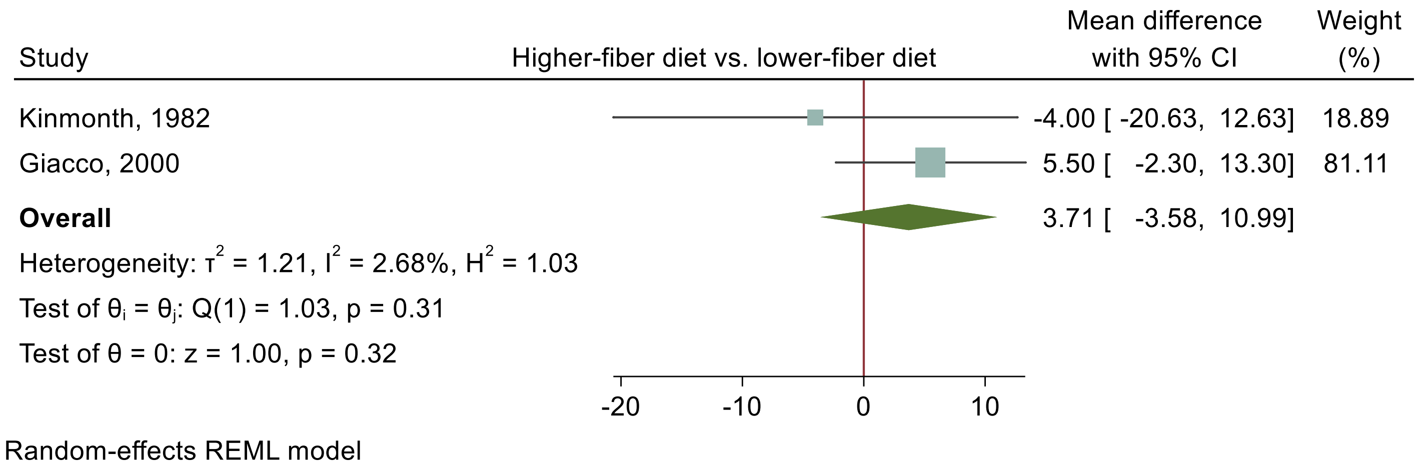

Supplementary Figure 14. Summary of mean difference in insulin dose (U/day) for higher-fiber versus lower-fiber diets diets.

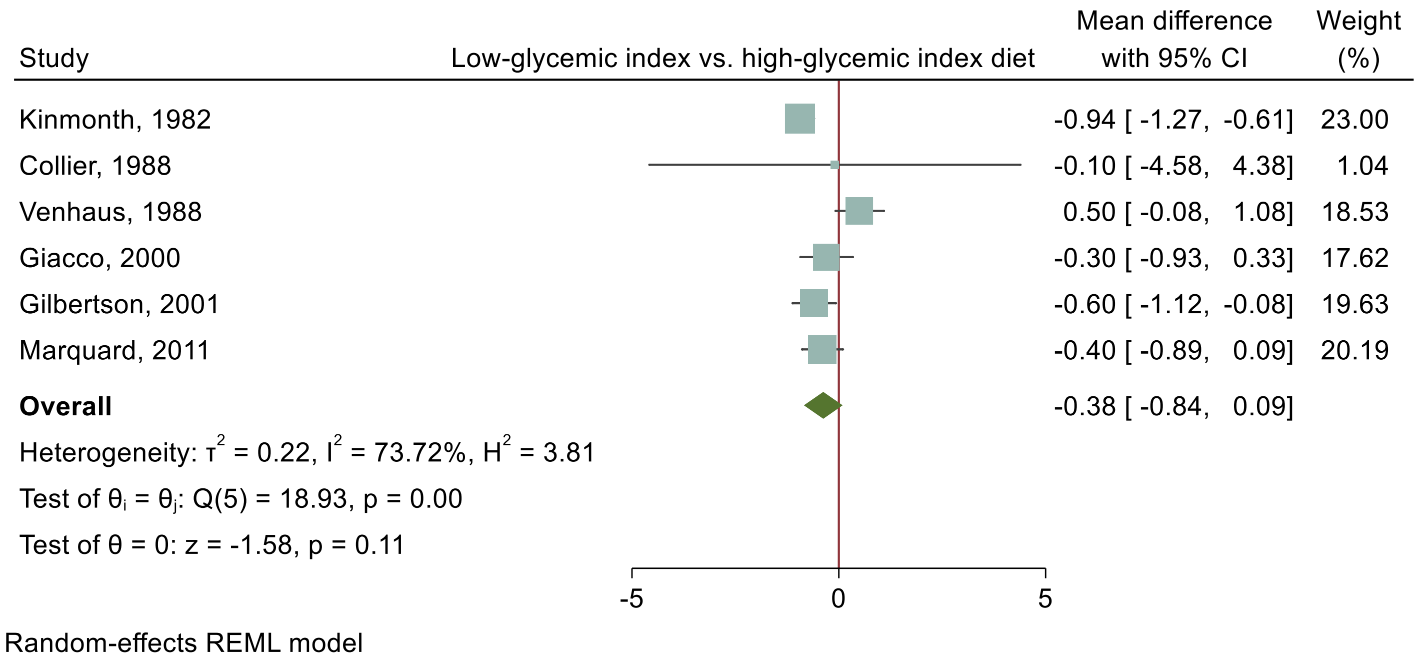

Supplementary Figure 15. Summary of mean difference in HbA1c (%) for low-glycemic index versus high-glycemic index diets.

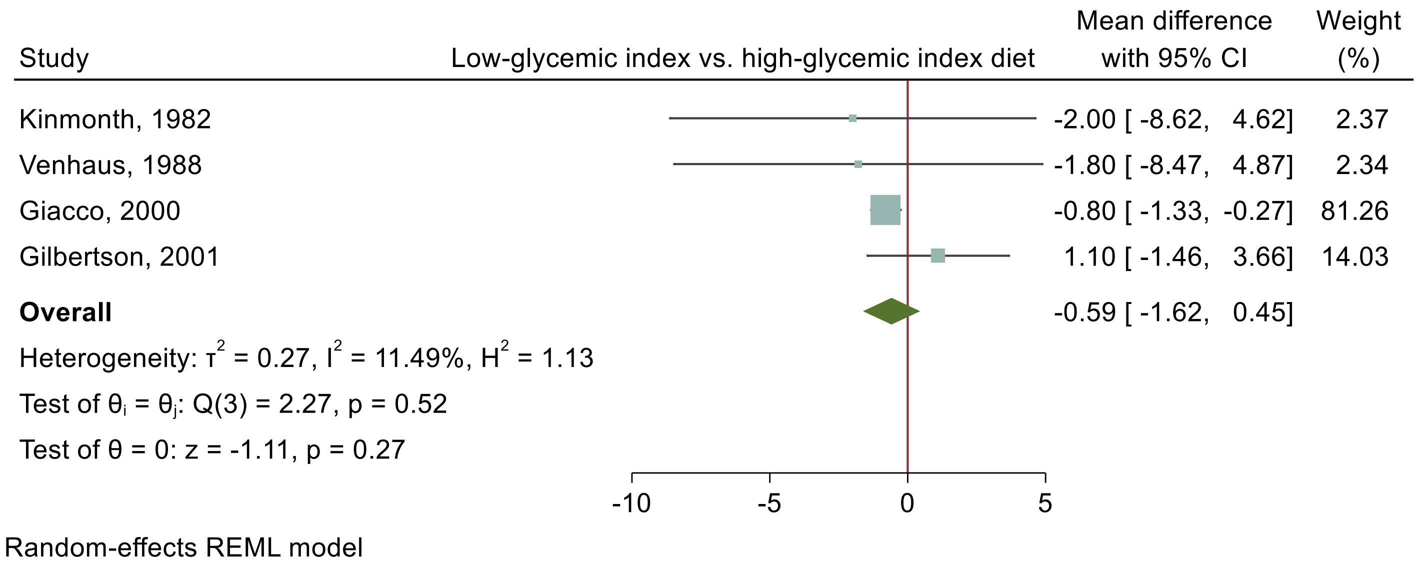

Supplementary Figure 16. Summary of mean difference in number of hypoglycemic episodes per month for low-glycemic index versus high-glycemic index diets.

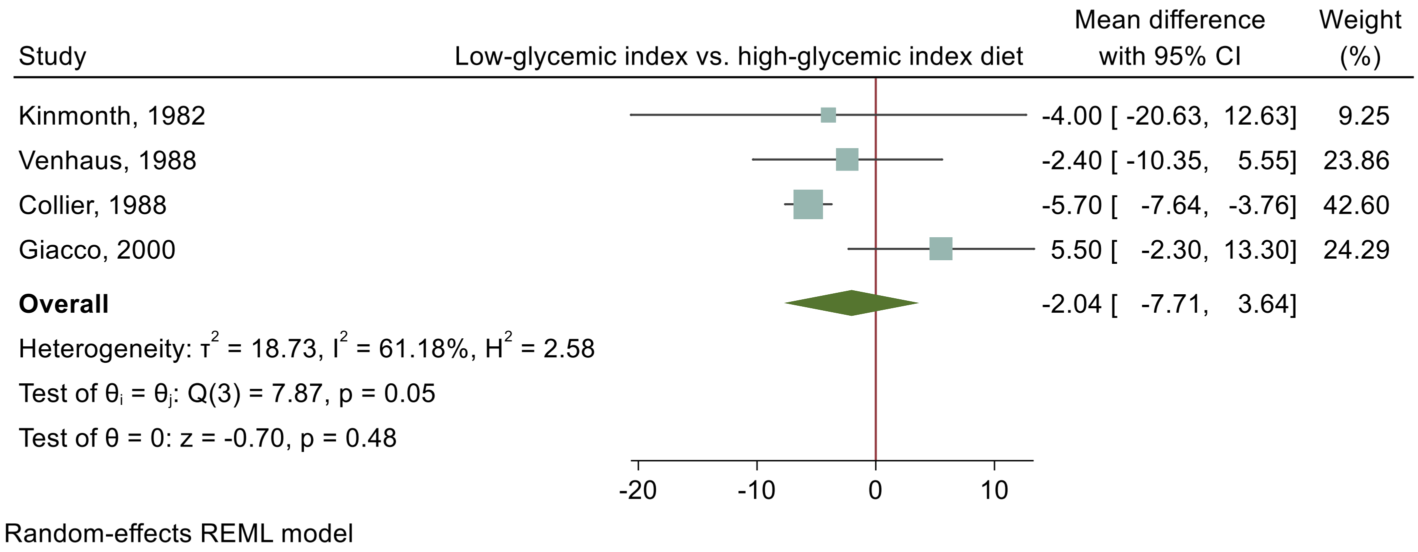

Supplementary Figure 17. Summary of mean difference in insulin dose (U/day) for low-glycemic index versus high-glycemic index diets.

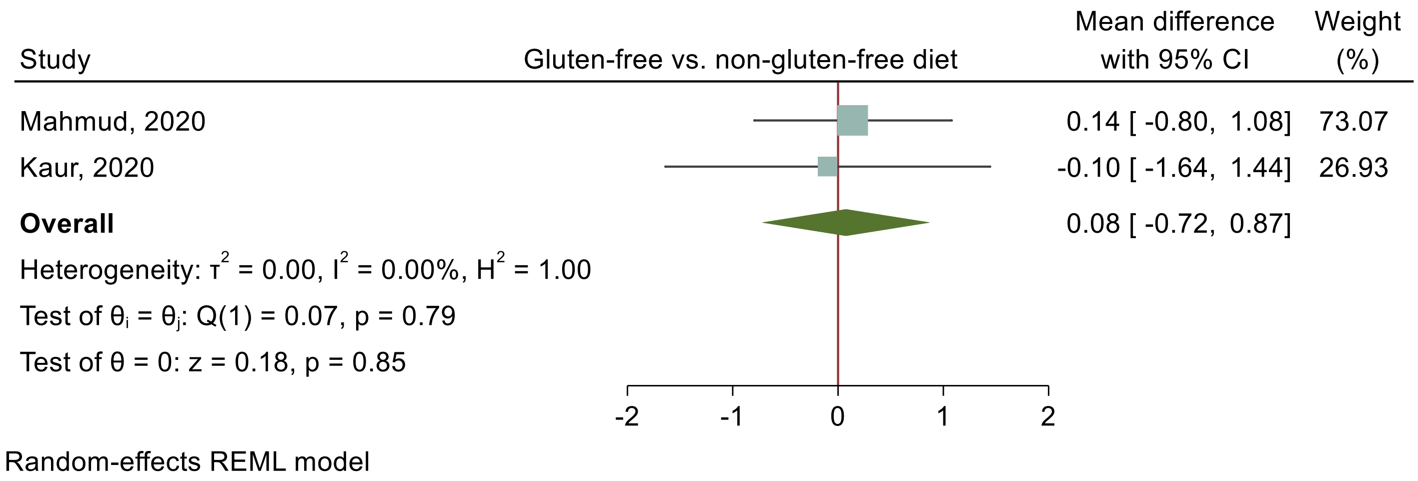

Supplementary Figure 18. Summary of mean difference in HbA1c (%) for gluten-free versus non-gluten-free diets.

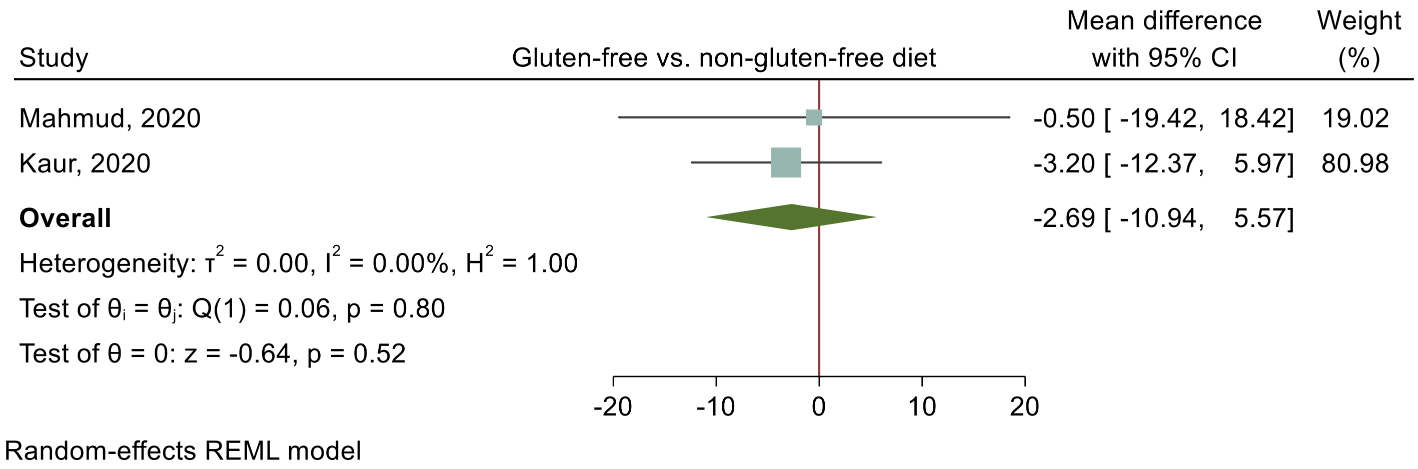

Supplementary Figure 19. Summary of mean difference in insulin dose (U/day) for gluten-free versus non-gluten-free diets.

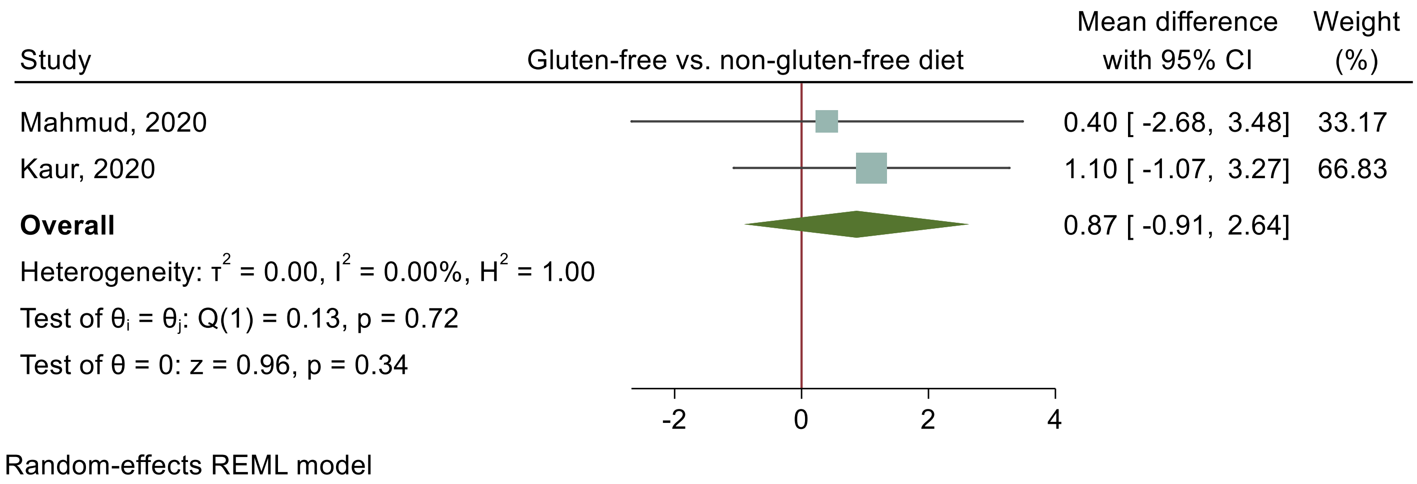

Supplementary Figure 20. Summary of mean difference in body mass index (kg/m^2) for gluten-free versus non-gluten-free diets.

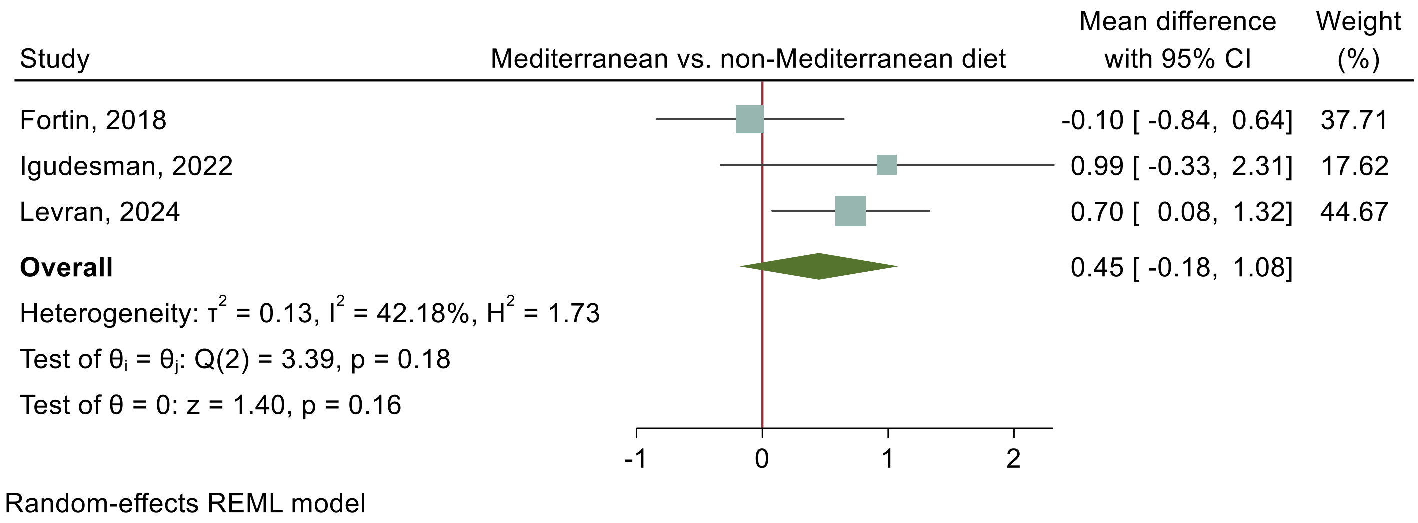

Supplementary Figure 21. Summary of mean difference in HbA1c (%) for Mediterranean versus non-Mediterranean diets.

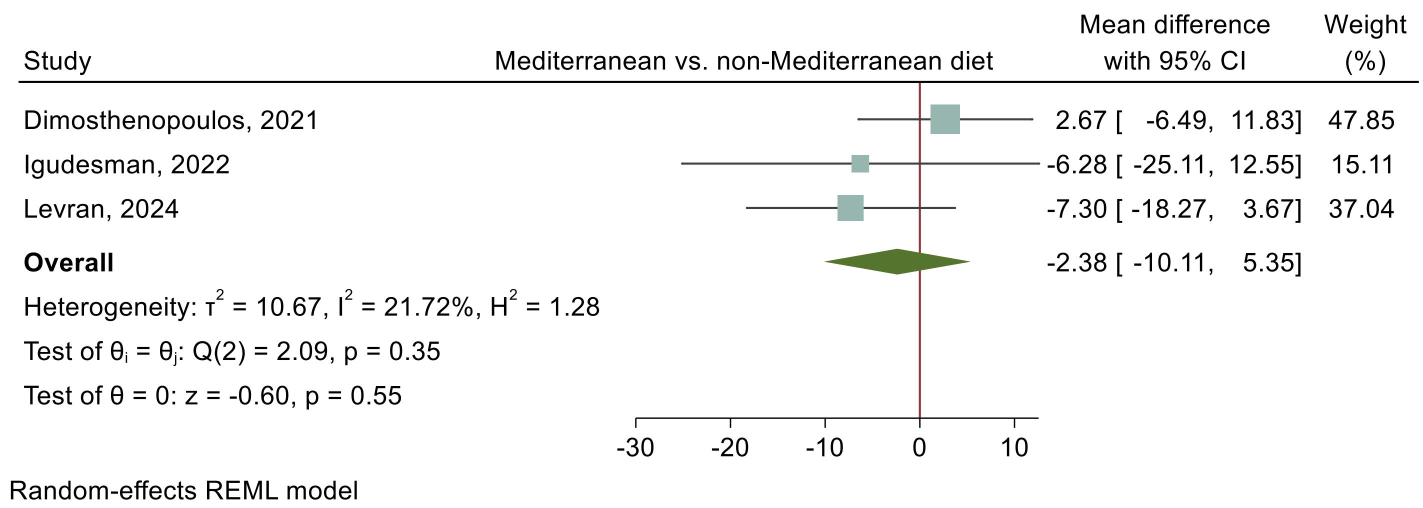

Supplementary Figure 22. Summary of mean difference in time in range (%) for Mediterranean versus non-Mediterranean diets.

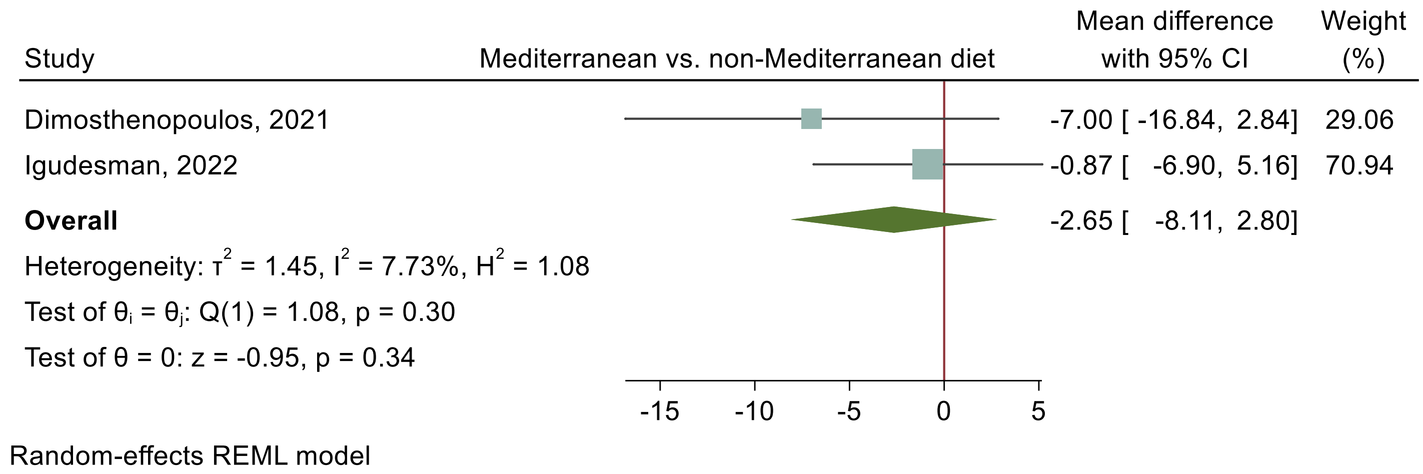

Supplementary Figure 23. Summary of mean difference in time below range (%) for Mediterranean versus non-Mediterranean diets.

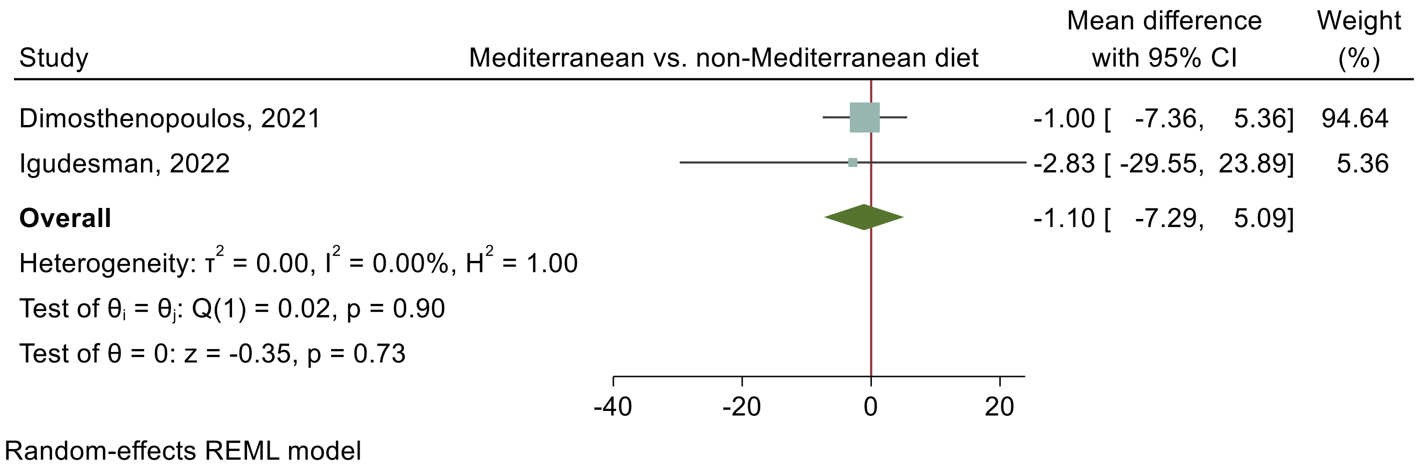

Supplementary Figure 24. Summary of mean difference in time above range (%) for Mediterranean versus non-Mediterranean diets.

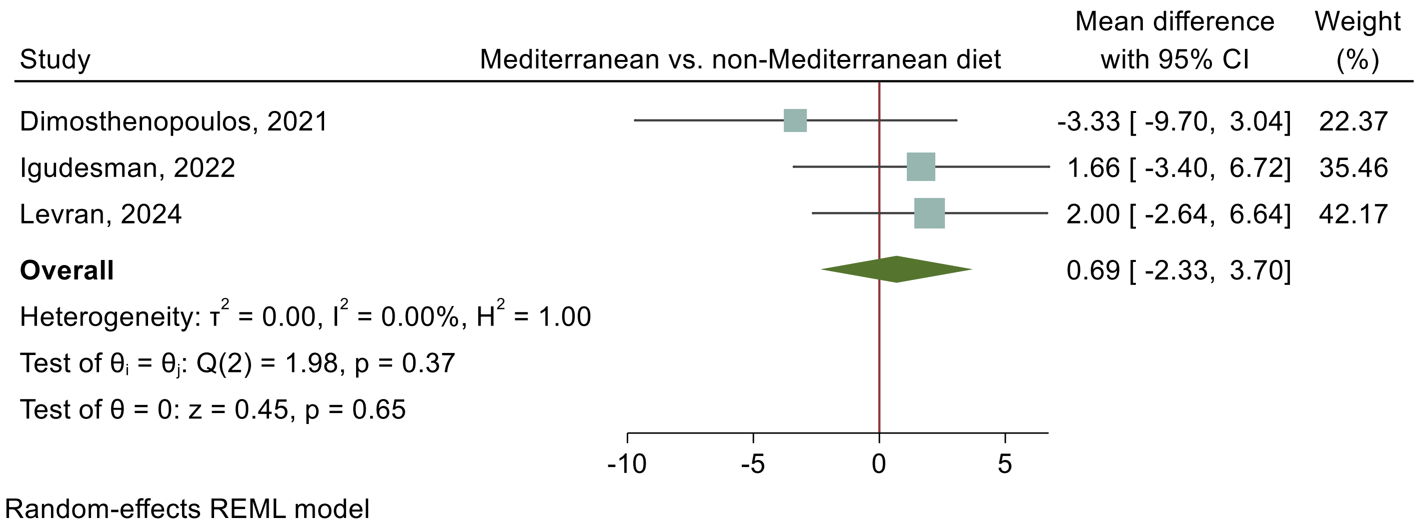

Supplementary Figure 25. Summary of mean difference in coefficient of variation (%) for Mediterranean versus non-Mediterranean diets.

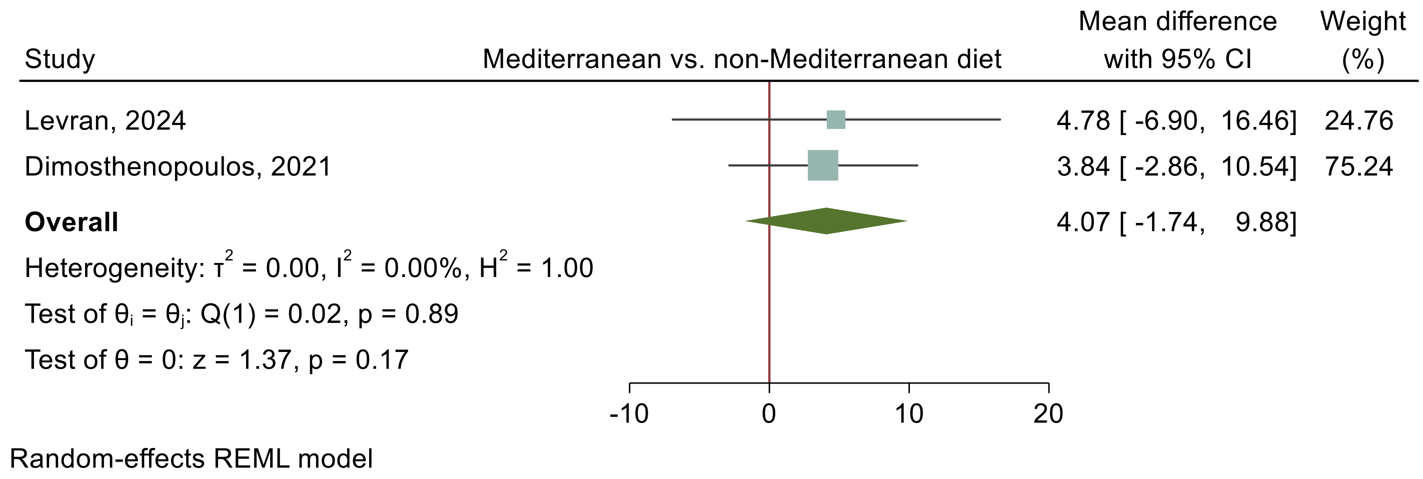

Supplementary Figure 26. Summary of mean difference in insulin dose (U/day) for Mediterranean versus non-Mediterranean diets.

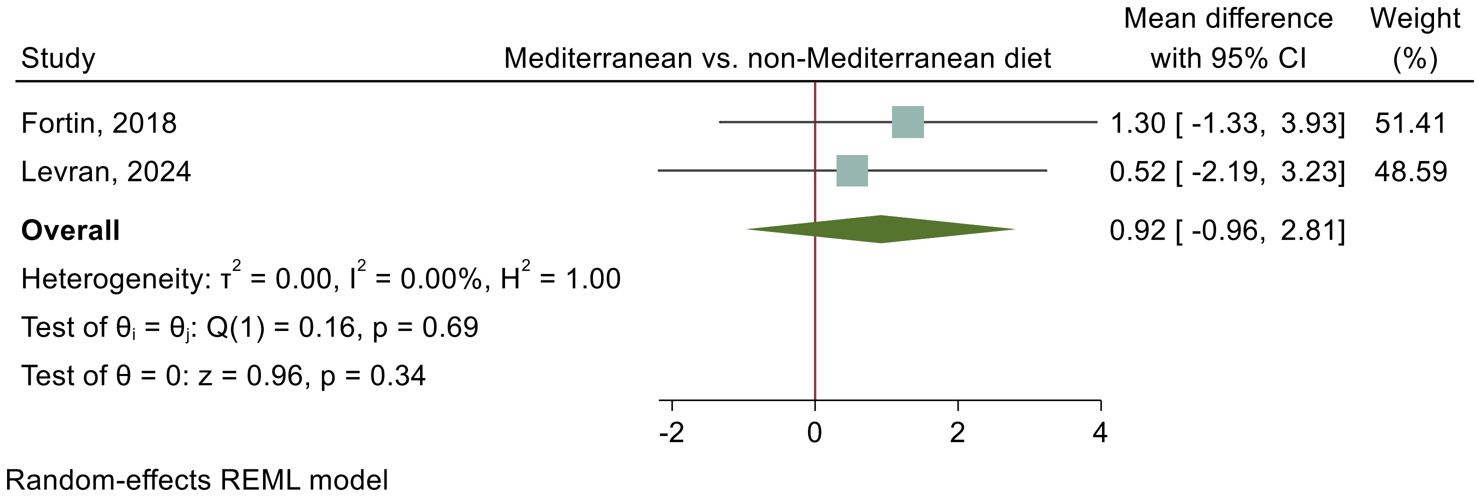

Supplementary Figure 27. Summary of mean difference in body mass index (kg/m^2) for Mediterranean versus non-Mediterranean diets.

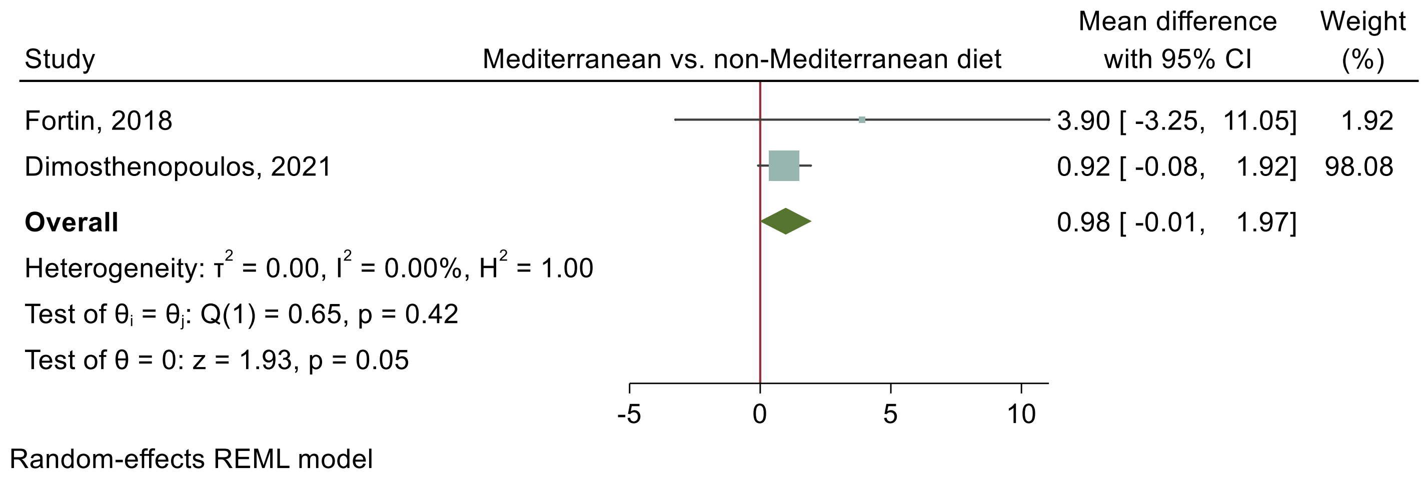

Supplementary Figure 28. Summary of mean difference in waist circumference (cm) for Mediterranean versus non-Mediterranean diets.

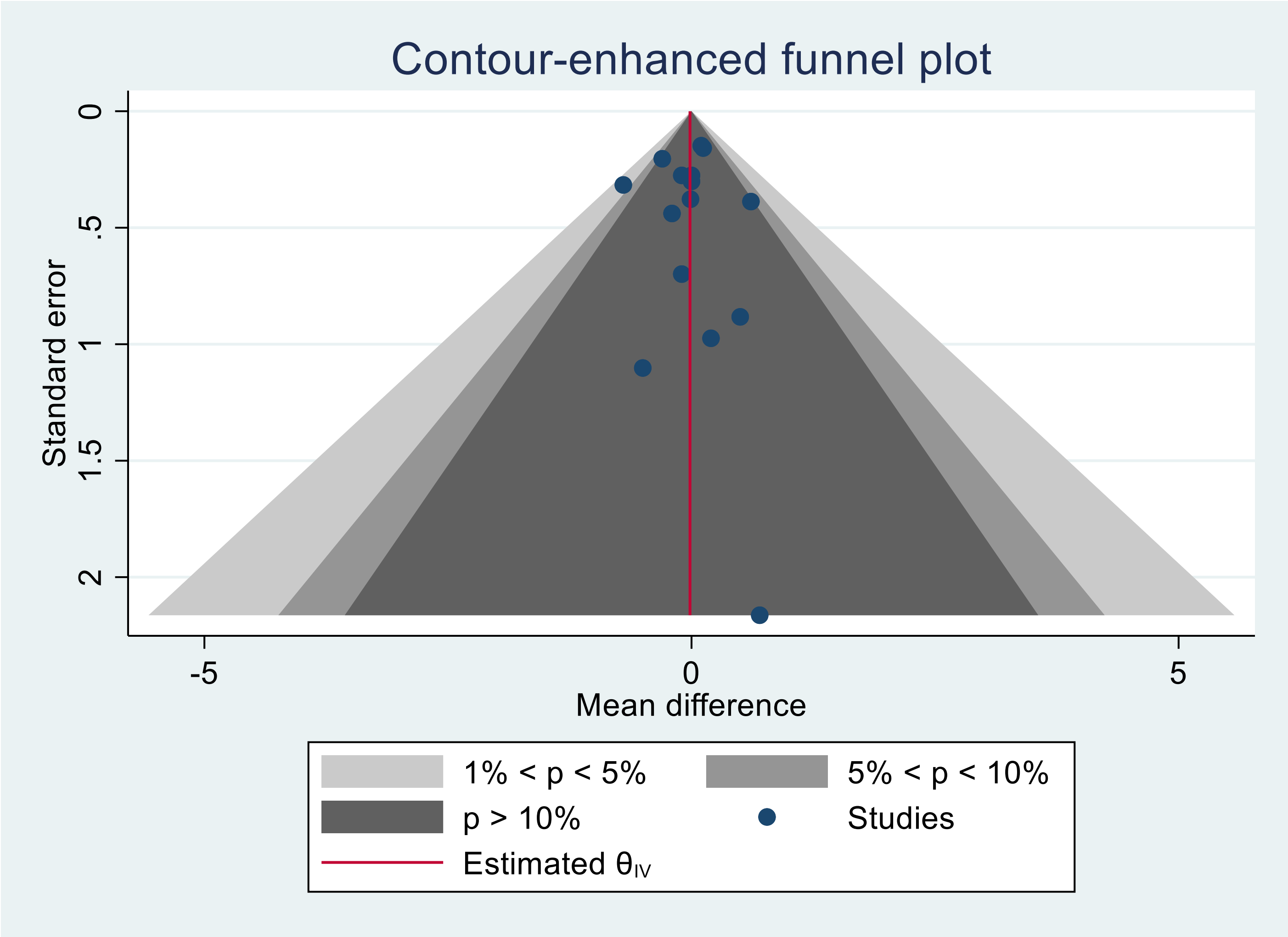

Supplementary Figure 29. Contour-enhanced funnel plot of studies on carbohydrate-restricted versus non-carbohydrate-restricted diets and HbA1c change. Egger's test *P* = 0.9601

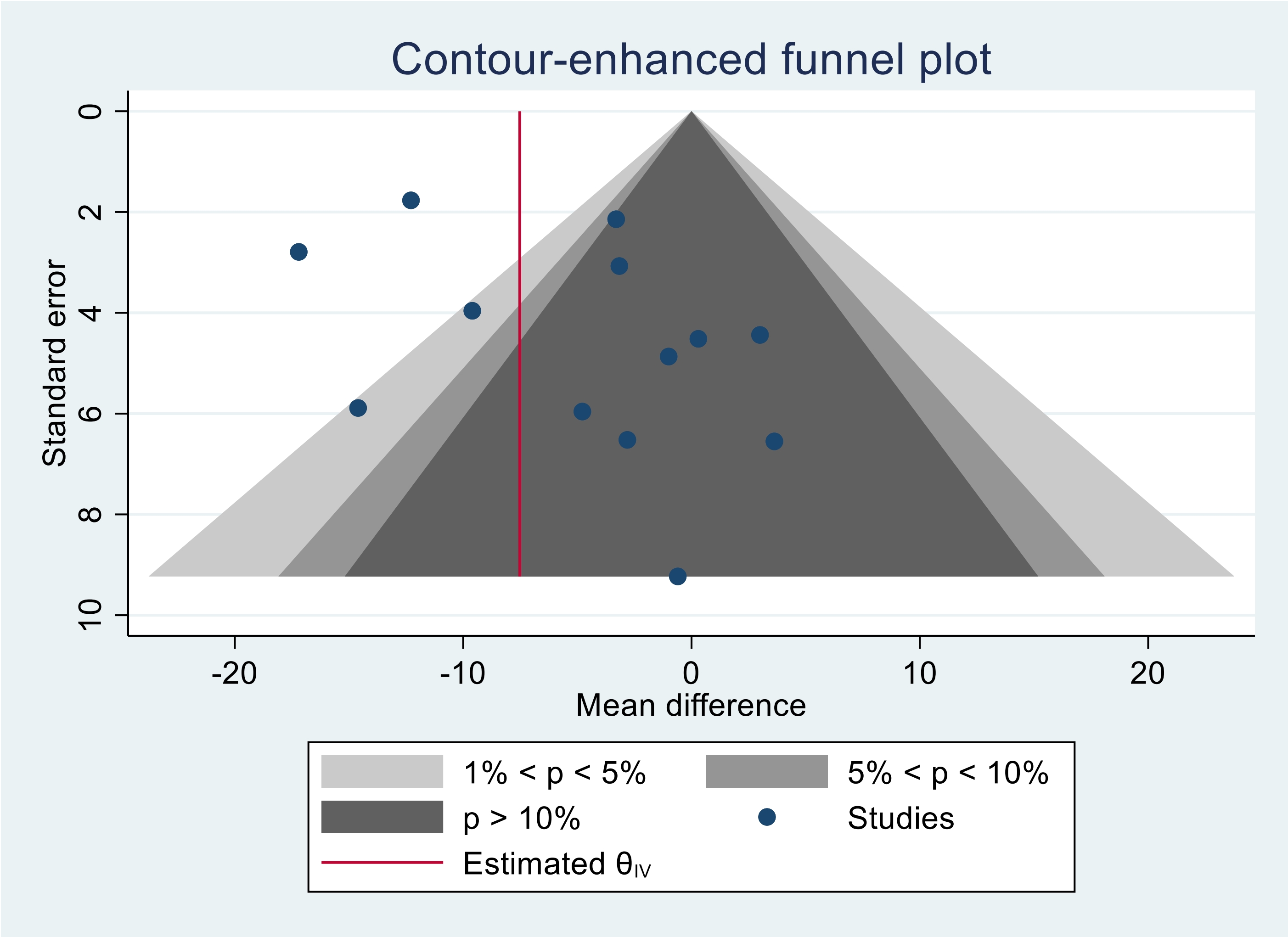

Supplementary Figure 30. Contour-enhanced funnel plot of studies on carbohydrate-restricted versus non-carbohydrate-restricted diets and insulin change. Egger's test *P* = 0.1510

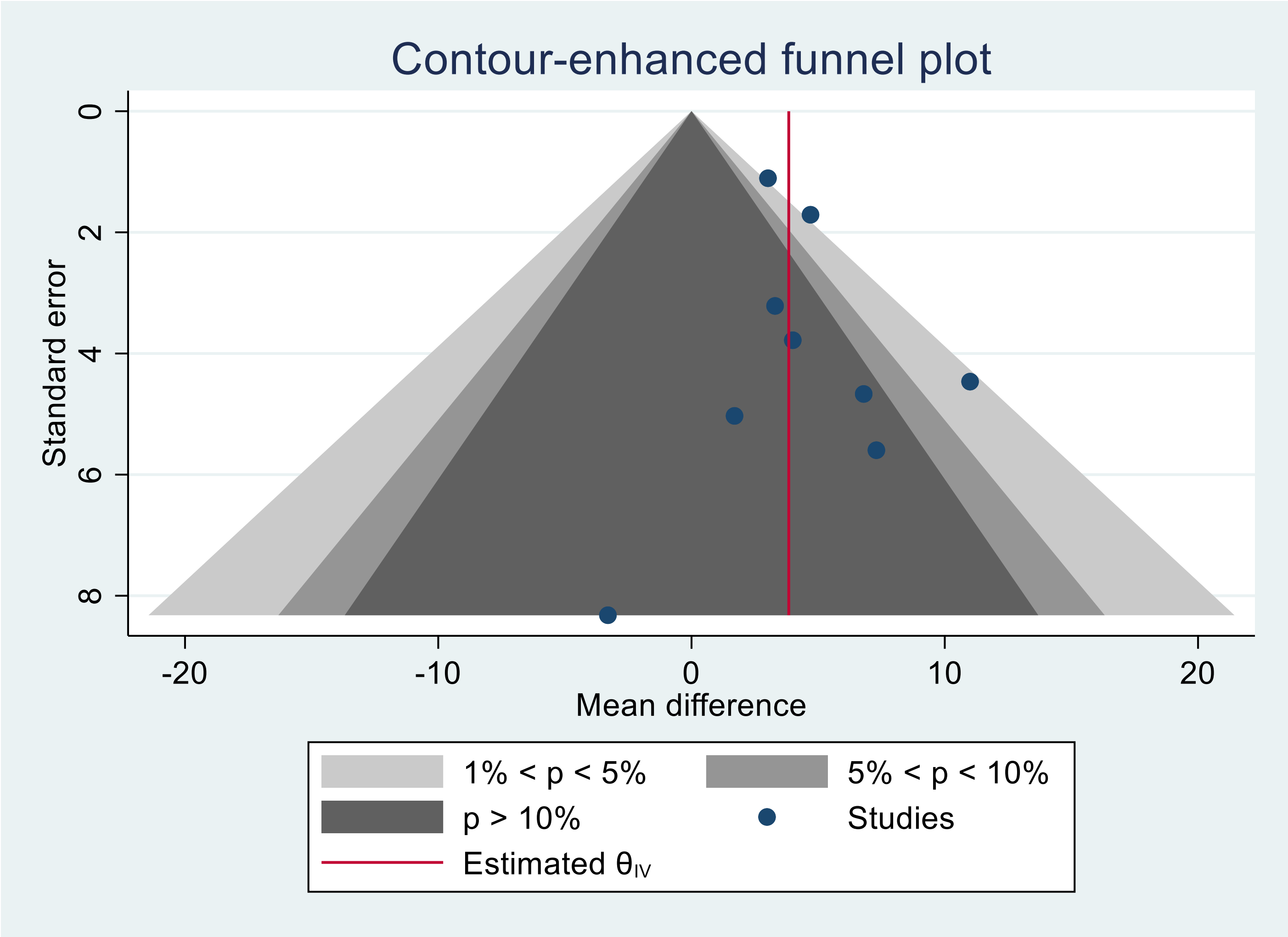

Supplementary Figure 31. Contour-enhanced funnel plot of studies on carbohydrate-restricted versus non-carbohydrate-restricted diets and time in range change.

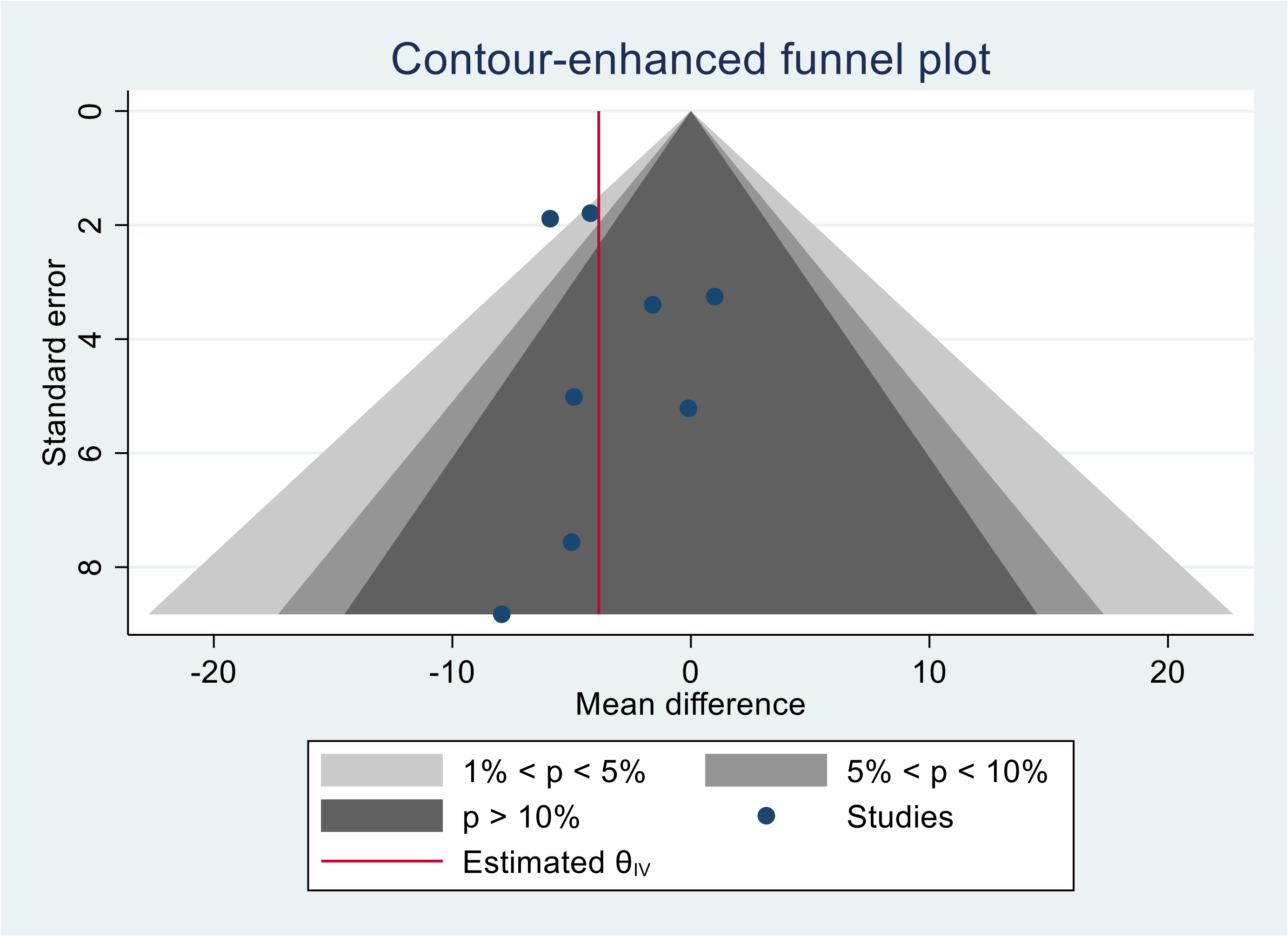

Supplementary Figure 32. Contour-enhanced funnel plot of studies on carbohydrate-restricted versus non-carbohydrate-restricted diets and time above range change.

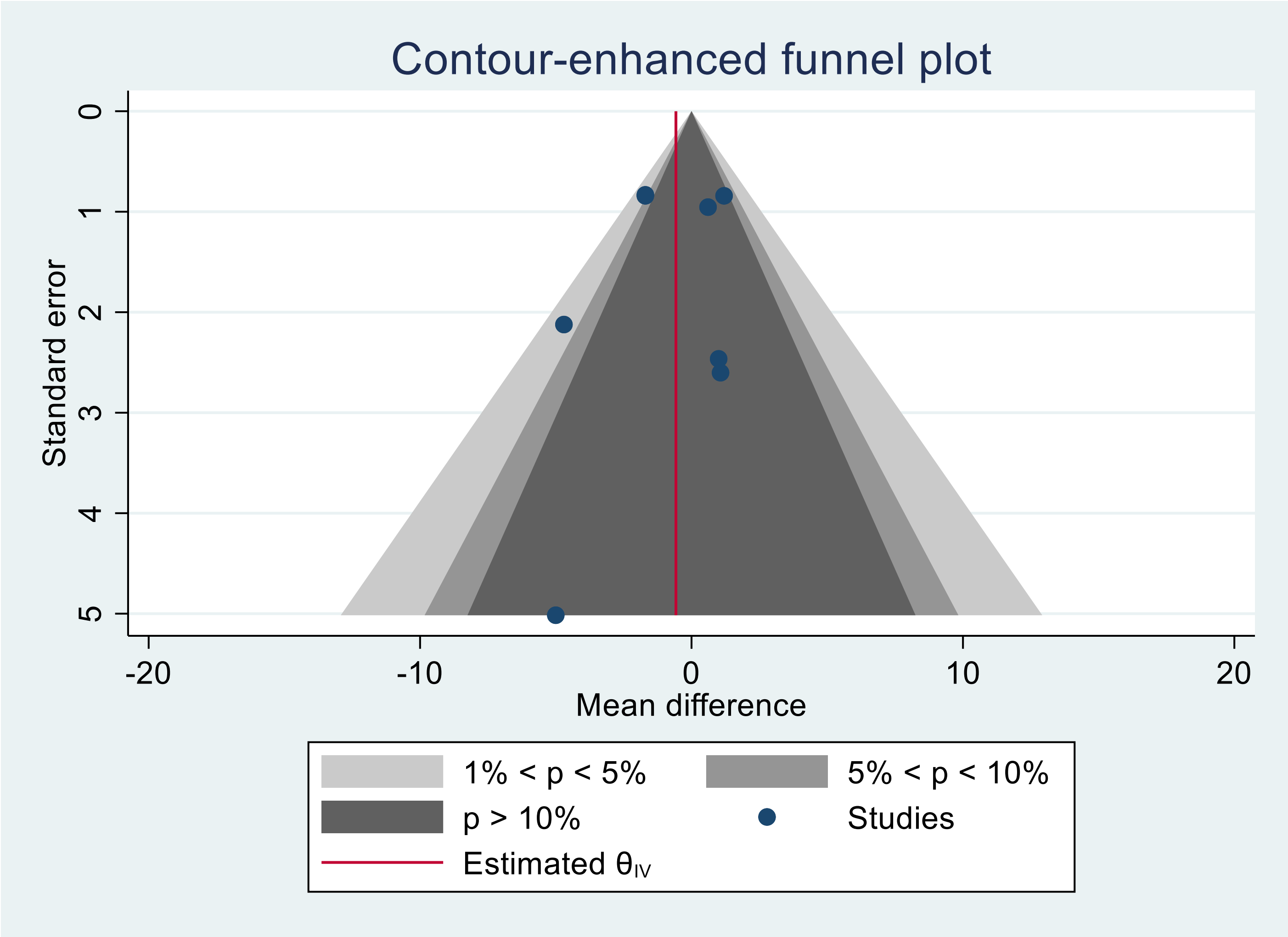

Supplementary Figure 33. Contour-enhanced funnel plot of studies on carbohydrate-restricted versus non-carbohydrate-restricted diets and time below range change.

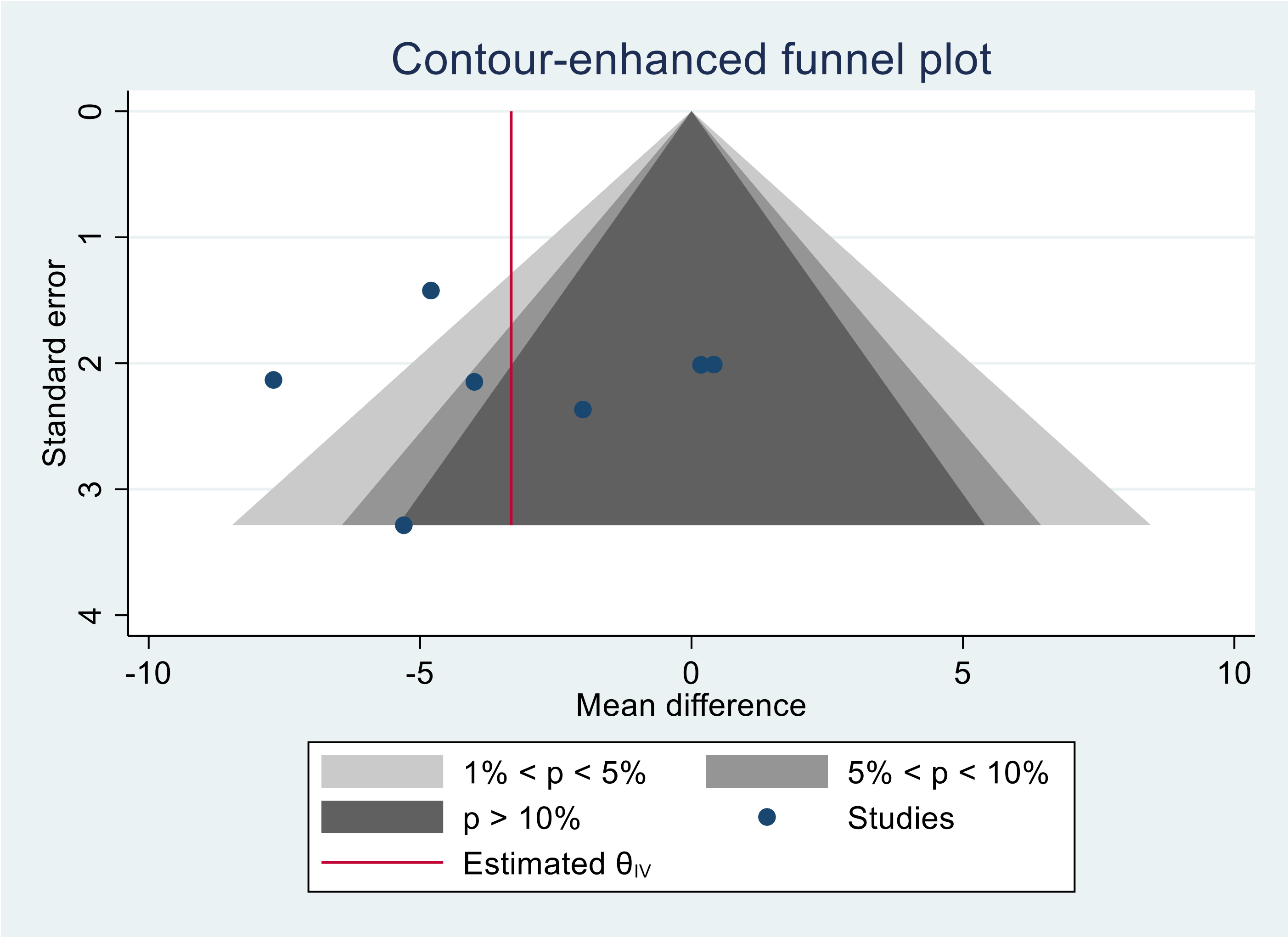

Supplementary Figure 34. Contour-enhanced funnel plot of studies on carbohydrate-restricted versus non-carbohydrate-restricted diets and coefficient of variation change.

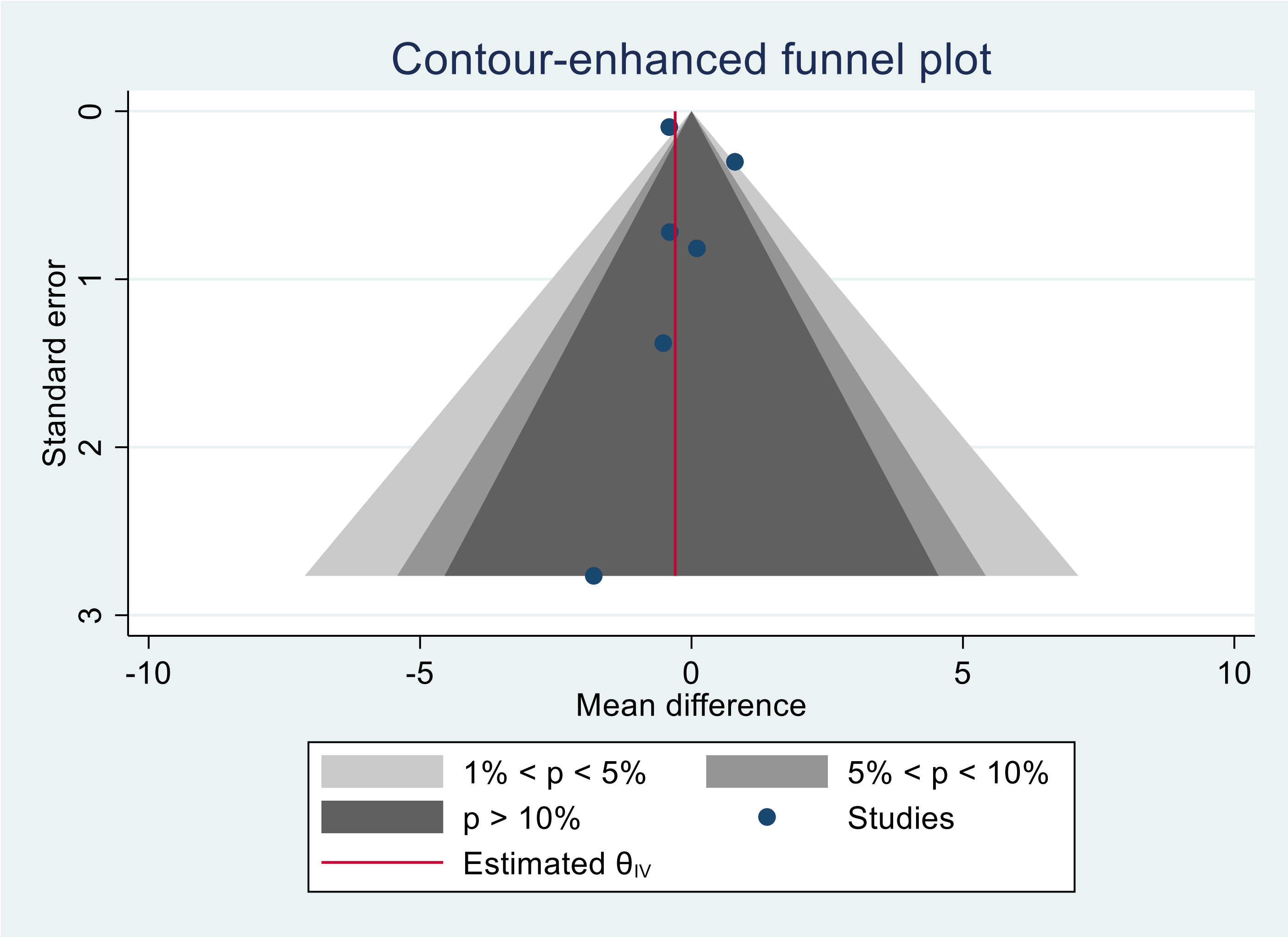

Supplementary Figure 35. Contour-enhanced funnel plot of studies on carbohydrate-restricted versus non-carbohydrate-restricted diets and BMI change.

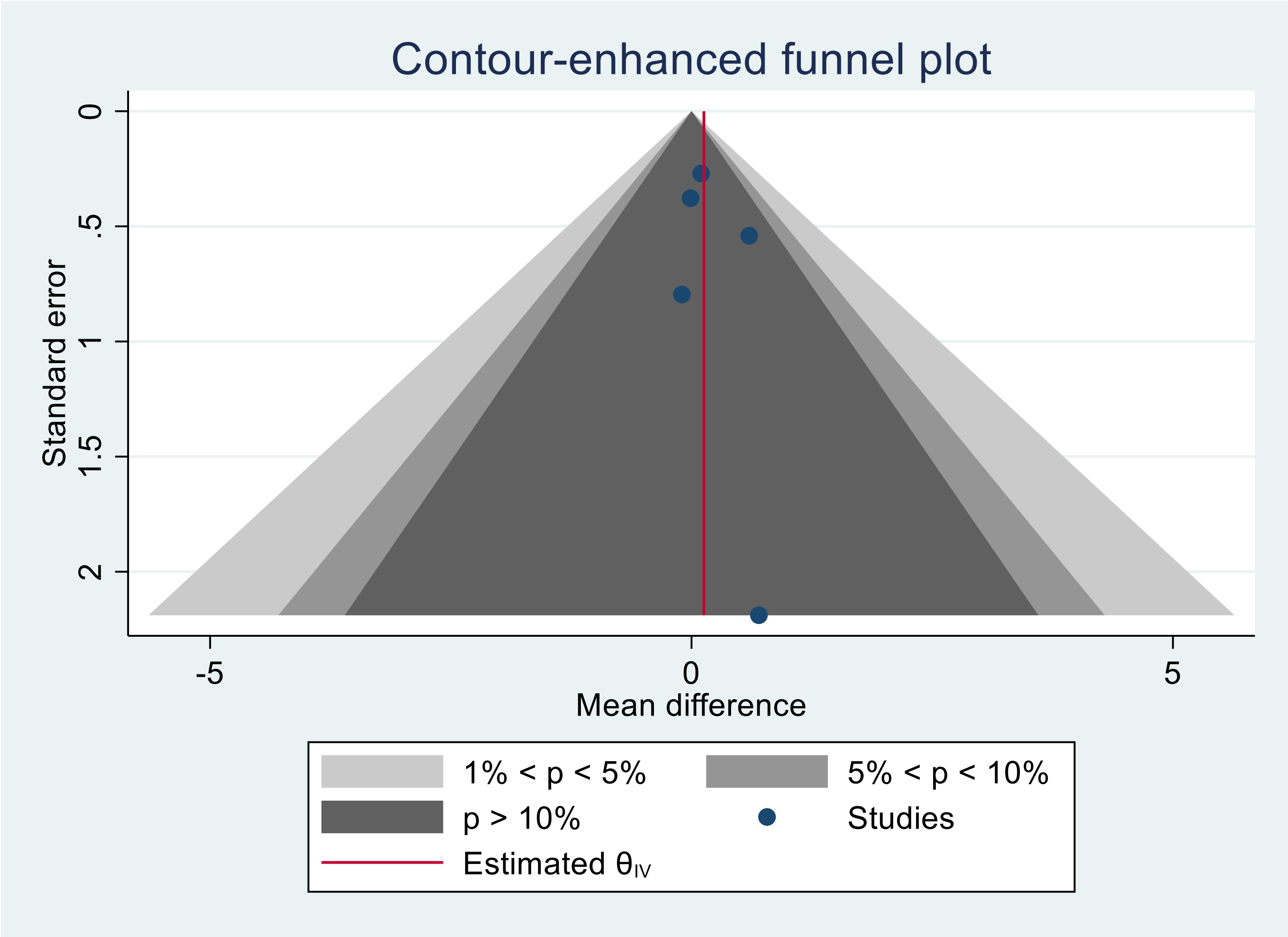

Supplementary Figure 36. Contour-enhanced funnel plot of studies on higher-protein versus lower-protein diets and HbA1c change.

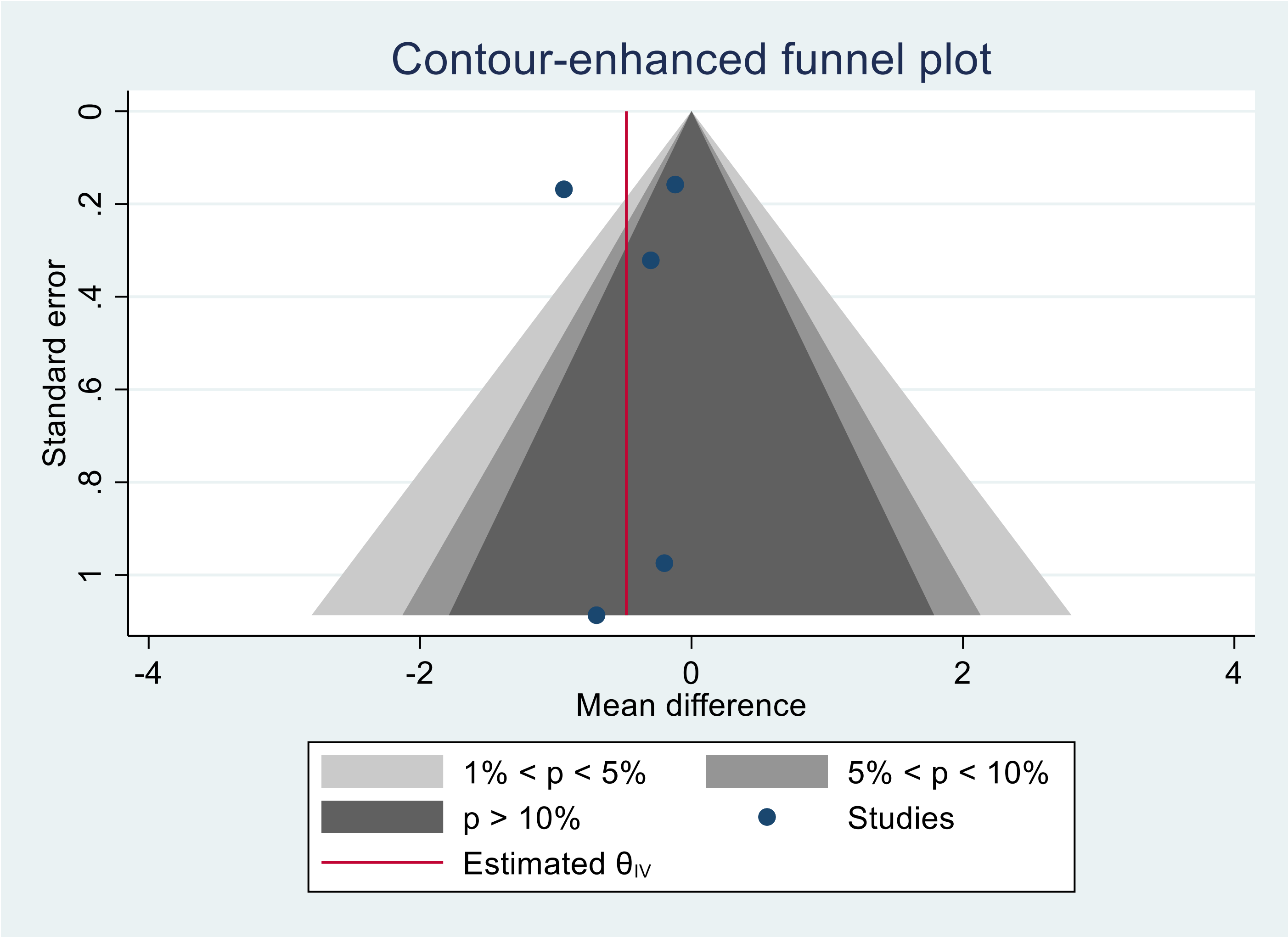

Supplementary Figure 37. Contour-enhanced funnel plot of studies on higher-fiber versus lower-fiber diets and HbA1c change.

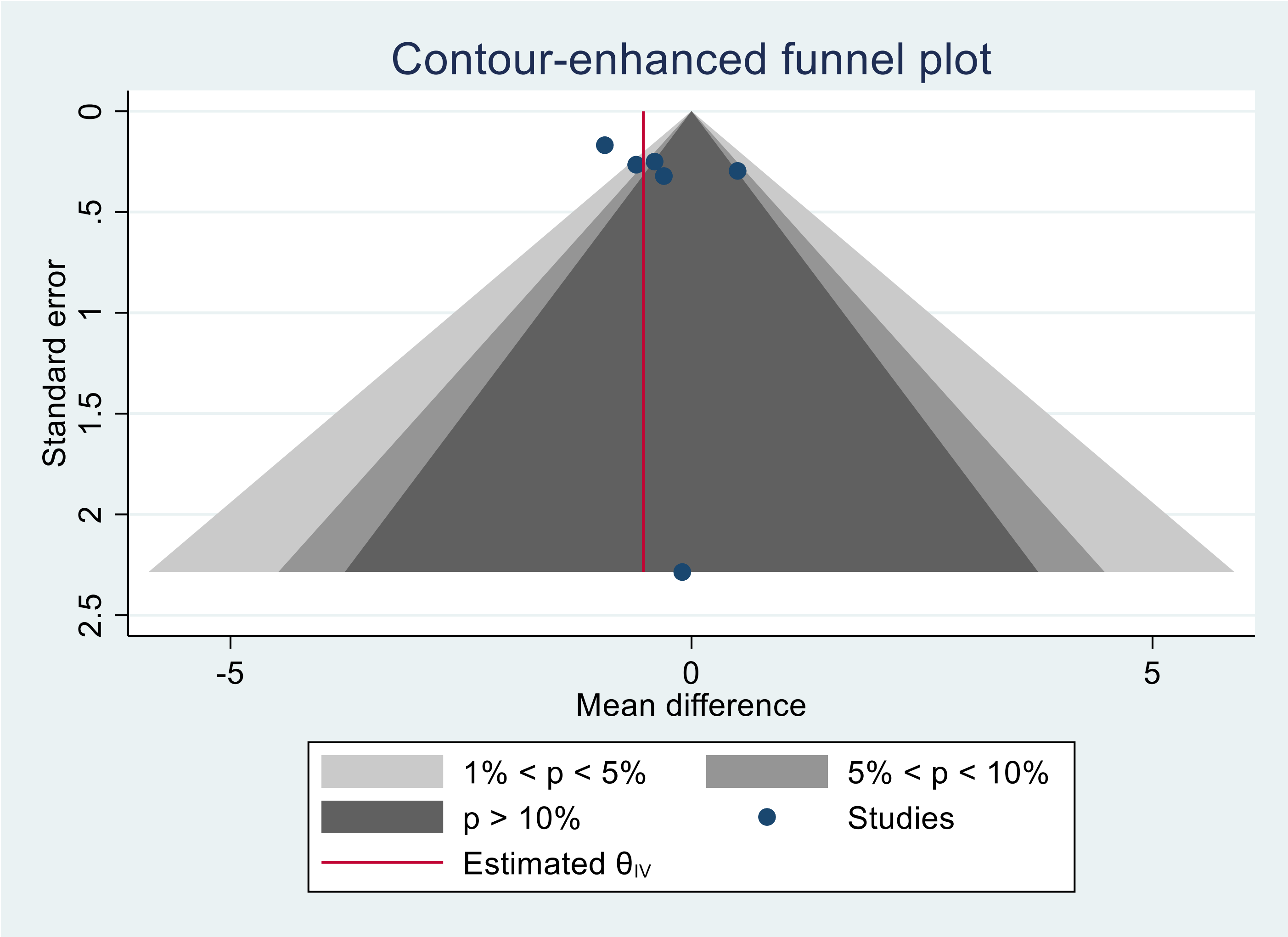

Supplementary Figure 38. Contour-enhanced funnel plot of studies on higher-glycemic index versus lower-glycemic index diets and HbA1c change.

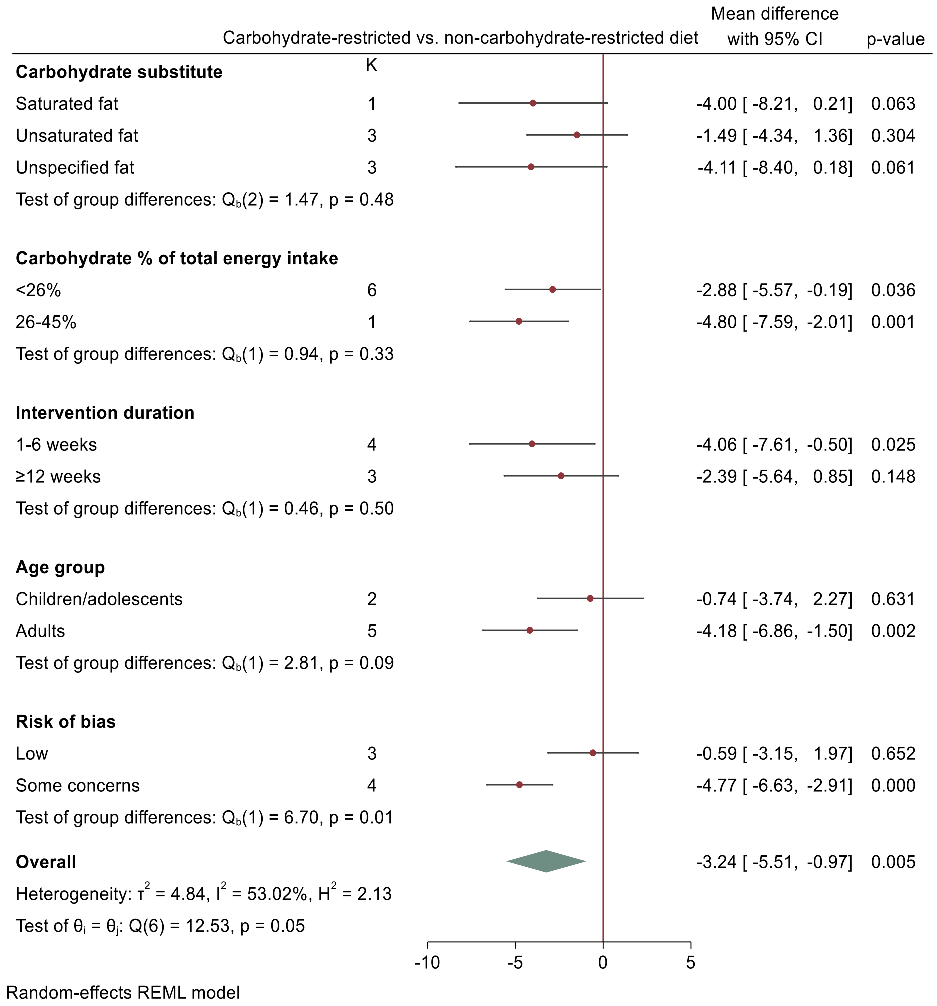

Supplementary Figure 39. Subgroup analyses for summary of mean difference in coefficient of variation (%) for carbohydrate-restricted versus non-carbohydrate-restricted diets.

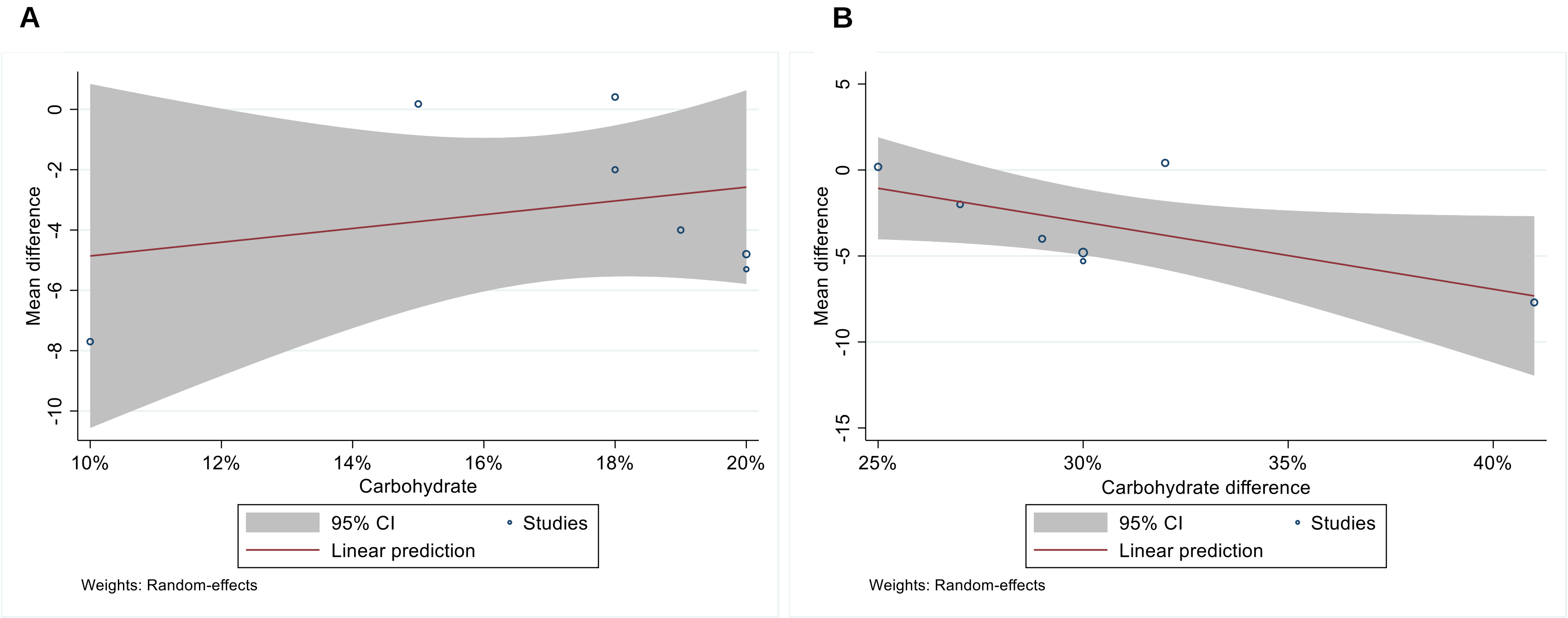

Supplementary Figure 40. Meta-regression of mean difference in coefficient of variation (%) for carbohydrate-restricted versus non-carbohydrate-restricted diets with (A) carbohydrate % of total energy intake (p=0.539) and (B) carbohydrate difference between intervention and control group (p=0.058) as effect modifiers.

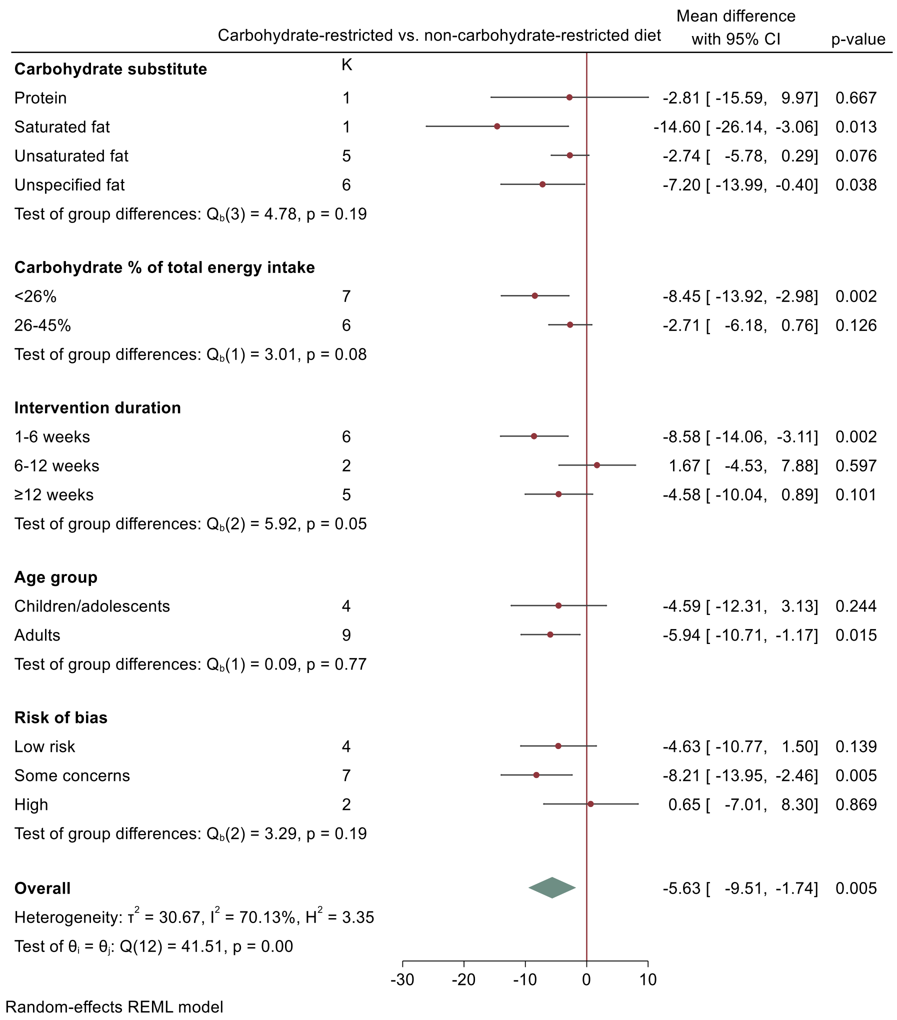

Supplementary Figure 41. Subgroup analyses for summary of mean difference in insulin dose (U/day) for carbohydrate-restricted versus non-carbohydrate-restricted diets.

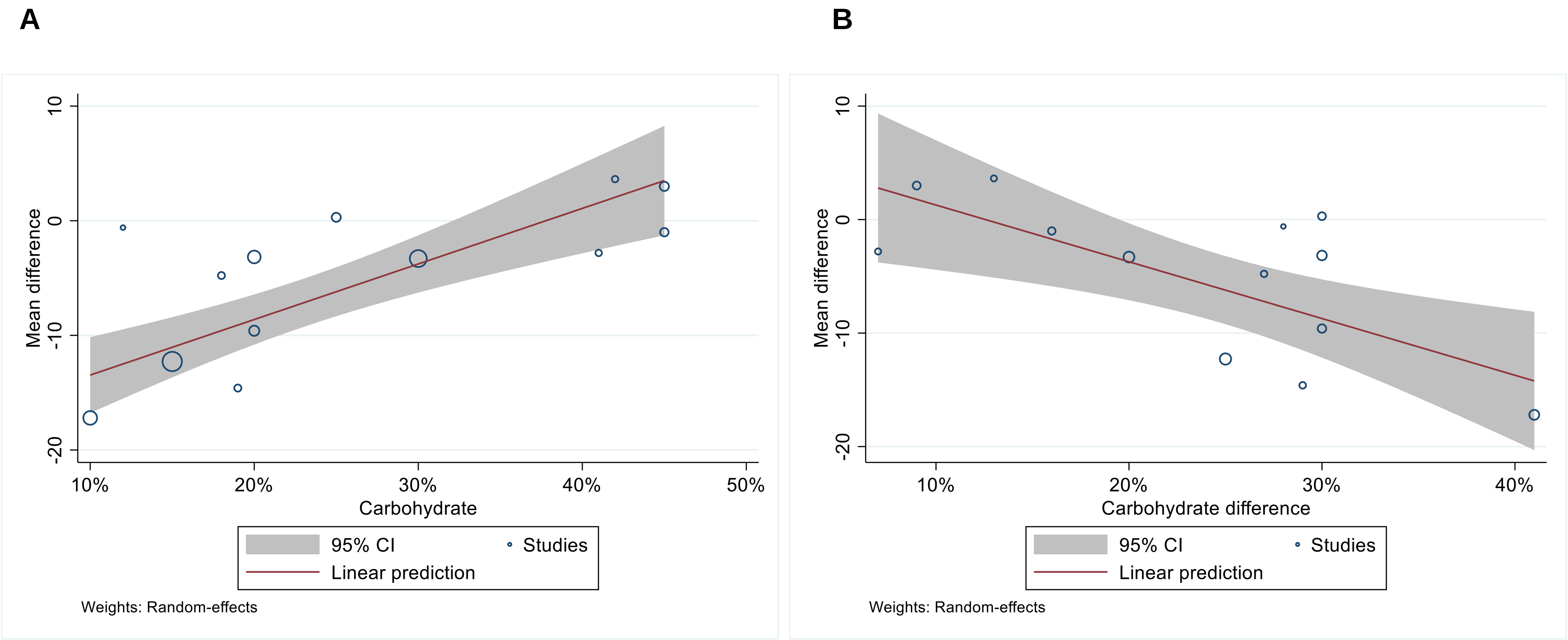

Supplementary Figure 42. Meta-regression of mean difference in insulin dose (U/day) for carbohydrate-restricted versus non-carbohydrate-restricted diets with (A) carbohydrate % of total energy intake ($p<0.001$) and (B) carbohydrate difference between intervention and control group ($p=0.003$) as effect modifiers.

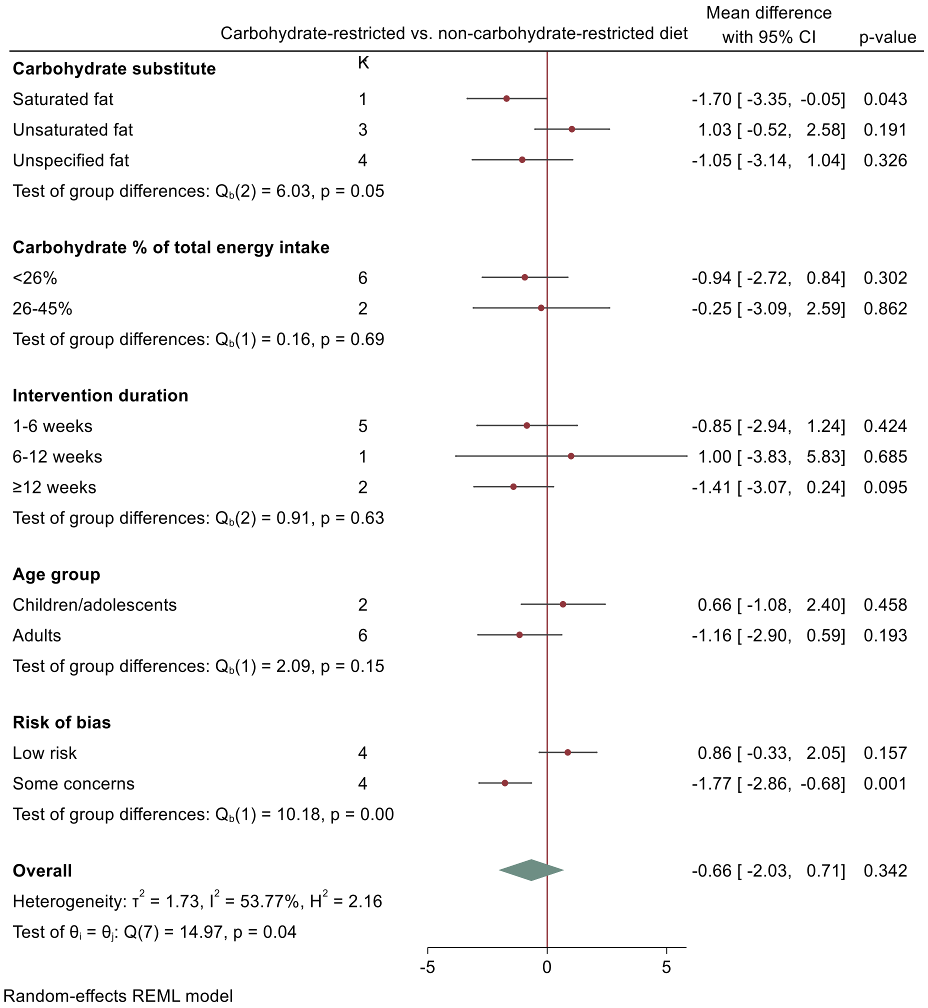

Supplementary Figure 43. Subgroup analyses for summary of mean difference in time below range (%) for carbohydrate-restricted versus non-carbohydrate-restricted diets.

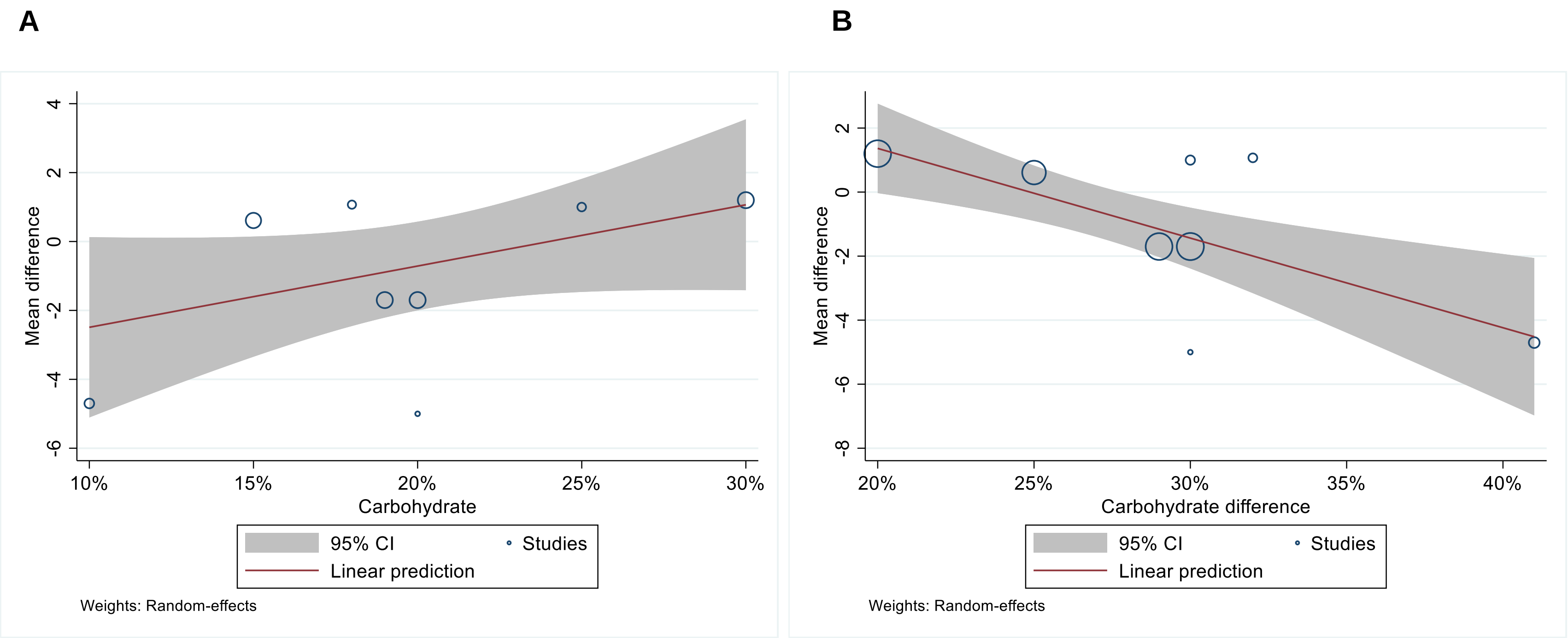

Supplementary Figure 44. Meta-regression of mean difference in time below range (%) for carbohydrate-restricted versus non-carbohydrate-restricted diets with (A) carbohydrate % of total energy intake (p=0.113) and (B) carbohydrate difference between intervention and control group (p=0.001) as effect modifiers.

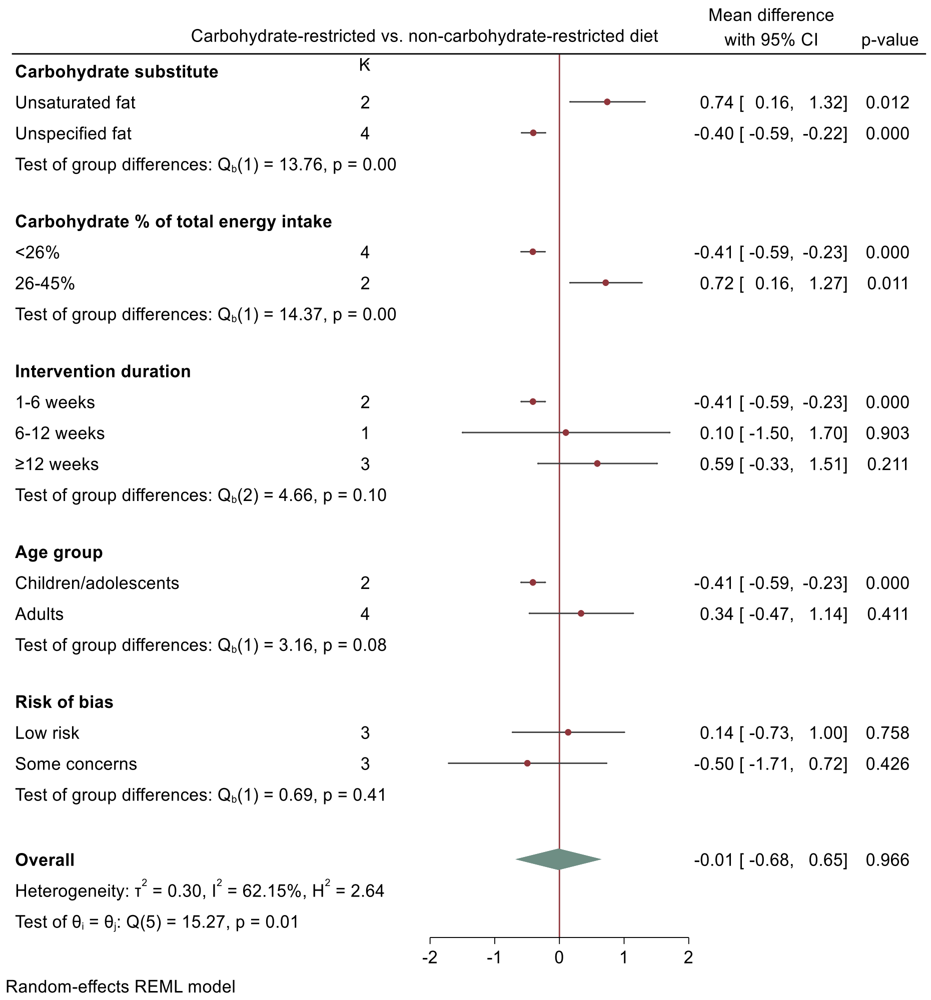

Supplementary Figure 45. Subgroup analyses for summary of mean difference in body mass index (kg/m^2) for carbohydrate-restricted versus non-carbohydrate-restricted diets.

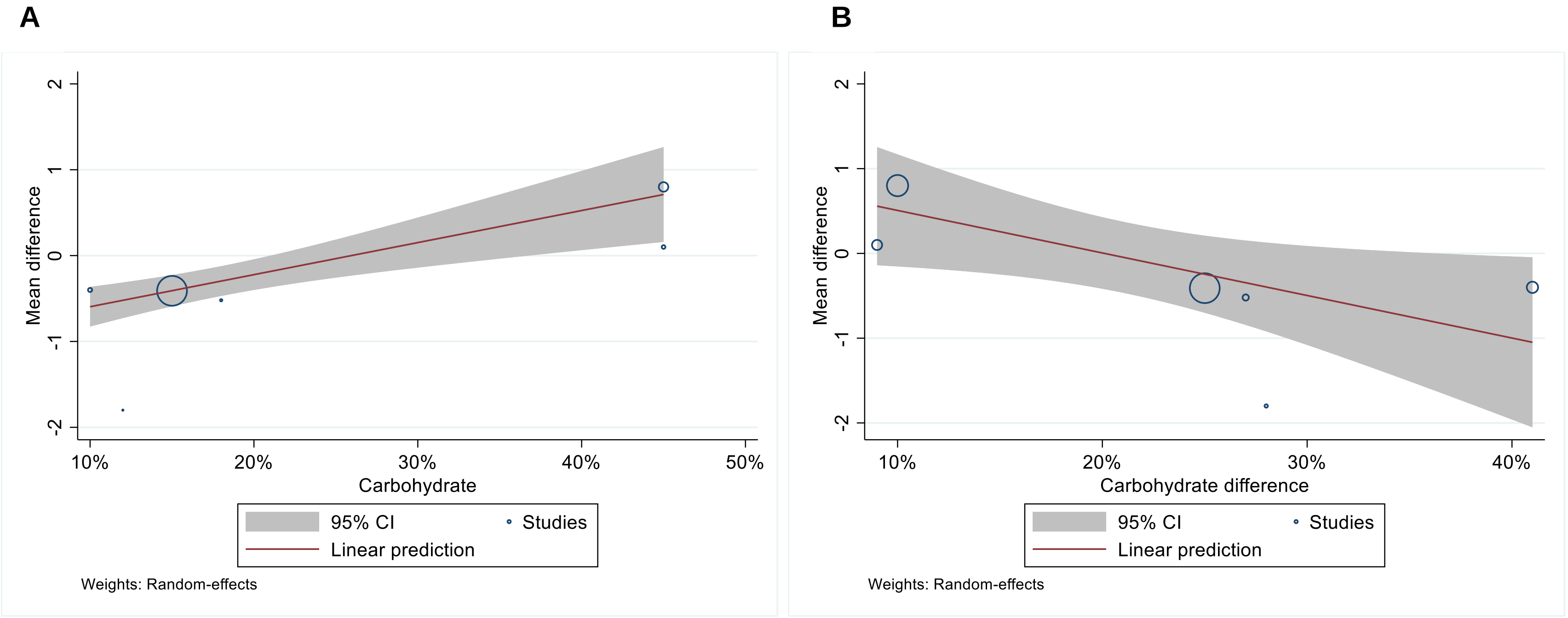

Supplementary Figure 46. Meta-regression of mean difference in body mass index (kg/m^2) for carbohydrate-restricted versus non-carbohydrate-restricted diets with (A) carbohydrate % of total energy intake (p<0.001) and (B) carbohydrate difference between intervention and control group (p=0.032) as effect modifiers.

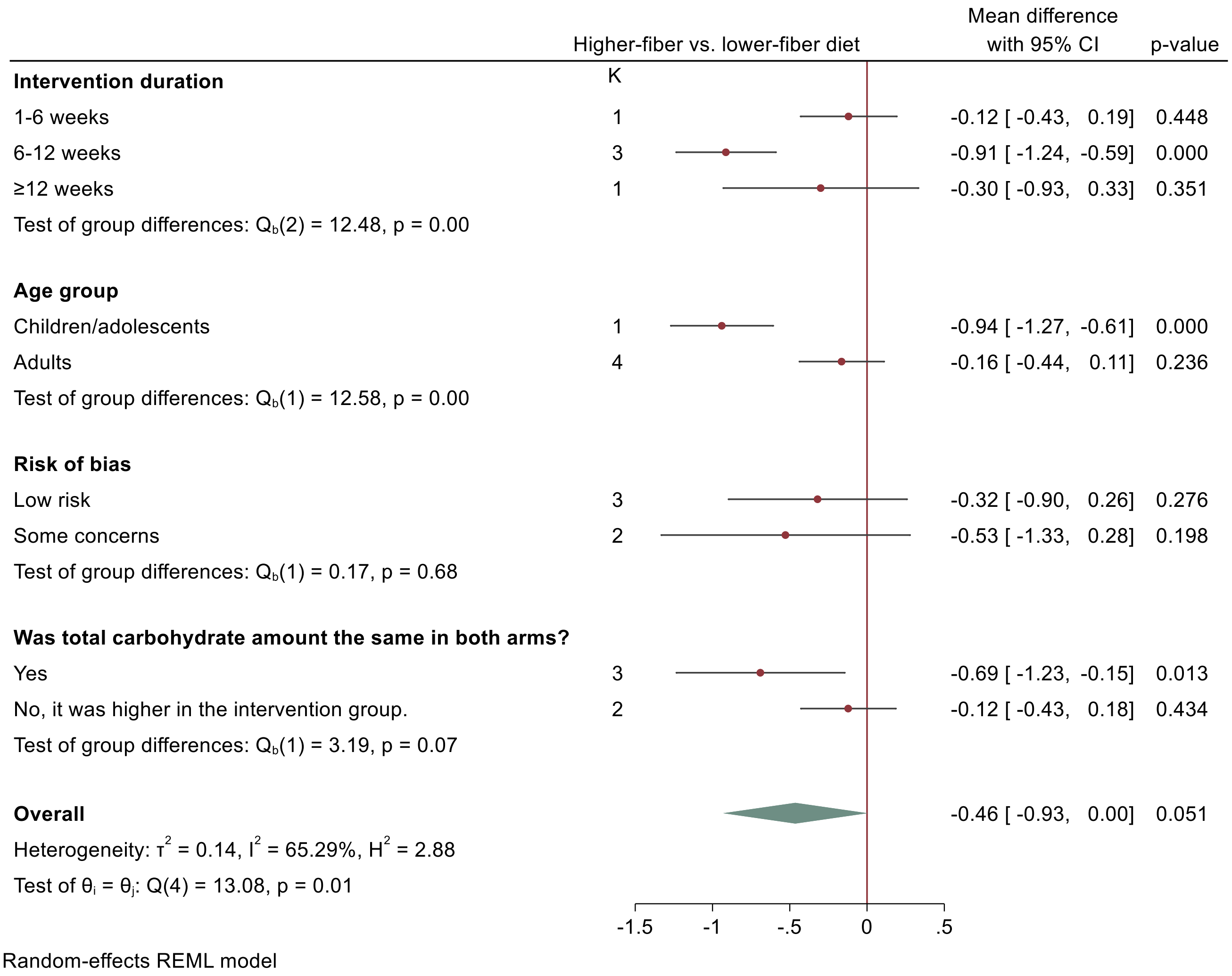

Supplementary Figure 47. Subgroup analyses for summary of mean difference in HbA1c (%) for higher-fiber versus lower-fiber diets.

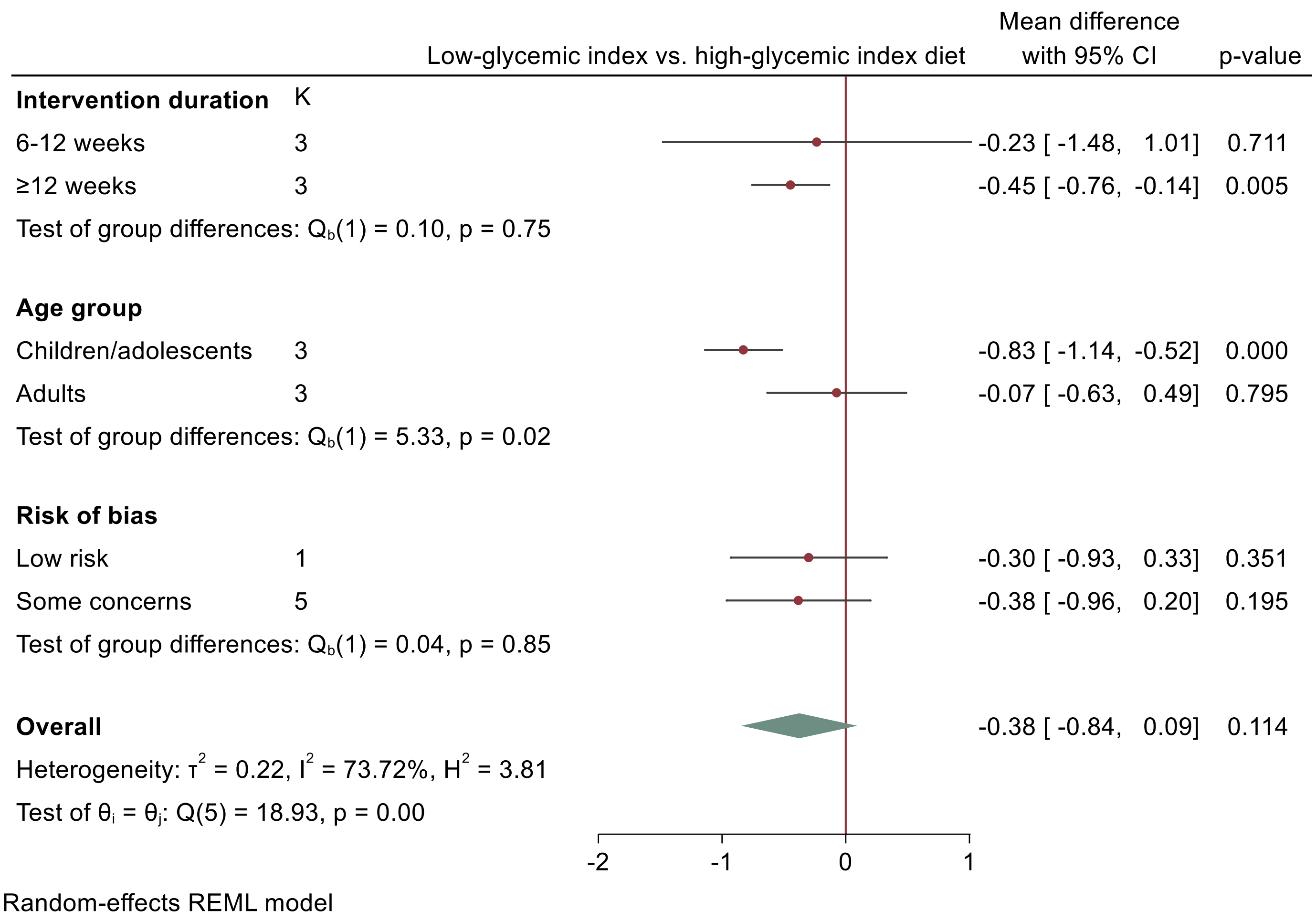

Supplementary Figure 48. Subgroup analyses for summary of mean difference in HbA1c (%) for low-glycemic index versus high-glycemic index diets.

Supplementary references

1. Al-Sari N, Schmidt S, Suvitaival T, Kim M, Trost K, Ranjan AG, et al. Changes in the lipidome in type 1 diabetes following low carbohydrate diet: Post-hoc analysis of a randomized crossover trial. *Endocrinol Diabetes Metab*. 2021;4(2):e00213.
2. Asayama K, Miyao A, Bradley CA. Effect of high-carbohydrate diet on lipid metabolism in young type I diabetics: Transient changes in high-density lipoprotein2 and lipoprotein lipase. *Acta Paediatr Jpn (Overseas Ed*. 1986;28(4):616–23.
3. Assor E, Marcon MA, Hamilton N, Fry M, Cooper T, Mahmud FH. Design of a dietary intervention to assess the impact of a gluten-free diet in a population with type 1 Diabetes and Celiac Disease. *BMC Gastroenterol*. 2015 Dec 21;15(1):181.
4. Bassir L, Amani R, Khaneh Masjedi M, Ahangarpor F. Relationship between dietary patterns and dental health in type i diabetic children compared with healthy controls. *Iran Red Crescent Med J*. 2014;16(1).
5. Bentaleb M, Sersar I, Bencharif M, Lahlah N, Bendjama RFN, Djaafri Z. Type 1 diabetes mellitus and Ramadan fasting: A case of adolescents and adults from Algeria. *Nutr Clin Metab*. 2023;37(4):214–20.
6. Bielefeld D, Grafenauer S, Rangan A. The Effects of Legume Consumption on Markers of Glycaemic Control in Individuals with and without Diabetes Mellitus: A Systematic Literature Review of Randomised Controlled Trials. *Nutrients*. 2020 Jul 17;12(7):2123.
7. Birkbeck JA, Truswell AS, Thomas BJ. Current practice in dietary management of diabetic children. A transatlantic comparison. *Arch Dis Child*. 1976;51(6):467–70.
8. Br, -Miller J, Hayne S, Petocz P, Colagiuri S. Low-glycemic index diets in the management of diabetes: a meta-analysis of randomized controlled trials. *Diabetes Care*. 2003;26(8):2261–7.
9. Buyken AE, Toeller M, Heitkamp G, Irsigler K, Holler C, Santeusanio F, et al. Carbohydrate sources and glycaemic control in Type 1 diabetes mellitus. *Diabet Med*. 2000 May 24;17(5):351–9.
10. Buyken AE, Toeller M, Heitkamp G, Irsigler K, Holler C, Santeusanio F, et al. Carbohydrate sources and glycaemic control in type 1 diabetes mellitus. *Diabet Med*. 2000;17(5):351–9.
11. Cherubini V, Marino M, Marigliano M, Maffeis C, Zanfardino A, Rabbone I, et al. Rethinking carbohydrate intake and time in range in children and adolescents with type 1 diabetes. *Nutrients*. 2021;13(11).
12. Chiavaroli L, Lee D, Ahmed A, Cheung A, Khan TA, Blanco S, et al. Effect of low glycaemic index or load dietary patterns on glycaemic control and cardiometabolic risk factors in diabetes: systematic review and meta-analysis of randomised controlled trials. *BMJ*. 2021 Aug 4;374:n1651.
13. Chiplonkar S, Kajale N, Lohiya N, Parthasarhty L, Khadilkar V, Khadilkar A. Dietary patterns in underprivileged Indian children and adolescents with type 1 diabetes. *Curr Nutr Food Sci*. 2020;16(6):874–83.
14. De Castro JM, Bellisle F, Dalix AM, Slama G. The effect of Type I diabetes on the eating patterns of free-living French: A diet diary study. *Physiol Behav*. 2002;75(4):583–92.
15. Dingena CF, Arofikina D, Campbell MD, Holmes MJ, Scott EM, Zulyniak MA. Nutritional and Exercise-Focused Lifestyle Interventions and Glycemic Control in Women with Diabetes in Pregnancy: A Systematic Review and Meta-Analysis of Randomized Clinical Trials. *Nutrients*. 2023;15(2).

16. Dowis K, Banga S. The Potential Health Benefits of the Ketogenic Diet: A Narrative Review. *Nutrients*. 2021;13(5):13.
17. Dube MC, Lavoie C, Galibois I, Weisnagel SJ. Nutritional strategies to prevent hypoglycemia at exercise in diabetic adolescents. *Diabetes Technol Ther*. 2013;15:S103–4.
18. Dussol B, Iovanna C, Raccah D, Darmon P, Morange S, Vague P, et al. A randomized trial of low-protein diet in type 1 and in type 2 diabetes mellitus patients with incipient and overt nephropathy. *J Ren Nutr*. 2005;15(4):398–406.
19. Dyson P. Very low carbohydrate ketogenic diets and diabetes. *Pract Diabetes*. 2020;37(4):121–6.
20. Dyson PA, Twenefour D, Breen C, Duncan A, Elvin E, Goff L, et al. Diabetes <scp>UK</scp> evidence-based nutrition guidelines for the prevention and management of diabetes. *Diabet Med*. 2018 May 16;35(5):541–7.
21. Erbe JK. Low glycemic index diets for the management of diabetes. *Am Fam Physician*. 2009;80(9):941.
22. Feinman RD, Pogozelski WK, Astrup A, Bernstein RK, Fine EJ, Westman EC, et al. Dietary carbohydrate restriction as the first approach in diabetes management: Critical review and evidence base. *Nutrition*. 2015;31(1):1–13.
23. Gillingham MB, Marak MC, Riddell MC, Calhoun P, Gal RL, Patton SR, et al. The Association Between Diet Quality and Glycemic Outcomes Among People with Type 1 Diabetes. *Curr Dev Nutr*. 2024 Apr;8(4):102146.
24. Grabia M, Puścion-Jakubik A, Markiewicz-żukowska R, Bielecka J, Mielech A, Nowakowski P, et al. Adherence to mediterranean diet and selected lifestyle elements among young women with type 1 diabetes mellitus from Northeast Poland: A case-control covid-19 survey. *Nutrients*. 2021;13(4).
25. Granado-Casas M, Sola I, Hernandez M, Rojo-Lopez MI, Julve J, Mauricio D. Effectiveness of medical nutrition therapy in adolescents with type 1 diabetes: a systematic review. *Nutr Diabetes*. 2022;12(1):24.
26. H, u D, Piotrowski M. Nutrition Interventions in Pediatric Patients with Type 1 Diabetes: An Evidence Analysis Center Scoping Review. *J Acad Nutr Diet*. 2022;122(2):424–31.
27. Hancock M, Burns K, Gan SK, Chew GT. Low-carbohydrate diets in type 1 diabetes: balancing benefits and risks. *Curr Opin Endocrinol Diabetes Obes*. 2023;30(2):113–22.
28. Hollenbeck CB, Connor WE, Riddle MC. The effects of a high-carbohydrate low-fat cholesterol-restricted diet on plasma lipid, lipoprotein, and apoprotein concentrations in insulin-dependent (Type I) diabetes mellitus. *Metabolism*. 1985;34(6):559–66.
29. Hollenbeck CB, Riddle MC, Connor WE, Leklem JE. The effects of subject-selected high carbohydrate, low fat diets on glycemic control in insulin dependent diabetes mellitus. *Am J Clin Nutr*. 1985;41(2):293–8.
30. Kaukinen K, Salmi J, Lahtela J, Siljamaki-Ojansuu U, Koivisto AM, Oksa H, et al. No effect of gluten-free diet on the metabolic control of type 1 diabetes in patients with diabetes and celiac disease. Retrospective and controlled prospective survey. *Diabetes Care*. 1999;22(10):1747–8.
31. Kietsiriroje N, Shah H, Zare M, O'Mahoney LL, West DJ, Pearson SM, et al. Dietary fat intake is associated with insulin resistance and an adverse vascular profile in patients with T1D: a pooled analysis. *Eur J Nutr*. 2023;62(3):1231–8.

32. Klupa T, Benbenek-Klupa T, Matejko B, Mrozinska S, Malecki MT. The impact of a pure protein load on the glucose levels in type 1 diabetes patients treated with insulin pumps. *Int J Endocrinol*. 2015;2015.
33. Kochar R, Herold KC. Nutrition: Prevention of T1DM: Feeding the ultimate goal. *Nat Rev Endocrinol*. 2011;7(3):132–4.
34. Kopple JD. Do low-protein diets retard the loss of kidney function in patients with diabetic nephropathy? *Am J Clin Nutr*. 2008;88(3):593–4.
35. Lampousi AM, Carlsson S, Löfvenborg JE. Dietary factors and risk of islet autoimmunity and type 1 diabetes: a systematic review and meta-analysis. *EBioMedicine*. 2021 Oct;72:103633.
36. Lariviere F, Chiasson JL, Schiffrin A, Taveroff A, Hoffer LJ. Effects of dietary protein restriction on glucose and insulin metabolism in normal and diabetic humans. *Metabolism*. 1994;43(4):462–7.
37. Lassenius MI, Mäkinen VP, Fogarty CL, Peräneva L, Jauhiainen M, Pussinen PJ, et al. Patients with type 1 diabetes show signs of vascular dysfunction in response to multiple high-fat meals. *Nutr Metab*. 2014;11(1).
38. Lehmann V, Zueger T, Zeder A, Scott S, Bally L, Laimer M, et al. Lower daily carbohydrate intake is associated with improved glycemic control in adults with type 1 diabetes using a hybrid closed-loop system. *Diabetes Care*. 2020;43(12):3102–5.
39. Lejk A, Chrzanowski J, Cieślak A, Fendler W, Myśliwiec M. Reduced Carbohydrate Diet Influence on Postprandial Glycemia—Results of a Short, CGM-Based, Interventional Study in Adolescents with Type 1 Diabetes. *Nutrients*. 2022;14(21).
40. Levran N, Levek N, Sher B, Gruber N, Afek A, Monsonego-Ornan E, et al. The Impact of a Low-Carbohydrate Diet on Micronutrient Intake and Status in Adolescents with Type 1 Diabetes. *Nutrients*. 2023;15(6).
41. Loghmani E, Rickard K, Washburne L, Vandagriff J, Fineberg N, Golden M. Glycemic response to sucrose-containing mixed meals in diets of children of with insulin-dependent diabetes mellitus. *J Pediatr*. 1991;119(4):531–7.
42. MacLeod J, Franz MJ, H, u D, Gradwell E, Brown C, et al. Academy of Nutrition and Dietetics Nutrition Practice Guideline for Type 1 and Type 2 Diabetes in Adults: Nutrition Intervention Evidence Reviews and Recommendations. *J Acad Nutr Diet*. 2017;117(10):1637–58.
43. Mahmud FH, De Melo EN, Noordin K, Assor E, Sahota K, Davies-Shaw J, et al. The Celiac Disease and Diabetes-Dietary Intervention and Evaluation Trial (CD-DIET) protocol: a randomised controlled study to evaluate treatment of asymptomatic coeliac disease in type 1 diabetes. *BMJ Open*. 2015;5(5):e008097.
44. Mahon JL, Dupre J. The limitations of clinical trials for prevention of IDDM. *Diabetes Care*. 1997;20(6):1027-1033.
45. Marquard J, Stahl A, Lerch C, Wolters M, Grotzke-Leweling M, Mayatepek E, et al. A prospective clinical pilot-trial comparing the effect of an optimized mixed diet versus a flexible low-glycemic index diet on nutrient intake and HbA(1c) levels in children with type 1 diabetes. Vol. 24, Journal of pediatric endocrinology & metabolism : JPEM. Germany; 2011. p. 441–7.
46. McAulay V, Farguson SC, Frier BM. Post-prandial administration of insulin lispro with a high fat meal minimizes risk of hypoglycaemia in Type 1 diabetes [3]. *Diabet Med*. 2004;21(8):953–4.

47. Muntis FR, Smith-Ryan AE, Crandell J, Evenson KR, Maahs DM, Seid M, et al. A High Protein Diet Is Associated with Improved Glycemic Control Following Exercise among Adolescents with Type 1 Diabetes. *Nutrients*. 2023;15(8).
48. Neuman V, Plachy L, Pruhova S, Kolouskova S, Petruzelkova L, Obermannova B, et al. Low-carbohydrate diet among children with type 1 diabetes: A multi-center study. *Nutrients*. 2021;13(11).
49. Øverby NC, Flaaten V, Veierød MB, Bergstad I, Margeirsdottir HD, Dahl-Jørgensen K, et al. Children and adolescents with type 1 diabetes eat a more atherosclerosis-prone diet than healthy control subjects. *Diabetologia*. 2007;50(2):307–16.
50. Pan Y, Guo LL, Jin HM. Low-protein diet for diabetic nephropathy: a meta-analysis of randomized controlled trials. *Am J Clin Nutr*. 2008;88(3):660–6.
51. Passali M, Josefsen K, Frederiksen JL, Antvorskov JC. Current evidence on the efficacy of gluten-free diets in multiple sclerosis, psoriasis, type 1 diabetes and autoimmune thyroid diseases. *Nutrients*. 2020;12(8):1–26.
52. Reynolds AN, Akerman AP, Mann J. Dietary fibre and whole grains in diabetes management: Systematic review and meta-analyses. Ma RCW, editor. *PLOS Med*. 2020 Mar 6;17(3):e1003053.
53. Roskjær AB, Andersen JR, Ronneby H, Damm P, Mathiesen ER. Dietary advices on carbohydrate intake for pregnant women with type 1 diabetes. *J Matern Neonatal Med*. 2015;28(2):229–33.
54. Rovner AJ, Nansel TR, Gellar L. The Effect of a Low-Glycemic Diet vs a Standard Diet on Blood Glucose Levels and Macronutrient Intake in Children with Type 1 Diabetes. *J Am Diet Assoc*. 2009 Feb;109(2):303–7.
55. Sanz-París A, Matía-Martín P, Martín-Palmero Á, Gómez-Candela C, Camprubi Robles M. Diabetes-specific formulas high in monounsaturated fatty acids and metabolic outcomes in patients with diabetes or hyperglycaemia. A systematic review and meta-analysis. *Clin Nutr*. 2020 Nov;39(11):3273–82.
56. Seckiner S, Bas M, Simsir IY, Ozgur S, Akcay Y, Aslan CG, et al. Effects of Dietary Carbohydrate Concentration and Glycemic Index on Blood Glucose Variability and Free Fatty Acids in Individuals with Type 1 Diabetes. *Nutrients*. 2024 May 2;16(9):1383.
57. Seid H, Rosenbaum M. Low Carbohydrate and Low-Fat Diets: What We Don’t Know and Why We Should Know It. *Nutrients*. 2019 Nov 12;11(11):2749.
58. Soderstrom H, Cervin M, Dereke J, Hillman M, Tiberg I, Norstrom F, et al. Does a gluten-free diet lead to better glycemic control in children with type 1 diabetes? Results from a feasibility study and recommendations for future trials. *Contemp Clin trials Commun*. 2022;26:100893.
59. Sohouli MH, Mirmiran P, Seraj SS, Kutbi E, Alkahmous HAM, Almuqayyid F, et al. Impact of low-protein diet on cardiovascular risk factors and kidney function in diabetic nephropathy: A systematic review and meta-analysis of randomized-controlled trials. *Diabetes Res Clin Pract*. 2022;191:110068.
60. Souto DL, Dantas JR, Oliveira MMDS, Rosado EL, Luiz RR, Zajdenverg L, et al. Does sucrose affect the glucose variability in patients with type 1 diabetes? a pilot crossover clinical study. *Nutrition*. 2018 Nov;55–56:179–84.
61. Thackrey E, Chen J, Martino CR, Preda V. The effects of diet on weight and metabolic outcomes in patients with double diabetes: A systematic review. *Nutrition*. 2022;94:111536.

62. Thomas DE, Elliott EJ. The use of low-glycaemic index diets in diabetes control. *Br J Nutr*. 2010;104(6):797–802.
63. Thomas D, Elliott EJ. Low glycaemic index, or low glycaemic load, diets for diabetes mellitus. *Cochrane Database Syst Rev*. 2009 Jan 21;(1):CD006296.
64. Thomson RL, Brown JD, Oakey H, Palmer K, Ashwood P, Penno MAS, et al. Dietary patterns during pregnancy and maternal and birth outcomes in women with type 1 diabetes: the Environmental Determinants of Islet Autoimmunity (ENDIA) study. *Diabetologia*. 2024 Nov 2;67(11):2420–32.
65. Tromba V, Silvestri F. Vegetarianism and type 1 diabetes in children. *Metab open*. 2021;11:100099.
66. Turton JL, Raab R, Rooney KB. Low-carbohydrate diets for type 1 diabetes mellitus: A systematic review. *PLoS ONE [Electronic Resour*. 2018;13(3):e0194987.
67. Valerio G, Spadaro R, Iafusco D, Lombardi F, del Puente A, Esposito A, et al. The influence of gluten free diet on quantitative ultrasound of proximal phalanxes in children and adolescents with type 1 diabetes mellitus and celiac disease. *Bone*. 2008 Aug;43(2):322–6.
68. Wilson D, Chase HP, Kollman C, Xing D, Caswell K, Tansey M, et al. Low-fat vs. high-fat bedtime snacks in children and adolescents with type 1 diabetes. *Pediatr Diabetes*. 2008 Aug;9(4pt1):320–5.
69. Zafar MI, Mills KE, Zheng J, Regmi A, Hu SQ, Gou L, et al. Low-glycemic index diets as an intervention for diabetes: a systematic review and meta-analysis. *Am J Clin Nutr*. 2019 Oct;110(4):891–902.
70. Agrawal RP, Jain S, Shah S, Chopra A, Agarwal V. Effect of camel milk on glycemic control and insulin requirement in patients with type 1 diabetes: 2-years randomized controlled trial. *Eur J Clin Nutr*. 2011;65(9):1048–52.
71. Amiel S, Beveridge S, Bradley C, Gianfrancesco C, Heller S, James P, et al. Training in flexible, intensive insulin management to enable dietary freedom in people with type 1 diabetes: Dose adjustment for normal eating (DAFNE) randomised controlled trial. *Br Med J*. 2002;325(7367):746–9.
72. Andrade R, Laitano O, Meyer F. Effect of hydration with carbohydrates on the glycemic response in type I diabetics during exercise. *Rev Bras Med do esporte*. 2005;11(1):61-70.
73. Barouti AA, Bjorklund A, Catrina SB, Brismar K, Rajamand Ekberg N. Effect of Isocaloric Meals on Postprandial Glycemic and Metabolic Markers in Type 1 Diabetes-A Randomized Crossover Trial. *Nutrients*. 2023;15(14).
74. Bozzetto L, Alderisio A, Giorgini M, Barone F, Giacco A, Riccardi G, et al. Extra-Virgin Olive Oil Reduces Glycemic Response to a High-Glycemic Index Meal in Patients With Type 1 Diabetes: A Randomized Controlled Trial. *Diabetes Care*. 2016;39(4):518–24.
75. Campbell MD, Walker M, Ajjan RA, Birch KM, Gonzalez JT, West DJ. An additional bolus of rapid-acting insulin to normalise postprandial cardiovascular risk factors following a high-carbohydrate high-fat meal in patients with type 1 diabetes: A randomised controlled trial. *Diabetes Vasc Dis Res*. 2017 Jul 21;14(4):336–44.
76. Cohen E, Tsoukas MA, Legault L, Vallis M, Von Oettingen JE, Palisaitis E, et al. Simple meal announcements and pramlintide delivery versus carbohydrate counting in type 1 diabetes with automated fast-acting insulin aspart delivery: a randomised crossover trial in Montreal, Canada. *Lancet Digit Heal*. 2024 Jul;6(7):e489–99.

77. Cordon NM, Smart CEM, Smith GJ, Davis EA, Jones TW, Seckold R, et al. The relationship between meal carbohydrate quantity and the insulin to carbohydrate ratio required to maintain glycaemia is non-linear in young people with type 1 diabetes: A randomized crossover trial. *Diabet Med*. 2022;39(2):e14675.
78. Dzygalo K, Indulska K, Szypowska A. Pure-protein load for children with type 1 diabetes: is any additional insulin needed? A randomized controlled study. *Acta Diabetol*. 2023;60(3):337–43.
79. Elbarbary NS, Ismail EAR. Glycemic control during Ramadan fasting in adolescents and young adults with type 1 diabetes on MiniMed TM 780G advanced hybrid closed-loop system: A randomized controlled trial. *Diabetes Res Clin Pract*. 2022;191:110045.
80. Erdal B, Caferoglu Z, Hatipoglu N. The comparison of two mealtime insulin dosing algorithms for high and low glycaemic index meals in adolescents with type 1 diabetes. *Diabet Med*. 2021;38(3):e14444.
81. Garcia A, Moscardo V, Ramos-Prol A, Diaz J, Boronat M, Bondia J, et al. Effect of meal composition and alcohol consumption on postprandial glucose concentration in subjects with type 1 diabetes: a randomized crossover trial. *BMJ Open Diabetes Res Care*. 2021;9(1):10.
82. Gilbertson HR, Thornburn AW, Br, -miller JC, Chondros P, Werther GA. Effect of low glycaemic-index dietary advice on dietary quality and food choice in children with type 1 diabetes. *Am J Clin Nutr*. 2003;77:83-90.
83. Gingras V, Bonato L, Messier V, Roy-Fleming A, Smaoui MR, Ladouceur M, et al. Impact of macronutrient content of meals on postprandial glucose control in the context of closed-loop insulin delivery: A randomized cross-over study. *Diabetes Obes Metab*. 2018;20(11):2695–9.
84. Goulet-Gelinas L, Saade MB, Suppere C, Fortin A, Messier V, Taleb N, et al. Comparison of two carbohydrate intake strategies to improve glucose control during exercise in adolescents and adults with type 1 diabetes. *Nutr Metab Cardiovasc Dis*. 2021;31(4):1238–46.
85. Gümüş AB, Keser A, Şiklar Z, Berberoğlu M. The impact of high-fat and high-protein meal of adolescents with type 1 diabetes mellitus receiving intensive insulin therapy on postprandial blood glucose level: a randomized, crossover, breakfast study. *Int J Diabetes Dev Ctries*. 2021;41(2):249–58.
86. Kaufman FR, Halvorson M, Kaufman ND. A randomized, blinded trial of uncooked cornstarch to diminish nocturnal hypoglycemia at diabetes camp. *Diabetes Res Clin Pract*. 1995;30(3):205–9.
87. Kaya N, Kurtoglu S, Gokmen Ozel H. Does meal-time insulin dosing based on fat-protein counting give positive results in postprandial glycaemic profile after a high protein-fat meal in adolescents with type 1 diabetes: a randomised controlled trial. *J Hum Nutr Diet*. 2020;33(3):396–403.
88. Kowalczyk-Korcz E, Dymińska M, Szypowska A. Super Bolus—A Remedy for a High Glycemic Index Meal in Children with Type 1 Diabetes on Insulin Pump Therapy?—A Randomized, Double-Blind, Controlled Trial. *Nutrients*. 2024 Jan 16;16(2):263.
89. Krarup-Hansen A, Lauritzen T, Christiansen JS, Svendsen PA, Deckert T. Diet versus average Danish food in insulin-dependent diabetes mellitus. An evaluation during treatment with an artificial betacell. *Scand J Clin Lab Investig*. 1982;42(8):603–6.

90. Krebs JD, Parry Strong A, Cresswell P, Reynolds AN, Hanna A, Haeusler S. A randomised trial of the feasibility of a low carbohydrate diet vs standard carbohydrate counting in adults with type 1 diabetes taking body weight into account. *Asia Pac J Clin Nutr*. 2016;25(1):78–84.
91. Kristensen KB, Ranjan AG, McCarthy OM, Holst JJ, Bracken RM, Nørgaard K, et al. Effects of a Low-Carbohydrate-High-Protein Pre-Exercise Meal in Type 1 Diabetes—a Randomized Crossover Trial. *J Clin Endocrinol Metab*. 2023 Dec 21;109(1):208–16.
92. Mohammed MHH, Al-Qahtani MHH, Takken T. Effects of 12 weeks of recreational football (soccer) with caloric control on glycemia and cardiovascular health of adolescent boys with type 1 diabetes. *Pediatr Diabetes*. 2021;22(4):625–37.
93. Muntis FR, Crandell JL, Evenson KR, Maahs DM, Seid M, Shaikh SR, et al. Pre-exercise protein intake is associated with reduced time in hypoglycaemia among adolescents with type 1 diabetes. *Diabetes Obes Metab*. 26(4):1366–75.
94. Nansel TR, Lipsky LM, Liu A. Greater diet quality is associated with more optimal glycemic control in a longitudinal study of youth with type 1 diabetes. *Am J Clin Nutr*. 2016;104(1):81–7.
95. Pańkowska E, Błazik M, Groele L. Does the Fat-Protein Meal Increase Postprandial Glucose Level in Type 1 Diabetes Patients on Insulin Pump: The Conclusion of a Randomized Study. *Diabetes Technol Ther*. 2012 Jan;14(1):16–22.
96. Papakonstantinou E, Papavasiliou K, Maouri C, Magriplis E, Pappas S, Bousboulas S, et al. Postprandial glucose response after the consumption of three mixed meals based on the carbohydrate counting method in adults with type 1 diabetes. A randomized crossover trial. *Clin Nutr ESPEN*. 2019;31:48–55.
97. Paterson MA, Smart CEM, Howley P, Price DA, Foskett DC, King BR. High-protein meals require 30% additional insulin to prevent delayed postprandial hyperglycaemia. *Diabet Med*. 2020;37(7):1185–91.
98. Petrovski G, Campbell J, Pasha M, Day E, Hussain K, Khalifa A, et al. Simplified Meal Announcement Versus Precise Carbohydrate Counting in Adolescents With Type 1 Diabetes Using the MiniMed 780G Advanced Hybrid Closed Loop System: A Randomized Controlled Trial Comparing Glucose Control. *Diabetes Care*. 2023;46(3):544–50.
99. Picard K, Senior PA, Adame Perez S, Jindal K, Richard C, Mager DR. Low Mediterranean Diet scores are associated with reduced kidney function and health related quality of life but not other markers of cardiovascular risk in adults with diabetes and chronic kidney disease. *Nutr Metab Cardiovasc Dis*. 2021;31(5):1445-1453.
100. Pozzilli P, Raz I, Peled D, Elias D, Avron A, Tamir M, et al. Evaluation of long-term treatment effect in a type 1 diabetes intervention trial: differences after stimulation with glucagon or a mixed meal. *Diabetes Care*. 2014;37(5):1384–91.
101. Sels JPJE, Postmes TJL, Nieman F, Ebert R, Wolffenbuttel BHR, Kruseman ACN. Effects of guar bread in type 1 and type 2 diabetes mellitus. *Eur J Intern Med*. 1993;4(3):193–200.
102. Sharma R, Upadhyay B, Lal N, Sagar R, Jain V. Carbohydrate Counting vs. Fixed Meal Plan in Indian Children with Type 1 Diabetes Mellitus: A Randomized Controlled Trial. *Indian J Pediatr*. 2023;
103. Smart CEM, Evans M, O'Connell SM, McElduff P, Lopez PE, Jones TW, et al. Both Dietary Protein and Fat Increase Postprandial Glucose Excursions in Children With Type 1 Diabetes, and the Effect Is Additive. *Diabetes Care*. 2013 Dec 1;36(12):3897–902.

104. Sterner Isaksson S, Bensow Bacos M, Eliasson B, Thors Adolfsson E, Rawshani A, Lindblad U, et al. Effects of nutrition education using a food-based approach, carbohydrate counting or routine care in type 1 diabetes: 12 months prospective randomized trial. *BMJ Open Diabetes Res Care*. 2021;9(1):3.
105. Tandon A, Bhowmik E, Ali Z, Tripathi S, BK A, Chen Y, et al. Basic carbohydrate counting and glycemia in young people with type 1 diabetes in India: A randomized controlled trial. *Nutrition*. 2024 Mar;119:112318.
106. van der Hoogt M, van Dyk JC, Dolman RC, Pieters M. Protein and fat meal content increase insulin requirement in children with type 1 diabetes - Role of duration of diabetes. *J Clin Transl Endocrinol*. 2017;10:15–21.
107. Vetrani C, Bozzetto L, Giorgini M, Cavagnuolo L, Di Mattia E, Cipriano P, et al. Fibre-enriched buckwheat pasta modifies blood glucose response compared to corn pasta in individuals with type 1 diabetes and celiac disease: Acute randomized controlled trial. *Diabetes Res Clin Pract*. 2019;149:156–62.
108. Vuorinen-Markkola H, Sinisalo M, Koivisto VA. Guar gum in insulin-dependent diabetes: effects on glycemic control and serum lipoproteins. *Am J Clin Nutr*. 1992;56(6):1056–60.
109. Zavitsanou S, Massa J, Deshp, e S, Pinsker JE, Church MM, et al. The Effect of Two Types of Pasta Versus White Rice on Postprandial Blood Glucose Levels in Adults with Type 1 Diabetes: A Randomized Crossover Trial. *Diabetes Technol Ther*. 2019;21(9):485–92.
110. Belfort GP, De Padilha PC, Farias DR, Da Silva LBG, Dos Santos K, Gomes EDS, et al. Effect of the Dietary Approaches to Stop Hypertension (DASH) diet on the development of preeclampsia and metabolic outcomes in pregnant women with pre-existing diabetes mellitus: A randomised, controlled, single-blind trial. *J Nutr Sci*. 2023;12.
111. Campbell MD, Gonzalez JT, Rumbold PL, Walker M, Shaw JA, Stevenson EJ, et al. Comparison of appetite responses to high– and low–glycemic index postexercise meals under matched insulinemia and fiber in type 1 diabetes. *Am J Clin Nutr*. 2015 Mar;101(3):478–86.
112. Evans M, Smart CEM, Paramalingam N, Smith GJ, Jones TW, King BR, et al. Dietary protein affects both the dose and pattern of insulin delivery required to achieve postprandial euglycaemia in Type 1 diabetes: a randomized trial. *Diabet Med*. 2019;36(4):499–504.
113. Harray AJ, Binkowski S, Keating BL, Horowitz M, St, field S, et al. Effects of Dietary Fat and Protein on Glucoregulatory Hormones in Adolescents and Young Adults With Type 1 Diabetes. *J Clin Endocrinol Metab*. 2022;107(1):e205–13.
114. Lafrance L, Rabasa-Lhoret R, Poisson D, Ducros F, Chiasson JL. Effects of different glycaemic index foods and dietary fibre intake on glycaemic control in type 1 diabetic patients on intensive insulin therapy. *Diabet Med*. 1998;15(11):972–8.
115. Perrotti N, Santoro D, Genovese S, Giacco A, Rivellese A, Riccardi G. Effect of digestible carbohydrates on glucose control in insulin-dependent diabetic patients. *Diabetes Care*. 1984;7(4):354–9.
116. Ranjan A, Schmidt S, Damm-Frydenberg C, Steineck I, Clausen TR, Holst JJ, et al. Low-Carbohydrate Diet Impairs the Effect of Glucagon in the Treatment of Insulin-Induced Mild Hypoglycemia: A Randomized Crossover Study. *Diabetes Care*. 2017;40(1):132–5.
117. Simpson HCR, Mann JI, Chakrabarti R. Effect of high-fibre diet on haemostatic variables in diabetes. *Br Med J*. 1982;284(6329):1608.

118. Weiman DI, Mahmud FH, Clarke ABM, Assor E, McDonald C, Saibil F, et al. Impact of a Gluten-Free Diet on Quality of Life and Health Perception in Patients With Type 1 Diabetes and Asymptomatic Celiac Disease. *J Clin Endocrinol Metab*. 2021;106(5):e1984–92.
119. Fontvieille AM, Acosta M, Rizkalla SW, Bornet F, David P, Letanoux M, et al. A moderate switch from high to low glycaemic-index foods for 3 weeks improves the metabolic control of Type I (IDDM) diabetic subjects. *Diabetes, Nutr Metab - Clin Exp*. 1988;1(2):139–43.
120. Fontvieille AM, Rizkalla SW, Penfornis A, Acosta M, Bornet FR, Slama G. The use of low glycaemic index foods improves metabolic control of diabetic patients over five weeks. *Diabet Med*. 1992 Jun;9(5):444–50.
121. Petkova M, Boyanov M, Protich M, Krivoshiikova D, Vutova G. Long-term effects of dietary protein restriction and ACE-inhibitors on micro-albuminuria in normotensive type 1 diabetics. *Endokrinologya*. 2001;6(2):29–36.
122. Provenzano C, Vero R, Oliva A, Leto G, Puccio L, Vecci E, et al. Lispro insulin in type 1 diabetic patients on a Mediterranean or normal diet: a randomized, cross-over comparative study with regular insulin. *Diabetes Nutr Metab*. 2001;14(3):133–9.
123. Vigard T, Lindberg B, Elmstahl H, Bjorck I, Axelsson I. Low glycaemic index (GI) foods improve glucose control in children with type 1 diabetes mellitus. *J Pediatr Gastroenterol Nutr*. 2003;36(4):576.
124. Reece EA, Hagay Z, Gay LJ, O’Connor T, DeGennaro N, Homko CJ, et al. A randomized clinical trial of a fiber-enriched diabetic diet vs. the standard American Diabetes Association-recommended diet in the management of diabetes mellitus in pregnancy. *J Matern Investig*. 1995;5(1):8–12.
125. Dussol B, Iovanna C, Raccah D, Darmon P, Morange S, Vague P, et al. A randomized trial of low-protein diet in type 1 and in type 2 diabetes mellitus patients with incipient and overt nephropathy. *J Ren Nutr*. 2005;15(4):398–406.
126. Monnier LH, Blotman MJ, Colette C, Monnier MP, Mirouze J. Effects of dietary fibre supplementation in stable and labile insulin-dependent diabetics. *Diabetologia*. 1981;20(1):12–7.
127. Bando H, Ebe K, Muneta T, Bando M, Yonei Y. Glucose variability for a short period of low carbohydrate diet in diabetic patients with possible latent autoimmune diabetes in adults. *Int Med*. 2019;1(3):124.
128. Fontvieille AM, Rizkalla SW, Penfornis A, Acosta M, Bornet FR, Slama G. The use of low glycaemic index foods improves metabolic control of diabetic patients over five weeks. *Diabet Med*. 1992 Jun;9(5):444–50.
129. McCulloch DK, Mitchell RD, Ambler J, Tattersall RB. A prospective comparison of “conventional” and high carbohydrate/high fibre/low fat diets in adults with established type 1 (insulin-dependent) diabetes. *Diabetologia*. 1985;28(4):208–12.
130. Pedersen O, Hjollund E, Lindskov HO. Increased insulin receptor binding to monocytes from insulin-dependent diabetic patients after a low-fat, high-starch, high-fiber diet. *Diabetes Care*. 1982;5(3):284–91.
